# Supplementary material for: Assessing the characteristics of un- and under-vaccinated children in low- and middle-income countries: A multi-level cross-sectional study
Source: PLOS Glob Public Health. 2022 Apr 27;2(4):e0000244. doi: 10.1371/journal.pgph.0000244 (PMC10021434; doi:10.1371/journal.pgph.0000244)
Supplement: S1 Text — Supplementary file containing Tables A–O, Figs A-O and additional text referenced in the manuscript. (DOCX) [file pgph.0000244.s001.docx]

**Assessing the characteristics of un- and under-vaccinated children in low- and middle-income countries: A multi-level cross-sectional study**

C. Edson Utazi, Oliver Pannell, Justice M. K. Aheto, Adelle Wigley, Natalia Tejedor-Garavito, Josh Wunderlich, Brittany Hagedorn, Dan Hogan, Andrew J. Tatem

**Supplementary information**

This supplementary information accompanies the main manuscript. It contains additional information referenced in the main manuscript and is organized into three main sections:

1. Data section,
2. Analysis section, and
3. Results section.

**Data section**

As mentioned in the main manuscript, cross-sectional data on the outcome variables and some covariate information were obtained from various Demographic and Health Surveys (DHS). The DHS program utilizes a stratified, two-stage cluster sampling technique, involving selection of clusters (usually enumeration areas) from a national sampling frame in the first stage and households from within the selected clusters in the second stage, with stratification achieved by separating administrative (usually level 1, and in a few cases, level 2) areas in the country into urban and rural strata.

Wherever possible, we considered the reference category for each categorical DHS covariate to be the category that had the least likelihood of being vaccinated based on prior knowledge, except for cases where this category yielded a highly imprecise effect estimate due to a small sample size, in which case the largest category was used as the reference. In addition, for some countries, we combined the categories of some covariates which had small frequencies that resulted in imprecise effect estimates in bivariate and/or multivariate analysis, where this was feasible.

Other DHS variables considered but not included in the analyses due to the preponderance of missing data in these variables across all study countries were birth interval (preceding birth interval in months), maternal decision-making (whether or not mother decides health care, visits and purchases) and malnutrition variables (stunting, wasting and underweight). For each country, we first computed the proportion of missing values for each covariate and then further excluded variables with greater than 5% missing data from the analyses due to the possibility that missingness is biased in an unknown way. We note that information on some of the DHS covariates was not included in all the surveys analysed here, since DHS questionnaires can vary between countries or surveys. We also chose between similar covariates (e.g., maternal employment status and maternal occupation; knowledge of malaria and ownership of a bednet) where both of these were available for a given country, mostly based on completeness of data.

**Table A: Description and coding of outcome variables and covariate factors**

| **Variable** | **Description** |
| --- | --- |
| **Outcome variables**  Received DTP1  Received DTP3  Received MCV1 | No/don’t know – 0, reported by mother/ vaccination date on card/ vaccination marked on card - 1  No/don’t know – 0, reported by mother/ vaccination date on card/ vaccination marked on card - 1  No/don’t know – 0, reported by mother/ vaccination date on card/ vaccination marked on card - 1 |
| **Covariate factors**  **Individual level**  Sex of child  Birth order  Skilled birth assistance (SBA)  Birth quarter  Mother’s antenatal care (ANC) visits during pregnancy  Mother received tetanus toxoid (TT) injections before birth  Post-natal care (PNC) of baby within 2 months after birth  **Household level**  Maternal age  Maternal marital status  Maternal employment status (worked in the last 12 months)  Maternal occupation  Mother had problem seeking medical advice or treatment (Any of getting permission, getting money needed for treatment, distance – no nearby health facility, not wanting to go alone)  Maternal education  Maternal religion  Mother’s media exposure  Mother’s use of mobile phone or internet  Mother’s land ownership  Mother’s knowledge of malaria  Household owned a mosquito bednet  Mother had health insurance  Mother’s/household’s ethnicity  Sex of head of household  Household wealth index  Mother owned a bank account  Household size  Length of stay in place of residence | Male – 0, female – 1  (1^st^ and 2^nd^ births) 1 - 2 – 0, 3-5 – 1, > 5 – 2  No skilled attendant at birth – 0, skilled attendant at birth (yes) – 1  January – March – 0, April – June – 1, July – September – 2, October – December – 3  No ANC/don’t know – 0, 1 - 3 – 1, 4 or more – 2  Received no injection/don’t know – 0, 1 – 2 (injections) – 1, 3 – 4 – 2, 5 or more – 3  No/don’t know – 0, yes - 1  15-19 years – 0, 20-29 – 1, 30-39 – 2, 40-49 – 3  Never in union – 0, married/ living with partner – 1, divorced/widowed/ no longer living together/separated – 2  No – 0, currently working/ have a job, but on leave last 7 days/worked in the past year – 1  Agricultural – 0, clerical/sales/services – 1, professional/technical/managerial – 2, skilled manual/unskilled manual/other – 3 (categories varied slightly between countries)  Had problem seeking medical advice or treatment – 0, did not have problem seeking medical advice or treatment – 1  No education – 0, primary – 1, secondary – 2, higher – 2  Categories created based on major religions practised in each country  No – 0, yes (listens to radio or tv or reads newspaper at least once in a week) – 1  No (never used phone or internet) – 0, yes (used phone or internet) – 1 (for some countries, data were available on mobile phone usage only)  Does not own land – 0, alone/jointly/both alone and jointly – 1  No – 0, yes (agreed that malaria can lead to death or that malaria can be cured) – 1  No – 0, yes – 1  No – 0, yes – 1  Categories dependent on major ethnic groups within each country  Female – 0, male – 1  Poorest/poorer – 0, middle – 1, Richer/richest – 2  No – 0, yes – 1  >= 9 (large) – 0, 5 – 8 (medium) – 1, <= 4 (small) – 2  < 1 year/visitor – 0, 1- 3 years – 1, 4-5 years – 2, > 5 years/always – 3 |
| **Community/cluster level**  Urban/rural residence  Region  Travel time to the nearest city of at least 50,000 people (remoteness)  Conflict area  Urban slum | Rural – 0, urban – 1  Administrative level one regions in each country, grouped together in some cases (see Fig E)  Higher – 0, medium – 1, lower – 2 (classes determined using the tertiles of the travel time distribution in each country)  Yes – 0, no – 1  Yes – 0, no – 1 |

**Table B: Classification of travel time to cities (in minutes) using the tertiles of the extracted cluster-level data**

| **Country** | **Travel time class** | | |
| --- | --- | --- | --- |
|  | **Lower** | **Medium** | **Higher** |
| Nigeria | 0 - 9.86 | 9.87 - 32.1 | 32.2 - 592 |
| DRC | 0 - 45.2 | 45.3 - 174 | 174.1 - 1401.6 |
| Ethiopia | 0 - 40 | 40.1 - 122 | 122.1 - 673 |
| India | 0 - 10.4 | 10.5 - 28 | 28.1 - 1486.6 |
| Cambodia | 0 - 49.2 | 49.3 - 98.4 | 98.5 - 731 |
| Madagascar | 0 - 119 | 119.1 - 303 | 303.1 - 1340.8 |
| Mozambique | 0 - 10.1 | 10.2 - 71.9 | 72.0 - 396 |
| Pakistan | 0 - 5.13 | 5.14 - 35.2 | 35.3 - 827 |
| Zambia | 0 - 27.6 | 27.7 - 106 | 106.1 - 756 |

**Details of slum classification using DHS data**

Relevant information needed for the classification was obtained from each DHS survey. These were household characteristics outlined in Table C. We first selected clusters that were designated as urban and households within these in each survey. Following the UN-Habitat definition, the households were classified as slum or non-slum dwellings. Subsequently, a cluster was classified as a slum area if it had at least 10 households and if at least 75% of its households were classified as slum dwellings.

To classify households as slum dwellings, we implemented the following steps:

- For each household identified, assess whether or not it meets the following slum characteristics and produce a 0/1 binary score for each characteristic:
  - Access to improved water - a household is without access to safe water if it does not have access to piped water, borehole, protected well or spring or rainwater.
  - Access to improved sanitation - a household is without access to improved sanitation if (i) they rely on open defecation or use an unimproved pit latrine (or bucket toilet, hanging toilet etc), or (ii) they share a toilet with more than one other household.
  - Durability of housing - if the floor material of a house is made of earth, dung, sand or wood, its structure is considered inadequate or non-durable.
  - Overcrowding - a household is defined as overcrowded if there are more than three people per habitable/sleeping room.
- Classify the household as a slum dwelling if it meets at least 2 of the above criteria (or had a score ≥ 2).

As is often the case in previous studies [1, 2], due to lack of data, the last criterion in the UN Habitat definition (security of tenure) was not included in our classification.

DHS cluster locations classified as slum areas are mapped in Fig D for all nine countries.

**Table C: DHS variables used for slum classification**

| **Criteria** | **Relevant DHS variables** |
| --- | --- |
| Access to improved water | v201 – source of drinking water |
| Access to improved sanitation | v205 – type of toilet facility  v225 – shares toilet with other households  v238 – number of households sharing toilet |
| Durability of housing | hv213 – main floor material |
| Overcrowding | hv216 – number of rooms used for sleeping  hv009 – number of household members |

**
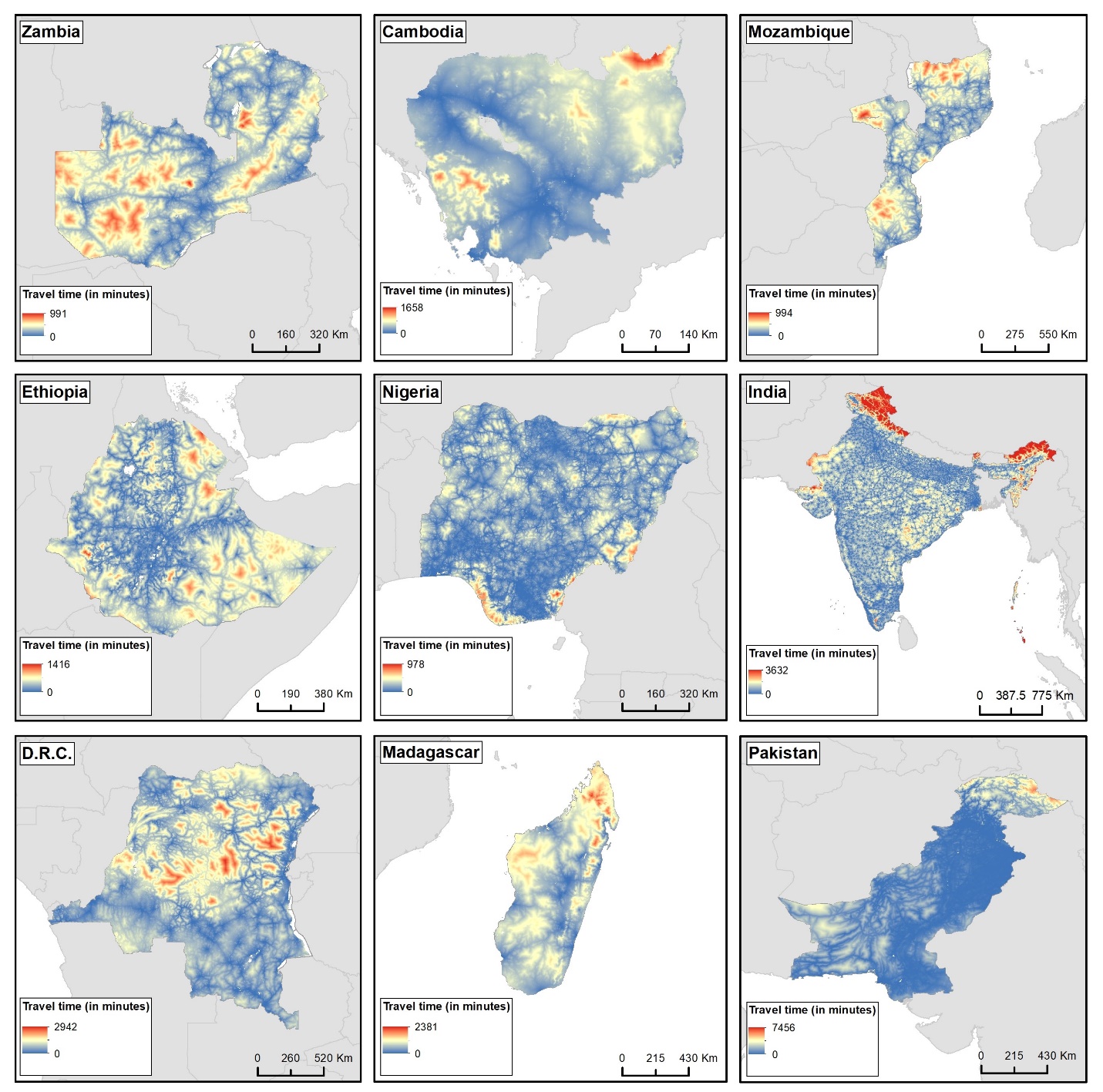
**

**Fig A: 1x1 km maps of estimated travel time to the nearest city of at least 50, 000 people, taken from Weiss et al [3]. The shapefiles for creating these maps were obtained from the global database of Global Administrative areas (GADM) (**[**https://gadm.org/data.html**](https://gadm.org/data.html)**). The maps were created using ESRI ArcGIS v10.6.**

**
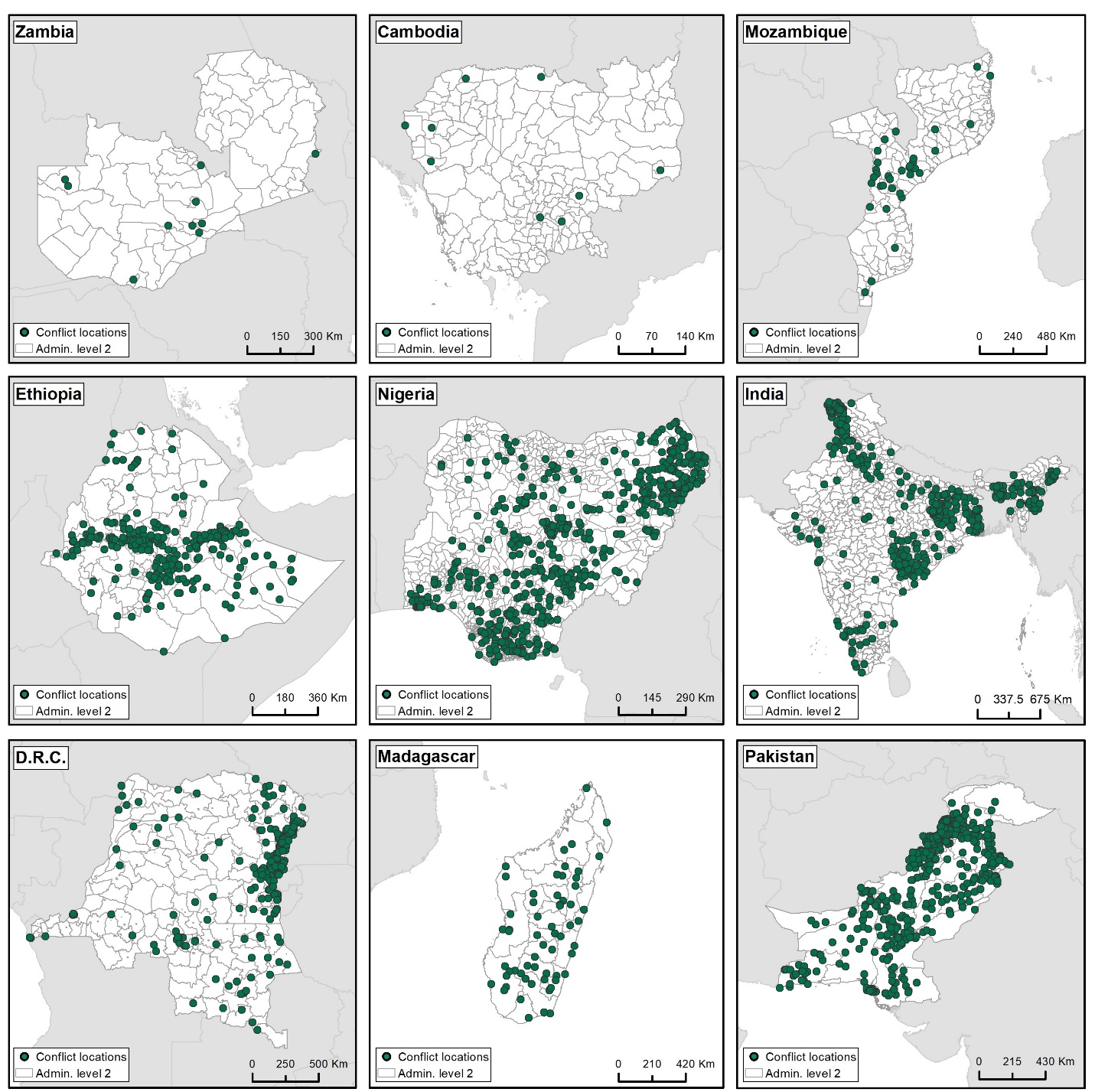
**

**Fig B: Locations of conflicts that resulted in at least one fatality within the 2 years prior to each DHS survey. Data were obtained from the Armed Conflict Location & Event Data Project (ACLED)** [**https://acleddata.com/#/dashboard**](https://acleddata.com/#/dashboard) **where conflicts with violent deaths (battles, explosions/remote violence, and violence against citizens) are registered. The shapefiles for creating these maps were obtained from the global database of Global Administrative areas (GADM) (**[**https://gadm.org/data.html**](https://gadm.org/data.html)**). The maps were created using ESRI ArcGIS v10.6.**

**
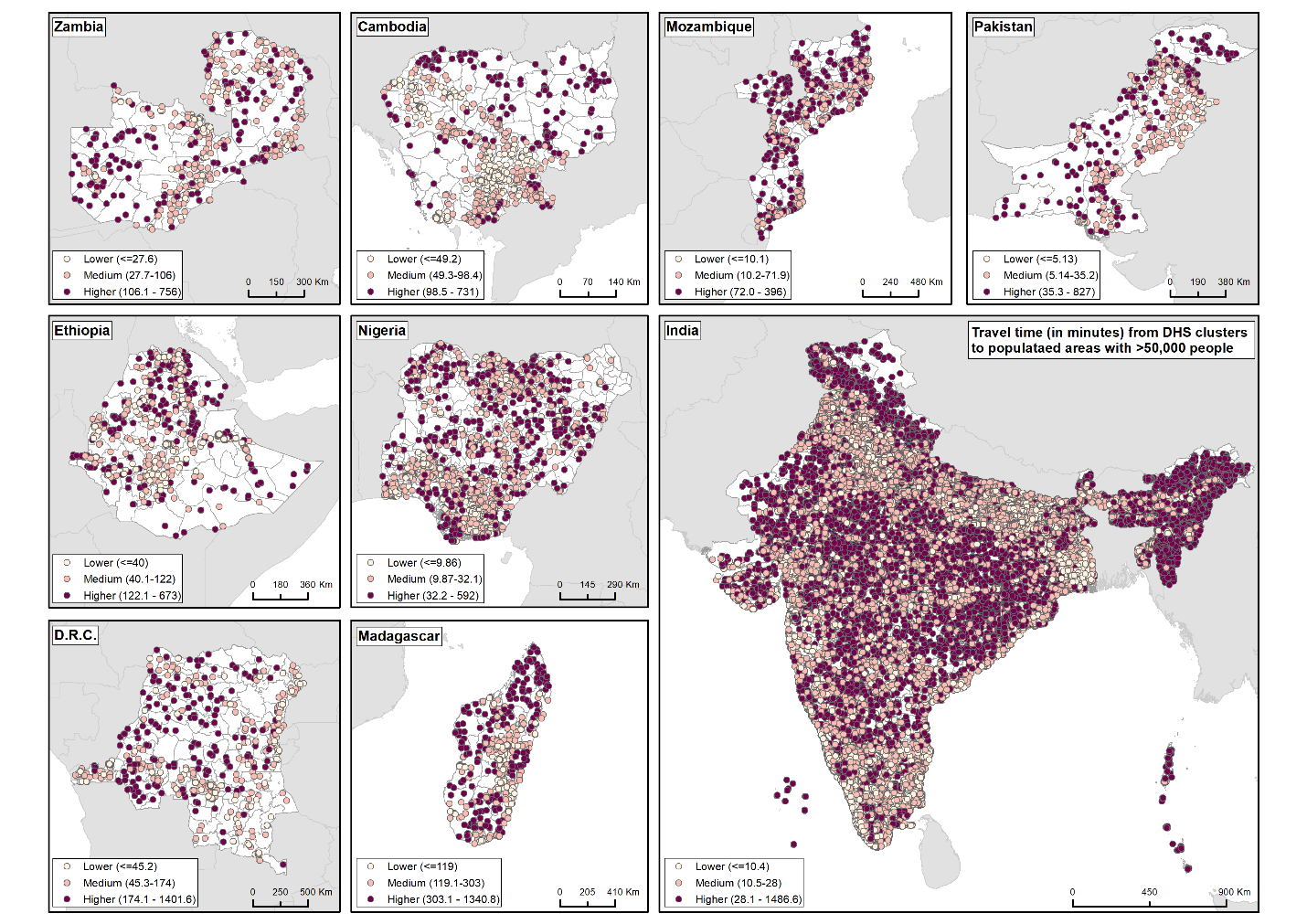
**

**Fig C: Maps of cluster/community level travel time to cities for all nine study countries. The information used in creating the classes is also provided in Table B. Conflict data were obtained from the Armed Conflict Location & Event Data Project (ACLED)** [**https://acleddata.com/#/dashboard**](https://acleddata.com/#/dashboard)**. The shapefiles for creating these maps were obtained from the global database of Global Administrative areas (GADM) (**[**https://gadm.org/data.html**](https://gadm.org/data.html)**). The maps were created using ESRI ArcGIS v10.6.**

**
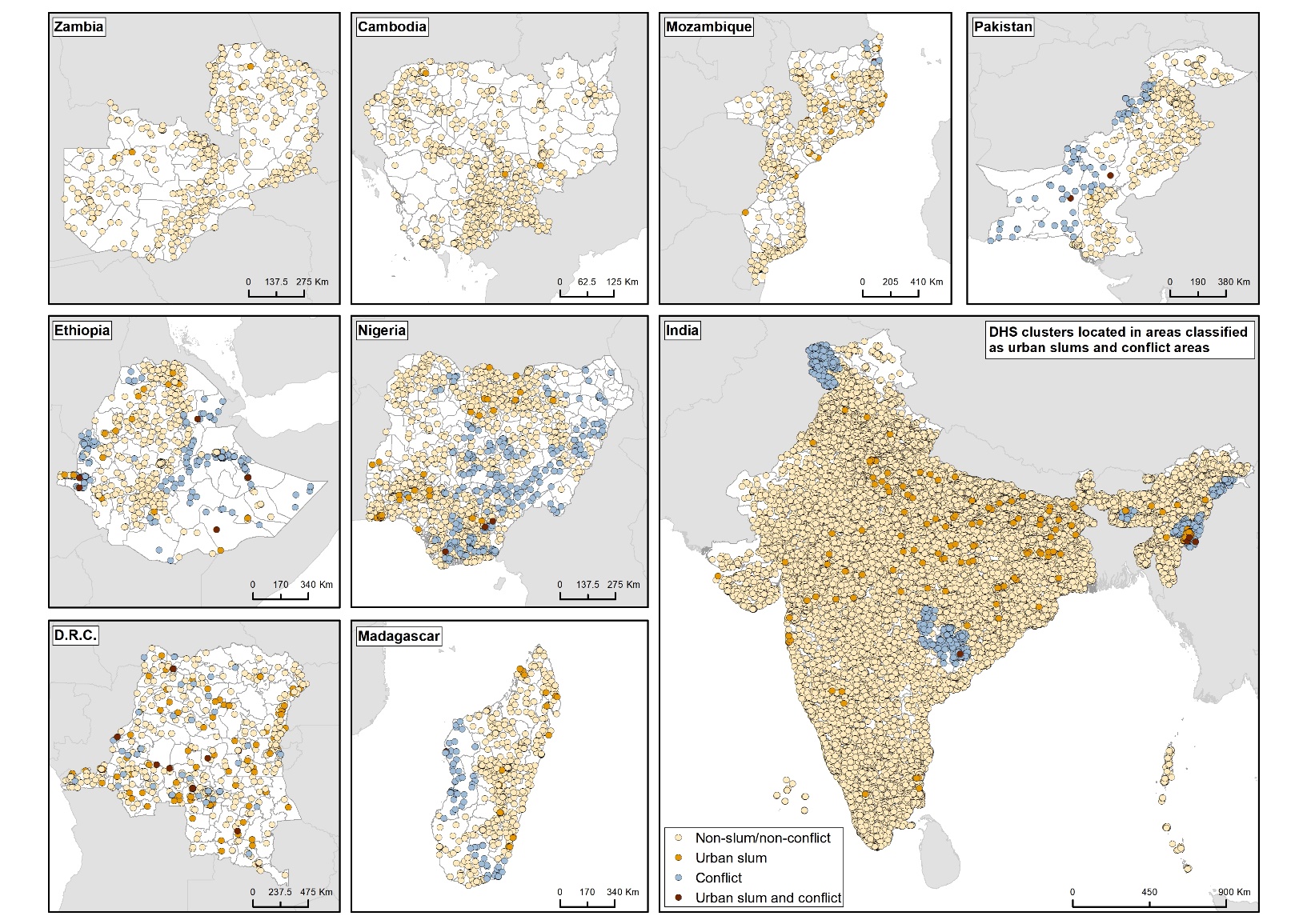
**

**Fig D: Classification of DHS cluster locations as slum and/or conflict areas in the study countries. The shapefiles for creating these maps were obtained from the global database of Global Administrative areas (GADM) (**[**https://gadm.org/data.html**](https://gadm.org/data.html)**). DHS data were obtained from the DHS program [4]. The maps were created using ESRI ArcGIS v10.6.**

**Table D: Number of DHS clusters found in conflict-affected areas and urban slums, and the total number of clusters processed for each country. The numbers of clusters in conflict-affected areas are based on the ‘narrow’ (> 300 deaths per 1 million pop.) and ‘broad’ (> 30 deaths per 1 million pop.) conflict definitions.**

| **Country** | **Number of clusters** | | | |
| --- | --- | --- | --- | --- |
|  | **Conflict**  **(‘narrow’ definition)** | **Conflict**  **(‘broad’ definition)** | **Urban slums** | **Total processed for analysis** |
| Cambodia | 0 | 0 | 4 | 611 |
| DRC | 8 | 106 | 62 | 492 |
| Ethiopia | 0 | 203 | 21 | 622 |
| India | 180 | 1,088 | 168 | 28,395 |
| Madagascar | 0 | 68 | 24 | 585 |
| Mozambique | 5 | 5 | 39 | 609 |
| Nigeria | 65 | 299 | 34 | 1,382 |
| Pakistan | 0 | 86 | 5 | 560 |
| Zambia | 0 | 0 | 12 | 535 |


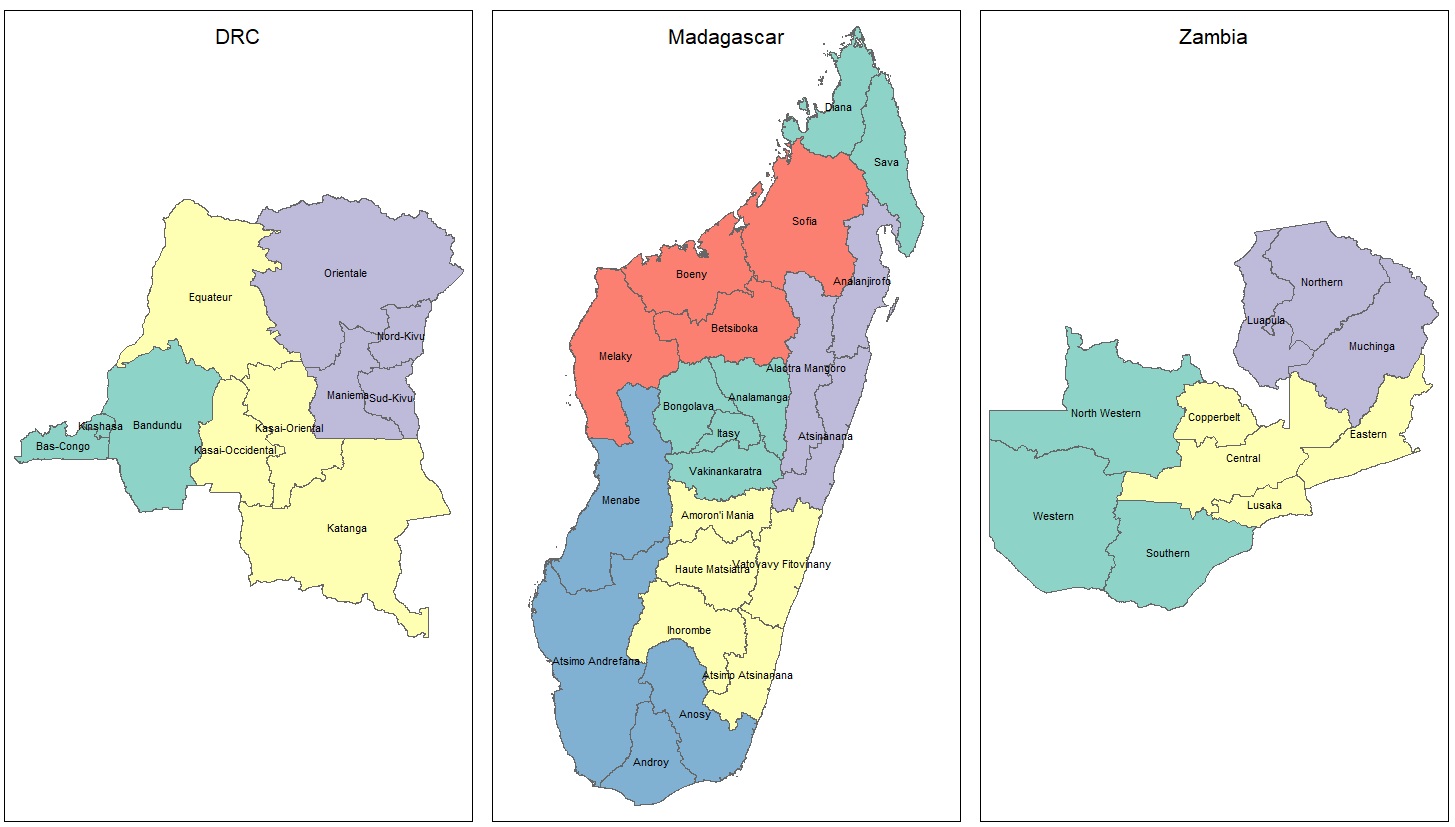


**Fig E: Maps of the regions included in the analyses for DRC, Madagascar and Zambia. Regions shown in the same colour were put into the same category in the analyses – see bivariate analyses tables and plots from the multivariate analyses. For Madagascar, the categories are based on the former six provinces, with two of these (Antananarivo and Antsiranana) combined into one category. The reference categories are shown in light green. The shapefiles for creating these maps were obtained from the global database of Global Administrative areas (GADM) (**[**https://gadm.org/data.html**](https://gadm.org/data.html)**). The maps were created using R programming language [5].**

**Analysis section**

**Test for (Multi)collinearity in multivariate analysis**

In the multivariate analyses, we addressed the problem of (multi)collinearity by computing the generalized variance inflation factors (GVIFs) [6] of the covariates for each country-vaccine combination and excluded variables that had high GVIFs ( > 2, on the scale that ensures comparability across the covariates as recommended by Fox and Monette [6]) or variables whose estimated relationships with the outcome variables were significant and not consistent between the bivariate and multivariate analyses (except in a few cases, e.g. important community factors), which can often occur due to undetected (multi)collinearity.

**Multi-level model for multivariate analysis**

The multi-level random intercept logistic regression model used in the multivariate analysis is described as follows. Let $y_{ijkl}$ denote the binary response (or vaccination status – DTP1, DTP3 or MCV1) for the $i$th child in household $j$, community/cluster $k$ and stratum $l$, and $p_{ijkl}$ - the corresponding probability of vaccination. The multi-level model is given by

$y_{ijkl}\sim\mathrm{Binomial}\left( 1, p_{ijkl} \right), i=1,\ldots,n_{jkl}, j=1,\ldots,n_{kl}, k=1,\ldots, n_{l}, l=1,\ldots, L$,

$\mathrm{logit}\left( p_{ijkl} \right)=\beta_{0}+\sum_{p=1}^{r_{1}} \beta_{p}^{ind}x_{pijkl}+\sum_{p=1}^{r_{2}} \beta_{p}^{house}x_{pjkl}+\sum_{p=1}^{r_{3}} \beta_{p}^{com}x_{pkl}+\delta_{jkl}^{house}+\delta_{kl}^{com}+ \delta_{l}^{strat}$,

$\delta_{jkl}^{house}\sim N\left( 0, \sigma_{house}^{2} \right), \delta_{kl}^{com}\sim N\left( 0, \sigma_{com}^{2} \right), \delta_{l}^{strat}\sim N\left( 0, \sigma_{strat}^{2} \right),$ (1)

where $r_{1},r_{2}$ and $r_{3}$ are the numbers of individual, household and community level covariates, respectively; $\beta_{0}$ is the overall intercept and $\beta_{p}^{ind}$, $\beta_{p}^{house}$ and $\beta_{p}^{com}$ are regression coefficients/fixed effects corresponding to the covariates $x_{pijkl}$, $x_{pjkl}$ and $x_{pkl}$ respectively; $\delta_{jkl}^{house}$, $\delta_{kl}^{com}$ and $\delta_{l}^{strat}$ are the household, community and stratification random effects with variances $\sigma_{house}^{2}$, $\sigma_{com}^{2}$ and $\sigma_{strat}^{2}$ respectively. The inclusion of clustering and stratification as random effects in the model serves to account for the complex design used in DHS surveys [7]. This is an alternative approach to including survey weights directly in the model. We note that no interaction terms were included in the model.

**Model estimation and evaluation**

All bivariate analyses were conducted in a frequentist framework. A fully Bayesian approach was used for estimating model (1). We placed a non-informative prior $N(0, {10}^{-3})$ on all regression coefficients and an informative $\mathrm{Gamma}(0.1, 0.1)$ prior with a mean of 1 and variance 10, on the precisions of the random effects to ensure that these were well-estimated, particularly $\sigma_{house}^{-2}$ whose estimation can often be affected by small sample sizes at this level [8]. We did not observe any meaningful differences in the estimation of the fixed effects with varying prior specifications on the variance parameters in model (1). Also, whether or not the household level was included in the model did not result in any meaningful changes in the estimates of the fixed effects.

We evaluated the contribution of the key community level variables (travel time/remoteness, conflict and urban slum) to explaining the variation in the outcome variables by computing the percentage change in total residual variation resulting from the inclusion of these variables in the model as: $PCV=100\times(\hat{\sigma}_{Tot1}^{2}-\hat{\sigma}_{Tot2}^{2})/\hat{\sigma}_{Tot1}^{2}$, where $\hat{\sigma}_{Tot1}^{2}=\hat{\sigma}_{house}^{2}+\hat{\sigma}_{com}^{2}+\hat{\sigma}_{strat}^{2}+\frac{\pi^{2}}{3}$ is the total residual variation when the variables were excluded from the model while $\hat{\sigma}_{Tot2}^{2}$ is the corresponding value when these were included. $\frac{\pi^{2}}{3}$ is a fixed individual level variance derived from the assumption that the observed binary responses arose from an underlying continuous variable having a logistic distribution with mean 0 and variance $\frac{\pi^{2}}{3}\cong3.29$ [9]. In the first scenario, we computed $\hat{\sigma}_{Tot1}^{2}$ from a no-covariate model and $\hat{\sigma}_{Tot2}^{2}$ from a model including the key community level variables only. In the second case, both estimates were computed from a model including all other covariates apart from the key community variables and a model including all the covariates, respectively. Positive $PCV$ values indicate an improvement in model fit due to the inclusion of these variables. Further, for each outcome variable, we assessed the proportion of the total residual variation (after accounting for covariate effects) lying at the various levels of the model’s hierarchy using the variance partitioning coefficient (VPC) [9]. For example, for the community level, the VPC is given by $\hat{\sigma}_{com}^{2}/(\hat{\sigma}_{house}^{2}+\hat{\sigma}_{com}^{2}+\hat{\sigma}_{strat}^{2}+\frac{\pi^{2}}{3})$. Finally, we evaluated the predictive or discriminatory ability of the fitted models by computing the area under the receiver operating characteristic curve (AUC) statistic, i.e. the area under the curve defined by plotting sensitivity against 1 minus specificity (sensitivity and specificity in our context relate to the proportions of vaccinated and unvaccinated children correctly classified by the fitted models). AUC scores close to 1 indicate good predictive power [10].

All analyses were carried out using ESRI ArcGIS v10.6, the R programming language [5], the R-INLA package [11] and Stata version 16 [12].

**Results section**

**Random effects – residual analysis and model evaluation**

In a regression analysis, as in model (1), covariates serve to explain the variation in the outcome variable. Often times, some residual variation could remain due to important unmeasured factors not included in the analysis. In our context, knowing what level of data where much of this residual variation lies is also key for understanding the heterogeneities that exist in the likelihood of vaccination.

Fig Fa shows that most of the residual variations in DTP1, DTP3 and MCV1 vaccinations were explained by the cluster/community level random effect. This is particularly the case for all three outcomes in DRC, Ethiopia, Madagascar, Mozambique, Pakistan and Zambia. However, there is also considerable variation at the stratum level, justifying the inclusion of this design variable in the fitted models. In nearly all cases, the least variation in vaccination occurred at the household level, as expected. The occurrence of higher residual variation in the odds of vaccination at the community level is indicative of the need to identify additional predictors of vaccination at this level. Also, it likely emphasizes the value of spatially-detailed estimates of vaccination coverage [13-15], and the need for targeting interventions at resolved spatial scales.

When examining the contribution of the key community variables to explaining the variation in the odds of vaccination, Fig Fb shows that in comparison with a no-covariate model, the model including only these variables accounted for reasonable amounts (up to 14.3%) of the total residual variation, most notably in Madagascar, Pakistan, Nigeria (DTP1 and DTP3) and Ethiopia (DTP3), all of which had PCV values that were > 5%. However, when all other variables have already been accounted for (Fig Fc), the inclusion of the key community variables only led to meaningful reductions in residual variation in Pakistan and DRC (DTP3). These findings suggest that whilst the key community variables are important for explaining the odds of vaccination, the combined effect of other covariates included in the analyses can be more influential.

Furthermore, the AUC scores plotted in Fig G range between 0.84 and 0.94 for all the country-vaccine combinations, showing that all the fitted models had good discriminatory power. India consistently had the lowest AUC scores while the highest scores were obtained for Mozambique and Pakistan - DTP1, Zambia - DTP3 and Pakistan - MCV1. In general, the fitted models had slightly higher discriminatory power for DTP1 and DTP3 compared to MCV1, suggesting that the receipt of these routine doses administered earlier in life is better explained by the factors included in the analyses.


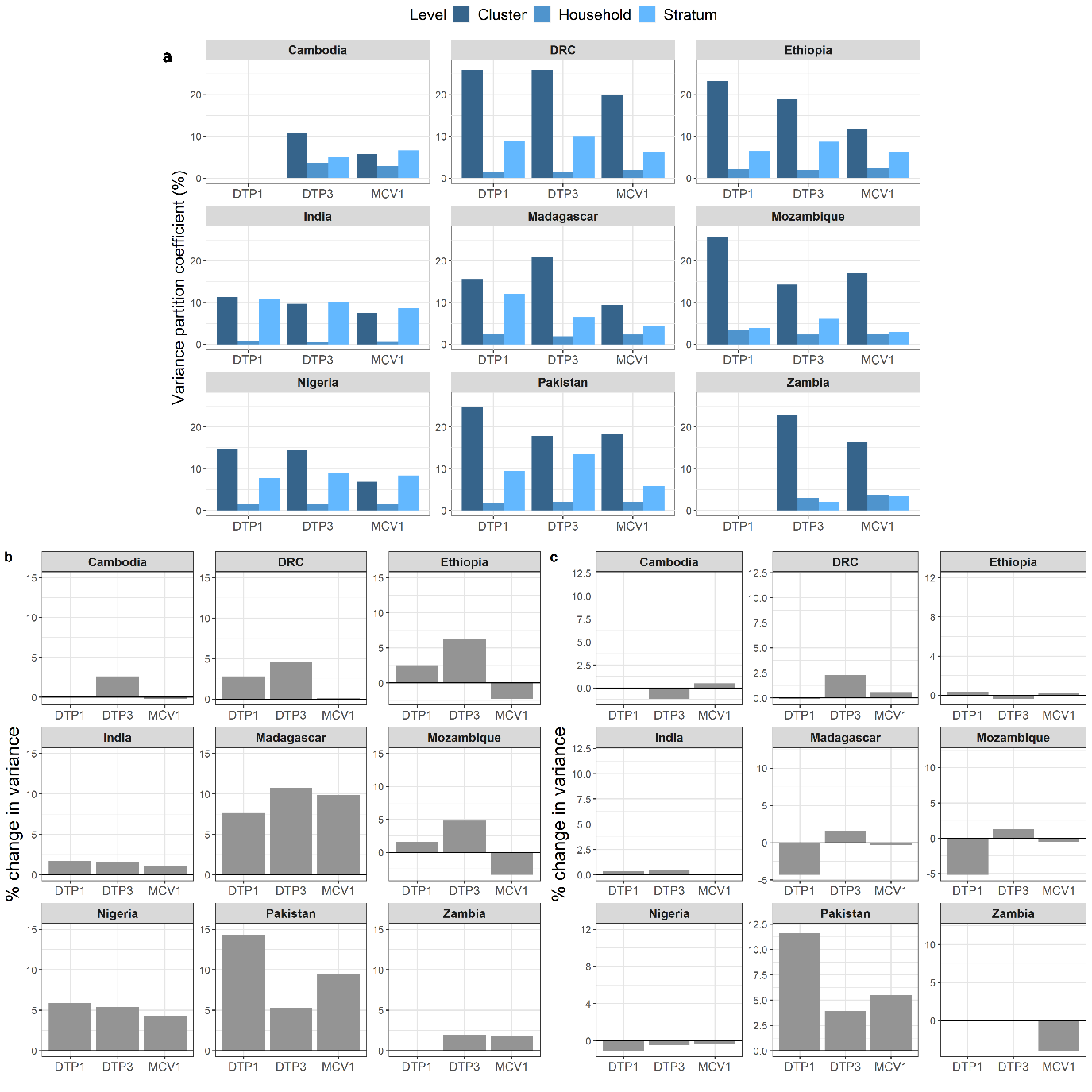


**Fig F: (a) Variance partition coefficient plots showing the proportion of variation in vaccination attributable to the different levels of data accounted for in the fitted models. (b) Percentage change in total residual variation when comparing a model including the key community variables (travel time, urban slum and conflict) only with a no-covariate (or empty) multi-level model. Positive values indicate a reduction in residual variation when the community variables were included in the analysis. (c) Percentage change in total residual variation when comparing the full model (including all the covariates) with a reduced model including all other covariates except the key community variables. Positive values indicate a reduction in residual variation when these variables were included in the analysis.**

**
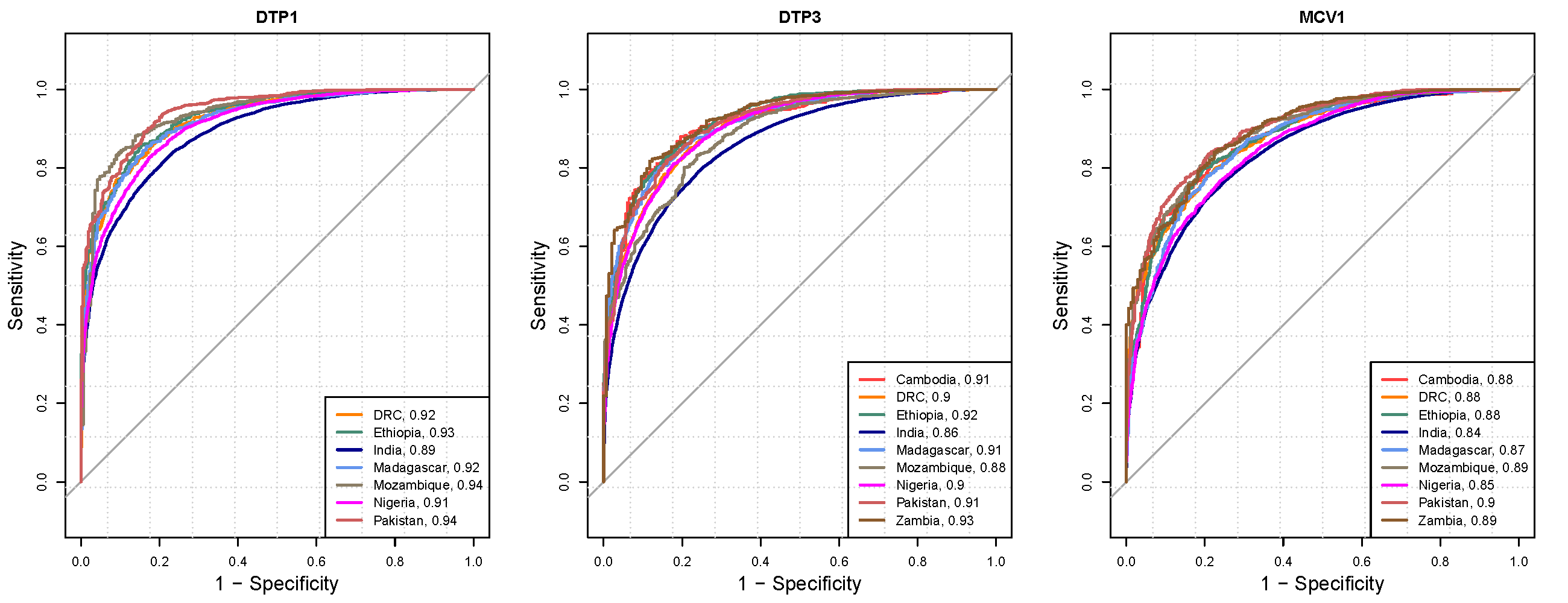
**

**Fig G: Plots of the area under the receiver operating characteristic curve (AUC) scores of the fitted models. The computed AUC scores are included in the legends for each country-vaccine combination.**

**Bivariate analysis results - tables**

**Table E: Bivariate analysis of factors associated with receipt of DPT3 and MCV1 for Cambodia, 2014 DHS**

|  |  | **DTP3** | | **MCV1** | |
| --- | --- | --- | --- | --- | --- |
| **Characteristics and categories** | **Number (%) in category** | **Percentage vaccinated**  **(95% CI)** | **cOR [95% CI]** | **Percentage vaccinated**  **(95% CI)** | **cOR [95% CI]** |
| Total number of children | 1377 |  |  |  |  |
| **Sex of child** |  |  |  |  |  |
| Male | 702 (51.0) | 84.5 [81.6-87.0] | 1.00 [reference] | 80.6 [77.5-83.4] | 1.00 [reference] |
| Female | 675 (49.0) | 84.4 [81.5-87.0] | 1.00 [0.75, 1.34] | 80.2 [77.0-83.0] | 0.97 [0.74, 1.27] |
| **Birth order** |  |  |  |  |  |
| 1-2 | 960 (69.7) | 86.7 [84.4-88.7] | 1.00 [reference] | 81.6 [79.0-83.9] | 1.00 [reference] |
| 3-5 | 374 (27.2) | 81.3 [77.0-84.9] | 0.67 [0.49, 0.92] * | 79.9 [75.6-83.7] | 0.90 [0.67, 1.22] |
| >5 | 43 (3.1) | 62.8 [47.6-75.8] | 0.26 [0.14, 0.50] * | 58.1 [43.1-71.8] | 0.31 [0.17, 0.59] * |
| **Skilled birth attendance (SBA)** |  |  |  |  |  |
| No | 136 (9.9) | 58.1 [49.6-66.1] | 1.00 [reference] | 55.9 [47.4-64.0] | 1.00 [reference] |
| Yes | 1241 (90.1) | 87.3 [85.4-89.1] | 4.98 [3.41, 7.28] * | 83.1 [80.9-85.1] | 3.88 [2.68, 5.61] * |
| **Mother’s ANC visits** |  |  |  |  |  |
| >=4 | 1048 (76.1) | 90.2 [88.2-91.8] | 1.00 [reference] | 85.3 [83.0-87.3] | 1.00 [reference] |
| 1-3 | 265 (19.2) | 70.6 [64.8-75.7] | 0.26 [0.19, 0.36] * | 68.3 [62.5-73.6] | 0.37 [0.27, 0.51] * |
| No ANC | 64 (4.6) | 48.4 [36.5-60.5] | 0.10 [0.06, 0.17] * | 50.0 [38.0-62.0] | 0.17 [0.10, 0.29] * |
| **Birth quarter** |  |  |  |  |  |
| Jan-Mar | 341 (24.8) | 87.7 [83.7-90.8] | 1.00 [reference] | 83.9 [79.6-87.4] | 1.00 [reference] |
| Apr-Jun | 333 (24.2) | 82.6 [78.1-86.3] | 0.67 [0.43, 1.02] | 74.8 [69.8-79.2] | 0.57 [0.39, 0.83] * |
| Jul-Sep | 303 (22.0) | 80.5 [75.7-84.6] | 0.58 [0.38, 0.89] * | 80.2 [75.3-84.3] | 0.78 [0.52, 1.17] |
| Oct-Dec | 400 (29.0) | 86.3 [82.5-89.3] | 0.88 [0.57, 1.36] | 82.3 [78.2-85.7] | 0.89 [0.61, 1.31] |
| **Mother’s TT vaccinations before birth** |  |  |  |  |  |
| 0 | 245 (17.8) | 77.6 [71.9-82.3] | 1.00 [reference] | 75.1 [69.3-80.1] | 1.00 [reference] |
| 1-2 | 799 (58.0) | 83.7 [81.0-86.1] | 1.49 [1.05, 2.12] * | 79.3 [76.4-82.0] | 1.27 [0.91, 1.78] |
| >=3 | 333 (24.2) | 91.3 [87.7-93.9] | 3.03 [1.87, 4.93] * | 86.8 [82.7-90.0] | 2.18 [1.42, 3.35] * |
| **Sex of head of household** |  |  |  |  |  |
| Female | 305 (22.1) | 85.2 [80.8-88.8] | 1.00 [reference] | 81.6 [76.9-85.6] | 1.00 [reference] |
| Male | 1072 (77.9) | 84.2 [81.9-86.3] | 0.92 [0.65, 1.32] | 80.0 [77.5-82.3] | 0.90 [0.65, 1.25] |
| **Maternal age (years)** |  |  |  |  |  |
| 15-19 | 65 (4.7) | 83.1 [72.0-90.4] | 1.00 [reference] | 70.8 [58.6-80.5] | 1.00 [reference] |
| 20-29 | 832 (60.4) | 84.7 [82.1-87.0] | 1.13 [0.58, 2.22] | 81.5 [78.7-84.0] | 1.82 [1.04, 3.19] * |
| 30-39 | 431 (31.3) | 85.4 [81.7-88.4] | 1.19 [0.59, 2.40] | 80.3 [76.2-83.8] | 1.68 [0.94, 3.02] |
| 40-49 | 49 (3.6) | 73.5 [59.5-83.9] | 0.56 [0.23, 1.40] | 75.5 [61.6-85.5] | 1.27 [0.55, 2.96] |
| **Maternal marital status** |  |  |  |  |  |
| Married/with partner | 1313 (95.4) | 84.8 [82.8-86.7] | 1.00 [reference] | 80.5 [78.3-82.6] | 1.00 [reference] |
| Divorced/widowed/separated | 64 (4.6) | 76.6 [64.7-85.4] | 0.58 [0.32, 1.06] | 78.1 [66.4-86.6] | 0.86 [0.47, 1.59] |
| **Mother had problem seeking medical advice or treatment** |  |  |  |  |  |
| Had problem | 373 (27.1) | 89.5 [86.0-92.3] | 1.00 [reference] | 85.5 [81.6-88.7] | 1.00 [reference] |
| No problem | 1004 (72.9) | 82.6 [80.1-84.8] | 0.55 [0.38, 0.80] * | 78.5 [75.8-80.9] | 0.62 [0.45, 0.85] * |
| **Maternal education** |  |  |  |  |  |
| None | 183 (13.3) | 68.3 [61.2-74.6] | 1.00 [reference] | 67.8 [60.6-74.1] | 1.00 [reference] |
| Primary | 651 (47.3) | 81.4 [78.2-84.2] | 2.03 [1.41, 2.94] * | 76.7 [73.2-79.7] | 1.56 [1.09, 2.24] * |
| Sec./higher | 543 (39.4) | 93.6 [91.2-95.3] | 6.73 [4.24, 10.70] * | 89.1 [86.2-91.5] | 3.90 [2.59, 5.89] * |
| **Maternal religion** |  |  |  |  |  |
| Buddhist | 1307 (94.9) | 85.8 [83.8-87.6] | 1.00 [reference] | 81.7 [79.5-83.7] | 1.00 [reference] |
| Christian/others | 41 (3.0) | 53.7 [38.5-68.2] | 0.19 [0.10, 0.36] * | 51.2 [36.3-66.0] | 0.23 [0.13, 0.44] * |
| Muslim | 29 (2.1) | 65.5 [46.9-80.3] | 0.31 [0.14, 0.68] * | 62.1 [43.6-77.6] | 0.37 [0.17, 0.79] * |
| **Mother’s media exposure** |  |  |  |  |  |
| No | 546 (39.7) | 76.9 [73.2-80.3] | 1.00 [reference] | 73.8 [70.0-77.3] | 1.00 [reference] |
| Yes | 831 (60.3) | 89.4 [87.1-91.3] | 2.53 [1.88, 3.41] * | 84.7 [82.1-87.0] | 1.97 [1.50, 2.57] * |
| **Mother’s use of mobile phone or internet** |  |  |  |  |  |
| No | 160 (11.6) | 73.8 [66.4-80.0] | 1.00 [reference] | 71.3 [63.8-77.7] | 1.00 [reference] |
| Yes | 1217 (88.4) | 85.9 [83.8-87.7] | 2.16 [1.47, 3.19] * | 81.6 [79.3-83.7] | 1.79 [1.23, 2.59] * |
| **Mother’s land ownership** |  |  |  |  |  |
| Does not own land | 562 (40.8) | 85.8 [82.6-88.4] | 1.00 [reference] | 82.6 [79.2-85.5] | 1.00 [reference] |
| Alone or jointly or both | 815 (59.2) | 83.6 [80.9-85.9] | 0.84 [0.62, 1.14] | 78.9 [76.0-81.6] | 0.79 [0.60, 1.04] |
| **Maternal employment status** |  |  |  |  |  |
| No | 326 (23.7) | 87.4 [83.4-90.6] | 1.00 [reference] | 84.7 [80.3-88.2] | 1.00 [reference] |
| Yes (currently/in the past 1 year) | 1051 (76.3) | 83.5 [81.2-85.7] | 0.73 [0.51, 1.05] | 79.1 [76.5-81.4] | 0.68 [0.49, 0.96] * |
| **Mother had health insurance** |  |  |  |  |  |
| No | 1158 (84.1) | 84.8 [82.6-86.8] | 1.00 [reference] | 80.6 [78.2-82.7] | 1.00 [reference] |
| Yes | 219 (15.9) | 82.6 [77.0-87.1] | 0.85 [0.58, 1.25] | 79.5 [73.6-84.3] | 0.93 [0.65, 1.33] |
| **Household wealth** |  |  |  |  |  |
| Poorer/poorest | 596 (43.3) | 75.0 [71.4-78.3] | 1.00 [reference] | 69.3 [65.5-72.9] | 1.00 [reference] |
| Middle | 194 (14.1) | 84.5 [78.7-89.0] | 1.82 [1.18, 2.80] * | 80.9 [74.8-85.9] | 1.88 [1.26, 2.80] * |
| Richer/richest | 587 (42.6) | 94.0 [91.8-95.7] | 5.26 [3.56, 7.75] * | 91.5 [88.9-93.5] | 4.76 [3.39, 6.67] * |
| **Mother owned a bank account** |  |  |  |  |  |
| No | 1130 (82.1) | 81.9 [79.5-84.0] | 1.00 [reference] | 77.6 [75.1-79.9] | 1.00 [reference] |
| Yes | 247 (17.9) | 96.4 [93.1-98.1] | 5.86 [2.96, 11.60] * | 93.1 [89.2-95.7] | 3.90 [2.34, 6.51] * |
| **Household size** |  |  |  |  |  |
| Large (>=9) | 152 (11.0) | 83.6 [76.8-88.6] | 1.00 [reference] | 77.0 [69.6-83.0] | 1.00 [reference] |
| Medium (5-8) | 766 (55.6) | 85.5 [82.8-87.8] | 1.16 [0.72, 1.87] | 83.4 [80.6-85.9] | 1.51 [0.99, 2.30] |
| Small (<=4) | 459 (33.3) | 83.0 [79.3-86.2] | 0.96 [0.59, 1.57] | 76.5 [72.4-80.1] | 0.97 [0.63, 1.50] |
| **Urban/rural** |  |  |  |  |  |
| Rural | 988 (71.8) | 81.5 [78.9-83.8] | 1.00 [reference] | 76.9 [74.2-79.4] | 1.00 [reference] |
| Urban | 389 (28.2) | 92.0 [88.9-94.3] | 2.63 [1.76, 3.92] * | 89.2 [85.7-91.9] | 2.48 [1.74, 3.53] * |
| **Travel time (mins)** |  |  |  |  |  |
| Higher (98.5 – 731) | 460 (33.4) | 79.3 [75.4-82.8] | 1.00 [reference] | 76.5 [72.4-80.2] | 1.00 [reference] |
| Medium (49.3 - 98.4) | 461 (33.5) | 85.5 [81.9-88.4] | 1.53 [1.09, 2.16] * | 80.3 [76.4-83.6] | 1.25 [0.91, 1.71] |
| Lower (0 - 49.2) | 456 (33.1) | 88.6 [85.3-91.2] | 2.02 [1.40, 2.92] * | 84.4 [80.8-87.5] | 1.66 [1.19, 2.32] * |

*Estimated crude odds ratio significant

**Table F: Bivariate analysis of factors associated with receipt of DPT1, DTP3 and MCV1 for DRC, 2013-14 DHS**

|  |  | **DTP1** |  | **DTP3** |  | **MCV1** |  |
| --- | --- | --- | --- | --- | --- | --- | --- |
| **Characteristics and categories** | **Number (%) in category** | **Percentage vaccinated**  **(95% CI)** | **cOR [95% CI]** | **Percentage vaccinated**  **(95% CI)** | **cOR [95% CI]** | **Percentage vaccinated**  **(95% CI)** | **COR [95% CI]** |
| Total number of children | 2948 |  |  |  |  |  |  |
| **Sex of child** |  |  |  |  |  |  |  |
| Male | 1488 (50.5) | 77.4 [75.2-79.5] | 1.00 [reference] | 54.4 [51.8-56.9] | 1.00 [reference] | 69.0 [66.6-71.3] | 1.00 [reference] |
| Female | 1460 (49.5) | 78.2 [76.0-80.2] | 1.04 [0.88, 1.24] | 56.9 [54.4-59.4] | 1.11 [0.96, 1.28] | 68.5 [66.1-70.8] | 0.98 [0.84, 1.14] |
| **Birth order** |  |  |  |  |  |  |  |
| 1-2 | 1062 (36.0) | 78.3 [75.8-80.7] | 1.00 [reference] | 55.2 [52.2-58.1] | 1.00 [reference] | 70.4 [67.6-73.1] | 1.00 [reference] |
| 3-5 | 1139 (38.6) | 78.4 [75.9-80.7] | 1.00 [0.82, 1.23] | 55.6 [52.7-58.4] | 1.02 [0.86, 1.20] | 68.4 [65.6-71.0] | 0.91 [0.76, 1.09] |
| >5 | 747 (25.3) | 76.0 [72.8-79.0] | 0.88 [0.70, 1.10] | 56.4 [52.8-59.9] | 1.05 [0.87, 1.27] | 66.8 [63.3-70.1] | 0.84 [0.69, 1.03] |
| **Birth quarter** |  |  |  |  |  |  |  |
| Jan-Mar | 588 (19.9) | 76.0 [72.4-79.3] | 1.00 [reference] | 54.3 [50.2-58.2] | 1.00 [reference] | 69.4 [65.5-73.0] | 1.00 [reference] |
| Apr-Jun | 708 (24.0) | 78.7 [75.5-81.5] | 1.16 [0.90, 1.51] | 58.6 [54.9-62.2] | 1.19 [0.96, 1.49] | 71.9 [68.5-75.1] | 1.13 [0.89, 1.43] |
| Jul-Sep | 763 (25.9) | 79.7 [76.7-82.4] | 1.24 [0.96, 1.60] | 56.5 [52.9-60.0] | 1.09 [0.88, 1.36] | 70.4 [67.0-73.5] | 1.05 [0.83, 1.33] |
| Oct-Dec | 889 (30.2) | 76.6 [73.7-79.3] | 1.03 [0.81, 1.32] | 53.4 [50.1-56.7] | 0.97 [0.79, 1.19] | 64.3 [61.1-67.4] | 0.80 [0.64, 0.99] * |
| **Skilled birth attendance (SBA)** |  |  |  |  |  |  |  |
| No | 672 (22.8) | 50.0 [46.2-53.8] | 1.00 [reference] | 27.4 [24.1-30.9] | 1.00 [reference] | 44.3 [40.6-48.1] | 1.00 [reference] |
| Yes | 2276 (77.2) | 86.0 [84.5-87.4] | 6.13 [5.06, 7.43] * | 64.0 [62.0-65.9] | 4.71 [3.89, 5.69] * | 75.9 [74.1-77.6] | 3.96 [3.31, 4.74] * |
| **Antenatal care attendance (ANC)** |  |  |  |  |  |  |  |
| No ANC | 311 (10.5) | 36.3 [31.2-41.8] | 1.00 [reference] | 20.6 [16.4-25.4] | 1.00 [reference] | 32.2 [27.2-37.6] | 1.00 [reference] |
| 1-3 | 1256 (42.6) | 80.1 [77.8-82.2] | 7.05 [5.39, 9.23] * | 55.6 [52.8-58.3] | 4.83 [3.59, 6.49] * | 69.9 [67.3-72.4] | 4.90 [3.75, 6.40] * |
| >=4 | 1381 (46.8) | 85.0 [83.0-86.8] | 9.94 [7.55, 13.07] * | 63.6 [61.0-66.1] | 6.74 [5.01, 9.06] * | 75.9 [73.6-78.1] | 6.64 [5.08, 8.68] * |
| **Post-natal care (PNC)** |  |  |  |  |  |  |  |
| No/don't know | 2445 (82.9) | 75.6 [73.9-77.3] | 1.00 [reference] | 52.1 [50.2-54.1] | 1.00 [reference] | 66.4 [64.5-68.2] | 1.00 [reference] |
| Yes | 503 (17.1) | 88.3 [85.2-90.8] | 2.43 [1.82, 3.23] * | 72.6 [68.5-76.3] | 2.43 [1.96, 3.00] * | 80.1 [76.4-83.4] | 2.04 [1.61, 2.58] * |
| **Sex of head of household** |  |  |  |  |  |  |  |
| Female | 621 (21.1) | 79.4 [76.0-82.4] | 1.00 [reference] | 58.0 [54.0-61.8] | 1.00 [reference] | 71.5 [67.8-74.9] | 1.00 [reference] |
| Male | 2327 (78.9) | 77.4 [75.6-79.0] | 0.89 [0.71, 1.10] | 55.0 [53.0-57.0] | 0.89 [0.74, 1.06] | 68.0 [66.1-69.9] | 0.85 [0.70, 1.03] |
| **Maternal age (years)** |  |  |  |  |  |  |  |
| 15-19 | 239 (8.1) | 76.2 [70.3-81.1] | 1.00 [reference] | 52.7 [46.4-59.0] | 1.00 [reference] | 64.4 [58.2-70.3] | 1.00 [reference] |
| 20-29 | 1552 (52.6) | 77.6 [75.4-79.6] | 1.08 [0.79, 1.49] | 54.8 [52.3-57.2] | 1.09 [0.83, 1.43] | 68.9 [66.5-71.1] | 1.22 [0.92, 1.63] |
| 30-39 | 951 (32.3) | 79.7 [77.0-82.1] | 1.23 [0.88, 1.72] | 57.6 [54.5-60.7] | 1.22 [0.92, 1.62] | 70.2 [67.3-73.1] | 1.30 [0.97, 1.76] |
| 40-49 | 206 (7.0) | 72.3 [65.8-78.0] | 0.82 [0.53, 1.25] | 56.3 [49.5-62.9] | 1.16 [0.79, 1.68] | 65.5 [58.8-71.7] | 1.05 [0.71, 1.55] |
| **Maternal marital status** |  |  |  |  |  |  |  |
| Never in union | 160 (5.4) | 85.0 [78.6-89.7] | 1.00 [reference] | 58.1 [50.3-65.5] | 1.00 [reference] | 78.8 [71.7-84.4] | 1.00 [reference] |
| Divorced/widowed/separated | 274 (9.3) | 77.4 [72.0-81.9] | 0.60 [0.36, 1.01] | 56.2 [50.3-62.0] | 0.92 [0.62, 1.37] | 70.1 [64.4-75.2] | 0.63 [0.40, 1.00] |
| Married/with partner | 2514 (85.3) | 77.4 [75.7-79.0] | 0.60 [0.39, 0.94] * | 55.4 [53.5-57.3] | 0.90 [0.65, 1.24] | 67.9 [66.1-69.7] | 0.57 [0.39, 0.84] * |
| **Mother had problem seeking medical advice or treatment** |  |  |  |  |  |  |  |
| Had problem | 531 (18.0) | 83.4 [80.0-86.4] | 1.00 [reference] | 65.2 [61.0-69.1] | 1.00 [reference] | 76.5 [72.7-79.9] | 1.00 [reference] |
| Had no problem | 2417 (82.0) | 76.5 [74.8-78.2] | 0.65 [0.51, 0.83] * | 53.5 [51.5-55.5] | 0.62 [0.51, 0.75] * | 67.0 [65.1-68.9] | 0.63 [0.50, 0.78] * |
| **Maternal education** |  |  |  |  |  |  |  |
| None | 581 (19.7) | 67.3 [63.4-71.0] | 1.00 [reference] | 48.4 [44.3-52.4] | 1.00 [reference] | 60.8 [56.7-64.7] | 1.00 [reference] |
| Primary | 1301 (44.1) | 73.4 [70.9-75.7] | 1.34 [1.08, 1.66] * | 48.4 [45.7-51.1] | 1.00 [0.82, 1.22] | 64.0 [61.4-66.6] | 1.15 [0.94, 1.41] |
| Sec./higher | 1066 (36.2) | 88.8 [86.8-90.6] | 3.87 [2.99, 5.00] * | 68.4 [65.5-71.1] | 2.31 [1.88, 2.84] * | 78.8 [76.2-81.2] | 2.40 [1.92, 3.00] * |
| **Mother’s religion** |  |  |  |  |  |  |  |
| Muslim | 60 (2.0) | 80.0 [68.0-88.3] | 1.00 [reference] | 51.7 [39.2-64.0] | 1.00 [reference] | 63.3 [50.5-74.5] | 1.00 [reference] |
| Christian | 2701 (91.6) | 78.4 [76.8-79.9] | 0.91 [0.48, 1.72] | 56.6 [54.7-58.4] | 1.22 [0.73, 2.03] | 69.5 [67.7-71.2] | 1.32 [0.77, 2.24] |
| Others | (6.3) | 67.9 [60.9-74.2] | 0.53 [0.26, 1.07] | 43.3 [36.4-50.5] | 0.71 [0.40, 1.28] | 59.9 [52.7-66.7] | 0.86 [0.47, 1.58] |
| **Mother’s media exposure** |  |  |  |  |  |  |  |
| No | 2221 (75.3) | 74.4 [72.5-76.2] | 1.00 [reference] | 50.7 [48.6-52.7] | 1.00 [reference] | 65.4 [63.4-67.4] | 1.00 [reference] |
| Yes | 727 (24.7) | 88.2 [85.6-90.3] | 2.57 [2.01, 3.28] * | 70.8 [67.4-74.0] | 2.37 [1.98, 2.83] * | 78.8 [75.7-81.6] | 1.97 [1.61, 2.40] * |
| **Mother’s use of mobile phone** |  |  |  |  |  |  |  |
| No | 1843 (62.5) | 71.0 [68.9-73.0] | 1.00 [reference] | 47.8 [45.5-50.1] | 1.00 [reference] | 63.8 [61.5-65.9] | 1.00 [reference] |
| Yes | 1105 (37.5) | 89.1 [87.2-90.8] | 3.36 [2.71, 4.16] * | 68.7 [65.9-71.4] | 2.40 [2.05, 2.80] * | 77.0 [74.4-79.4] | 1.90 [1.61, 2.26] * |
| **Mother’s land ownership** |  |  |  |  |  |  |  |
| Does not own land | 1654 (56.1) | 80.9 [78.9-82.7] | 1.00 [reference] | 58.0 [55.6-60.4] | 1.00 [reference] | 71.9 [69.7-74.0] | 1.00 [reference] |
| Alone or jointly or both | 1294 (43.9) | 73.8 [71.3-76.1] | 0.67 [0.56, 0.79] * | 52.6 [49.8-55.3] | 0.80 [0.69, 0.93]* | 64.7 [62.0-67.2] | 0.72 [0.61, 0.84]* |
| **Mother’s occupation** |  |  |  |  |  |  |  |
| (Un)skilled manual/army/other | 573 (19.4) | 80.1 [76.6-83.2] | 1.00 [reference] | 61.4 [57.4-65.3] | 1.00 [reference] | 70.3 [66.5-73.9] | 1.00 [reference] |
| Agric. | 1652 (56.0) | 74.5 [72.4-76.6] | 0.73 [0.58, 0.92] * | 50.8 [48.4-53.2] | 0.65 [0.53, 0.79] * | 66.2 [63.9-68.5] | 0.83 [0.67, 1.02] |
| Clerical/sales/services | 671 (22.8) | 82.6 [79.5-85.3] | 1.18 [0.88, 1.57] | 60.2 [56.5-63.8] | 0.95 [0.76, 1.19] | 71.5 [68.0-74.8] | 1.06 [0.83, 1.36] |
| Prof./tech./mgr. | 52 (1.8) | 94.2 [83.6-98.1] | 4.06 [1.24, 13.25] * | 86.5 [74.3-93.4] | 4.04 [1.79, 9.11] * | 94.2 [83.6-98.1] | 6.89 [2.12, 22.41] * |
| **Mother had health insurance** |  |  |  |  |  |  |  |
| No | 2861 (97.0) | 77.2 [75.6-78.7] | 1.00 [reference] | 54.7 [52.9-56.6] | 1.00 [reference] | 68.2 [66.5-69.9] | 1.00 [reference] |
| Yes | 87 (3.0) | 97.7 [91.3-99.4] | 12.57 [3.96, 76.48] * | 85.1 [75.9-91.1] | 4.71 [2.60, 8.53] * | 86.2 [77.3-92.0] | 2.92 [1.58, 5.39] * |
| **Household owned a mosquito bednet** |  |  |  |  |  |  |  |
| No | 766 (26.0) | 69.1 [65.7-72.2] | 1.00 [reference] | 48.0 [44.5-51.6] | 1.00 [reference] | 59.9 [56.4-63.3] | 1.00 [reference] |
| Yes | 2182 (74.0) | 80.8 [79.1-82.4] | 1.89 [1.57, 2.28] * | 58.3 [56.2-60.3] | 1.51 [1.28, 1.78] * | 71.8 [69.9-73.7] | 1.70 [1.43, 2.02] * |
| **Mother’s Ethnicity** |  |  |  |  |  |  |  |
| Bakongo Nord & Sud | 239 (8.1) | 94.6 [90.9-96.8] | 1.00 [reference] | 82.0 [76.6-86.4] | 1.00 [reference] | 86.2 [81.2-90.0] | 1.00 [reference] |
| Bas-kasai et Kwilu-Kwngo | 462 (15.7) | 87.0 [83.6-89.8] | 0.39 [0.21, 0.72] * | 66.7 [62.2-70.8] | 0.44 [0.30, 0.64] * | 76.6 [72.5-80.3] | 0.53 [0.34, 0.80] * |
| Basele-k , Man. et kivu | 532 (18.0) | 84.6 [81.3-87.4] | 0.32 [0.17, 0.58] * | 64.8 [60.7-68.8] | 0.40 [0.28, 0.59] * | 75.8 [71.9-79.2] | 0.50 [0.33, 0.76] * |
| Cuvette Central | 283 (9.6) | 64.3 [58.6-69.7] | 0.10 [0.06, 0.19] * | 35.3 [30.0-41.1] | 0.12 [0.08, 0.18] * | 59.7 [53.9-65.3] | 0.24 [0.15, 0.37] * |
| Kasai, Katanga, Tanganika | 825 (28.0) | 73.2 [70.1-76.1] | 0.16 [0.09, 0.28] * | 51.5 [48.1-54.9] | 0.23 [0.16, 0.33] * | 58.5 [55.1-61.9] | 0.23 [0.15, 0.34] * |
| Lunda, Pygmy, Foreign/Non-congolese, Others | 37 (1.3) | 86.5 [71.4-94.3] | 0.37 [0.12, 1.10] | 62.2 [45.8-76.2] | 0.36 [0.17, 0.76] * | 73.0 [56.6-84.8] | 0.43 [0.19, 0.98] * |
| Ubangi et Itimbiri | 360 (12.2) | 66.4 [61.3-71.1] | 0.11 [0.06, 0.21] * | 42.2 [37.2-47.4] | 0.16 [0.11, 0.24] * | 66.7 [61.6-71.3] | 0.32 [0.21, 0.49] * |
| Uele lac Albert | 210 (7.1) | 75.2 [69.0-80.6] | 0.17 [0.09, 0.33] * | 43.3 [36.8-50.1] | 0.17 [0.11, 0.26] * | 68.6 [62.0-74.5] | 0.35 [0.22, 0.56] * |
| **Household wealth** |  |  |  |  |  |  |  |
| Poorer/poorest | 1387 (47.0) | 67.8 [65.3-70.2] | 1.00 [reference] | 44.5 [41.9-47.1] | 1.00 [reference] | 61.1 [58.5-63.7] | 1.00 [reference] |
| Middle | 605 (20.5) | 79.3 [75.9-82.4] | 1.83 [1.46, 2.29] * | 54.0 [50.1-58.0] | 1.47 [1.21, 1.78] * | 69.8 [66.0-73.3] | 1.47 [1.19, 1.80] * |
| Richer/richest | 956 (32.4) | 91.3 [89.4-92.9] | 5.00 [3.89, 6.43] * | 72.8 [69.9-75.5] | 3.34 [2.80, 3.99] * | 79.1 [76.4-81.5] | 2.40 [1.99, 2.90] * |
| **Mother owned a bank account** |  |  |  |  |  |  |  |
| No | 2844 (96.5) | 77.1 [75.5-78.6] | 1.00 [reference] | 54.6 [52.8-56.5] | 1.00 [reference] | 68.0 [66.3-69.7] | 1.00 [reference] |
| Yes | 104 (3.5) | 96.2 [90.2-98.5] | 7.42 [2.72, 20.24] * | 82.7 [74.2-88.8] | 3.97 [2.37, 6.63] * | 88.5 [80.8-93.3] | 3.61 [1.97, 6.62] * |
| **Household size** |  |  |  |  |  |  |  |
| Large (>=9) | 683 (23.2) | 80.7 [77.5-83.5] | 1.00 [reference] | 58.7 [55.0-62.4] | 1.00 [reference] | 71.4 [67.9-74.7] | 1.00 [reference] |
| Medium (5-8) | 1552 (52.6) | 78.2 [76.0-80.1] | 0.86 [0.68, 1.07] | 56.4 [53.9-58.8] | 0.91 [0.76, 1.09] | 68.8 [66.4-71.0] | 0.88 [0.72, 1.07] |
| Small (<=4) | 713 (24.2) | 74.2 [70.9-77.3] | 0.69 [0.53, 0.89] * | 51.1 [47.4-54.7] | 0.73 [0.59, 0.91] * | 66.1 [62.5-69.4] | 0.78 [0.62, 0.98] * |
| **Urban/rural** |  |  |  |  |  |  |  |
| Rural | 2003 (67.9) | 72.1 [70.1-74.0] | 1.00 [reference] | 49.1 [46.9-51.3] | 1.00 [reference] | 64.7 [62.6-66.8] | 1.00 [reference] |
| Urban | 945 (32.1) | 89.8 [87.7-91.6] | 3.42 [2.71, 4.32] * | 69.4 [66.4-72.3] | 2.35 [2.00, 2.77] * | 77.2 [74.5-79.8] | 1.85 [1.55, 2.21] * |
| **Region** |  |  |  |  |  |  |  |
| Kinshasa, Bandundu, Bas-congo\| | 760 (25.8) | 90.8 [88.5-92.7] | 1.00 [reference] | 73.7 [70.4-76.7] | 1.00 [reference] | 81.6 [78.7-84.2] | 1.00 [reference] |
| Equateur, Kasai- occidental, Kasai-oriental, Katanga\| | 1376 (46.7) | 69.4 [66.9-71.8] | 0.23 [0.18, 0.30] * | 45.1 [42.4-47.7] | 0.29 [0.24, 0.36] * | 59.0 [56.4-61.6] | 0.33 [0.26, 0.40] * |
| Maniema, Nord-kivu, Orientale, Sud-kivu | 812 (27.5) | 79.8 [76.9-82.4] | 0.40 [0.30, 0.54] * | 56.7 [53.2-60.0] | 0.47 [0.38, 0.58] * | 73.2 [70.0-76.1] | 0.62 [0.48, 0.78] * |
| **Conflict area** |  |  |  |  |  |  |  |
| Yes | 657 (22.3) | 82.0 [78.9-84.8] | 1.00 [reference] | 62.7 [58.9-66.3] | 1.00 [reference] | 73.2 [69.7-76.5] | 1.00 [reference] |
| No | 2291 (77.7) | 76.6 [74.8-78.3] | 0.72 [0.57, 0.89] * | 53.6 [51.6-55.6] | 0.69 [0.57, 0.82] * | 67.4 [65.5-69.3] | 0.76 [0.62, 0.92] * |
| **Urban slum** |  |  |  |  |  |  |  |
| Yes | 418 (14.2) | 84.0 [80.1-87.2] | 1.00 [reference] | 57.9 [53.1-62.5] | 1.00 [reference] | 70.3 [65.8-74.5] | 1.00 [reference] |
| No | 2530 (85.8) | 76.8 [75.1-78.4] | 0.63 [0.48, 0.83] * | 55.3 [53.3-57.2] | 0.90 [0.73, 1.11] | 68.5 [66.6-70.2] | 0.92 [0.73, 1.15] |
| **Travel time (mins)** |  |  |  |  |  |  |  |
| Higher (174.1 - 1401.6) | 981 (33.3) | 69.9 [67.0-72.7] | 1.00 [reference] | 40.8 [37.7-43.9] | 1.00 [reference] | 65.2 [62.2-68.2] | 1.00 [reference] |
| Medium (45.3 – 174) | 987 (33.5) | 74.2 [71.3-76.8] | 1.23 [1.01, 1.50] * | 56.3 [53.2-59.4] | 1.87 [1.57, 2.24] * | 64.8 [61.8-67.8] | 0.98 [0.82, 1.18] |
| Lower (0 - 45.2) | 980 (33.2) | 89.3 [87.2-91.1] | 3.58 [2.81, 4.57] * | 69.8 [66.8-72.6] | 3.36 [2.79, 4.04] * | 76.1 [73.4-78.7] | 1.70 [1.39, 2.07] * |

*Estimated crude odds ratio significant

**Table G: Bivariate analysis of factors associated with receipt of DPT1, DTP3 and MCV1 for Ethiopia, 2016 DHS**

|  |  | **DTP1** |  | **DTP3** |  | **MCV1** |  |
| --- | --- | --- | --- | --- | --- | --- | --- |
| **Characteristics and categories** | **Number (%) in category** | **Percentage vaccinated**  **(95% CI)** | **cOR [95% CI]** | **Percentage vaccinated**  **(95% CI)** | **cOR [95% CI]** | **Percentage vaccinated**  **(95% CI)** | **cOR [95% CI]** |
| Total number of children | 1757 |  |  |  |  |  |  |
| **Sex of child** |  |  |  |  |  |  |  |
| Male | 862 (49.1) | 73.5 [70.5-76.4] | 1.00 [reference] | 55.1 [51.8-58.4] | 1.00 [reference] | 55.7 [52.3-59.0] | 1.00 [reference] |
| Female | 895 (50.9) | 74.1 [71.1-76.8] | 1.03 [0.83, 1.27] | 57.1 [53.8-60.3] | 1.08 [0.90, 1.31] | 59.6 [56.3-62.7] | 1.17 [0.97, 1.41] |
| **Birth order** |  |  |  |  |  |  |  |
| 1-2 | 681 (38.8) | 80.2 [77.0-83.0] | 1.00 [reference] | 65.2 [61.5-68.7] | 1.00 [reference] | 65.9 [62.3-69.4] | 1.00 [reference] |
| 3-5 | 630 (35.9) | 73.7 [70.1-76.9] | 0.69 [0.53, 0.90] * | 55.7 [51.8-59.6] | 0.67 [0.54, 0.84] * | 56.7 [52.8-60.5] | 0.68 [0.54, 0.84] * |
| >5 | 446 (25.4) | 64.3 [59.8-68.7] | 0.45 [0.34, 0.58] * | 42.8 [38.3-47.5] | 0.40 [0.31, 0.51] * | 46.4 [41.8-51.1] | 0.45 [0.35, 0.57] * |
| **Skilled birth attendance (SBA)** |  |  |  |  |  |  |  |
| No | 1041 (59.2) | 62.7 [59.7-65.6] | 1.00 [reference] | 42.0 [39.0-45.0] | 1.00 [reference] | 45.6 [42.6-48.7] | 1.00 [reference] |
| Yes | 716 (40.8) | 89.9 [87.5-91.9] | 5.31 [4.04, 6.99] * | 76.7 [73.4-79.6] | 4.54 [3.67, 5.62] * | 75.1 [71.8-78.2] | 3.60 [2.92, 4.44] * |
| **Antenatal care attendance (ANC)** |  |  |  |  |  |  |  |
| No ANC | 554 (31.5) | 46.6 [42.4-50.7] | 1.00 [reference] | 24.9 [21.5-28.7] | 1.00 [reference] | 30.9 [27.2-34.8] | 1.00 [reference] |
| 1-3 | 518 (29.5) | 79.3 [75.6-82.6] | 4.41 [3.36, 5.78] * | 60.0 [55.8-64.2] | 4.53 [3.49, 5.88] * | 59.1 [54.8-63.2] | 3.23 [2.51, 4.16] * |
| >=4 | 685 (39.0) | 91.7 [89.4-93.5] | 12.64 [9.19, 17.38] * | 78.4 [75.2-81.3] | 10.94 [8.39, 14.26] * | 78.2 [75.0-81.2] | 8.06 [6.24, 10.41] * |
| **Mother’s TT vaccinations before birth** |  |  |  |  |  |  |  |
| 0 | 775 (44.1) | 56.3 [52.7-59.7] | 1.00 [reference] | 39.1 [35.7-42.6] | 1.00 [reference] | 42.1 [38.6-45.6] | 1.00 [reference] |
| 1-2 | 570 (32.4) | 87.2 [84.2-89.7] | 5.29 [3.99, 7.03] * | 67.0 [63.0-70.8] | 3.17 [2.52, 3.97] * | 68.8 [64.8-72.4] | 3.03 [2.42, 3.81] * |
| 3-4 | 341 (19.4) | 88.6 [84.7-91.5] | 6.02 [4.19, 8.65] * | 73.3 [68.4-77.7] | 4.28 [3.23, 5.66] * | 71.8 [66.8-76.4] | 3.51 [2.67, 4.63] * |
| >=5 | 71 (4.0) | 87.3 [77.4-93.3] | 5.36 [2.62, 10.93] * | 71.8 [60.3-81.1] | 3.97 [2.32, 6.80] * | 70.4 [58.8-79.9] | 3.28 [1.93, 5.57] * |
| **Post-natal care (PNC)** |  |  |  |  |  |  |  |
| No/don't know | 1586 (90.3) | 71.8 [69.5-74.0] | 1.00 [reference] | 53.8 [51.4-56.3] | 1.00 [reference] | 55.2 [52.8-57.7] | 1.00 [reference] |
| Yes | 171 (9.7) | 92.4 [87.3-95.5] | 4.77 [2.68, 8.48] * | 77.2 [70.3-82.9] | 2.90 [2.00, 4.20] * | 80.1 [73.5-85.4] | 3.27 [2.21, 4.82] * |
| **Birth quarter** |  |  |  |  |  |  |  |
| Jan-Mar | 491 (27.9) | 73.9 [69.9-77.6] | 1.00 [reference] | 56.8 [52.4-61.1] | 1.00 [reference] | 58.9 [54.4-63.1] | 1.00 [reference] |
| Apr-Jun | 492 (28.0) | 72.6 [68.4-76.3] | 0.93 [0.70, 1.24] | 54.5 [50.0-58.8] | 0.91 [0.71, 1.17] | 52.8 [48.4-57.2] | 0.78 [0.61, 1.01] |
| Jul-Sep | 446 (25.4) | 73.5 [69.2-77.4] | 0.98 [0.73, 1.31] | 55.6 [51.0-60.2] | 0.95 [0.74, 1.23] | 59.9 [55.2-64.3] | 1.04 [0.80, 1.35] |
| Oct-Dec | 328 (18.7) | 75.9 [71.0-80.2] | 1.11 [0.80, 1.54] | 58.2 [52.8-63.5] | 1.06 [0.80, 1.41] | 60.1 [54.7-65.2] | 1.05 [0.79, 1.40] |
| **Mother’s employment status** |  |  |  |  |  |  |  |
| No | 994 (56.6) | 69.5 [66.6-72.3] | 1.00 [reference] | 50.0 [46.9-53.1] | 1.00 [reference] | 53.0 [49.9-56.1] | 1.00 [reference] |
| Yes (currently/in the past year) | 763 (43.4) | 79.4 [76.4-82.1] | 1.69 [1.36, 2.11] * | 64.1 [60.6-67.4] | 1.78 [1.47, 2.17] * | 63.7 [60.2-67.0] | 1.55 [1.28, 1.89] * |
| **Sex of head of household** |  |  |  |  |  |  |  |
| Female | 386 (22.0) | 67.4 [62.5-71.9] | 1.00 [reference] | 50.5 [45.5-55.5] | 1.00 [reference] | 54.1 [49.1-59.1] | 1.00 [reference] |
| Male | 1371 (78.0) | 75.6 [73.3-77.8] | 1.50 [1.18, 1.92] * | 57.7 [55.1-60.3] | 1.34 [1.07, 1.68] * | 58.6 [56.0-61.2] | 1.20 [0.96, 1.51] |
| **Maternal age (years)** |  |  |  |  |  |  |  |
| 15-19 | 90 (5.1) | 65.6 [55.2-74.6] | 1.00 [reference] | 47.8 [37.7-58.1] | 1.00 [reference] | 57.8 [47.4-67.5] | 1.00 [reference] |
| 20-29 | 905 (51.5) | 77.3 [74.5-80.0] | 1.79 [1.13, 2.85] * | 58.9 [55.7-62.1] | 1.57 [1.01, 2.42] * | 60.4 [57.2-63.6] | 1.12 [0.72, 1.73] |
| 30-39 | 638 (36.3) | 69.6 [65.9-73.0] | 1.20 [0.75, 1.92] | 53.3 [49.4-57.1] | 1.25 [0.80, 1.94] | 52.8 [48.9-56.7] | 0.82 [0.52, 1.28] |
| 40-49 | 124 (7.1) | 75.8 [67.5-82.5] | 1.65 [0.91, 2.99] | 56.5 [47.6-64.9] | 1.42 [0.82, 2.44] | 62.1 [53.3-70.2] | 1.20 [0.69, 2.08] |
| **Maternal marital status** |  |  |  |  |  |  |  |
| Never in union | 17 (1.0) | 88.2 [63.1-97.0] | 1.00 [reference] | 76.5 [51.4-90.9] | 1.00 [reference] | 76.5 [51.4-90.9] | 1.00 [reference] |
| Divorced/widowed/separated | 84 (4.8) | 69.0 [58.4-78.0] | 0.30 [0.06, 1.40] | 51.2 [40.6-61.7] | 0.32 [0.10, 1.07] | 60.7 [49.9-70.5] | 0.48 [0.14, 1.58] |
| Married/with partner | 1656 (94.3) | 73.9 [71.7-76.0] | 0.38 [0.09, 1.66] | 56.2 [53.8-58.5] | 0.39 [0.13, 1.21] | 57.3 [54.9-59.7] | 0.41 [0.13, 1.27] |
| **Mother had problem seeking medical advice or treatment** |  |  |  |  |  |  |  |
| Had problem | 520 (29.6) | 82.3 [78.8-85.4] | 1.00 [reference] | 67.7 [63.5-71.6] | 1.00 [reference] | 66.3 [62.2-70.3] | 1.00 [reference] |
| Had no problem | 1237 (70.4) | 70.3 [67.6-72.7] | 0.51 [0.39, 0.66] * | 51.3 [48.5-54.0] | 0.50 [0.40, 0.62] * | 54.0 [51.2-56.8] | 0.60 [0.48, 0.74] * |
| **Maternal education** |  |  |  |  |  |  |  |
| None | 1052 (59.9) | 64.6 [61.7-67.5] | 1.00 [reference] | 43.9 [40.9-46.9] | 1.00 [reference] | 47.7 [44.7-50.7] | 1.00 [reference] |
| Primary | 477 (27.1) | 86.2 [82.8-89.0] | 3.41 [2.55, 4.55] * | 69.4 [65.1-73.4] | 2.90 [2.30, 3.64] * | 67.3 [63.0-71.4] | 2.25 [1.80, 2.83] * |
| Sec./higher | 228 (13.0) | 90.4 [85.8-93.6] | 5.12 [3.24, 8.09] * | 84.6 [79.4-88.8] | 7.04 [4.82, 10.30] * | 83.3 [77.9-87.6] | 5.48 [3.79, 7.92] * |
| **Mother’s religion** |  |  |  |  |  |  |  |
| Islam | 807 (45.9) | 66.5 [63.2-69.7] | 1.00 [reference] | 45.6 [42.2-49.1] | 1.00 [reference] | 48.9 [45.5-52.4] | 1.00 [reference] |
| Christian | 910 (51.8) | 81.5 [78.9-83.9] | 2.22 [1.78, 2.77] * | 67.1 [64.0-70.1] | 2.44 [2.00, 2.96] * | 66.5 [63.3-69.5] | 2.07 [1.70, 2.51] * |
| Trad. / others | 40 (2.3) | 45.0 [30.5-60.4] | 0.41 [0.22, 0.78] * | 17.5 [8.6-32.4] | 0.25 [0.11, 0.58] * | 32.5 [19.9-48.3] | 0.50 [0.26, 0.99] * |
| **Mother’s media exposure** |  |  |  |  |  |  |  |
| No | 1366 (77.7) | 70.0 [67.5-72.4] | 1.00 [reference] | 50.4 [47.7-53.0] | 1.00 [reference] | 52.6 [49.9-55.2] | 1.00 [reference] |
| Yes | 391 (22.3) | 87.2 [83.5-90.2] | 2.92 [2.13, 4.02] * | 76.2 [71.7-80.2] | 3.16 [2.44, 4.08] * | 75.4 [70.9-79.5] | 2.77 [2.15, 3.57] * |
| **Mother’s use of phone or internet** |  |  |  |  |  |  |  |
| No | 1308 (74.4) | 69.1 [66.6-71.6] | 1.00 [reference] | 49.8 [47.1-52.5] | 1.00 [reference] | 52.0 [49.3-54.7] | 1.00 [reference] |
| Yes | 449 (25.6) | 87.5 [84.1-90.3] | 3.14 [2.32, 4.25] * | 74.6 [70.4-78.4] | 2.97 [2.34, 3.76] * | 74.2 [69.9-78.0] | 2.65 [2.09, 3.36] * |
| **Mother had health insurance** |  |  |  |  |  |  |  |
| No | 1692 (96.3) | 73.2 [71.1-75.3] | 1.00 [reference] | 55.0 [52.6-57.4] | 1.00 [reference] | 56.8 [54.4-59.1] | 1.00 [reference] |
| Yes | 65 (3.7) | 89.2 [79.1-94.8] | 3.03 [1.37, 6.69] * | 84.6 [73.7-91.5] | 4.50 [2.28, 8.88] * | 80.0 [68.5-88.0] | 3.04 [1.64, 5.63] * |
| **Mother’s ethnicity** |  |  |  |  |  |  |  |
| Amhara | 289 (16.4) | 33.8 [26.6-41.9] | 1.00 [reference] | 6.9 [3.7-12.3] | 1.00 [reference] | 18.6 [13.1-25.8] | 1.00 [reference] |
| Afar | 145 (8.3) | 76.0 [72.1-79.6] | 0.10 [0.06, 0.15] * | 60.4 [56.1-64.6] | 20.59 [10.57, 40.10] * | 59.6 [55.3-63.8] | 6.45 [4.09, 10.16] * |
| Oromo | 439 (25.0) | 84.1 [79.4-87.9] | 0.51 [0.35, 0.74] * | 72.0 [66.5-76.9] | 34.67 [17.36, 69.24] * | 71.3 [65.8-76.2] | 10.85 [6.65, 17.70] * |
| Tigrie | 224 (12.7) | 72.9 [68.5-76.8] | 1.93 [1.11, 3.37] * | 52.6 [47.9-57.3] | 14.99 [7.68, 29.27] * | 53.3 [48.6-57.9] | 4.99 [3.15, 7.89] * |
| Somalie | 155 (8.8) | 62.6 [54.7-69.8] | 0.32 [0.20, 0.50] * | 32.9 [26.0-40.7] | 6.62 [3.21, 13.66] * | 44.5 [36.9-52.4] | 3.51 [2.08, 5.92] * |
| Others | 505 (28.7) | 91.1 [86.6-94.2] | 0.60 [0.41, 0.87] * | 80.8 [75.1-85.4] | 56.83 [27.57, 117.13] * | 78.6 [72.7-83.5] | 16.02 [9.47, 27.12] * |
| **Household wealth** |  |  |  |  |  |  |  |
| Poorer/poorest | 876 (49.9) | 61.8 [58.5-64.9] | 1.00 [reference] | 39.8 [36.6-43.1] | 1.00 [reference] | 45.1 [41.8-48.4] | 1.00 [reference] |
| Middle | 259 (14.7) | 78.8 [73.4-83.3] | 2.30 [1.66, 3.19] * | 62.2 [56.1-67.9] | 2.48 [1.87, 3.30] * | 60.2 [54.1-66.0] | 1.84 [1.39, 2.45] * |
| Richer/richest | 622 (35.4) | 88.7 [86.0-91.0] | 4.88 [3.68, 6.48] * | 76.5 [73.0-79.7] | 4.92 [3.91, 6.19] * | 74.3 [70.7-77.6] | 3.52 [2.81, 4.40] * |
| **Household size** |  |  |  |  |  |  |  |
| Large (>=9) | 222 (12.6) | 63.1 [56.5-69.2] | 1.00 [reference] | 45.9 [39.5-52.5] | 1.00 [reference] | 46.4 [39.9-53.0] | 1.00 [reference] |
| Medium (5-8) | 958 (54.5) | 72.3 [69.4-75.1] | 1.53 [1.13, 2.08] * | 53.7 [50.5-56.8] | 1.36 [1.02, 1.83] * | 56.7 [53.5-59.8] | 1.51 [1.13, 2.03] * |
| Small (<=4) | 577 (32.8) | 80.4 [77.0-83.5] | 2.41 [1.71, 3.38] * | 64.1 [60.1-67.9] | 2.10 [1.54, 2.88] * | 63.6 [59.6-67.4] | 2.02 [1.48, 2.76] * |
| **Length of stay in place of residence** |  |  |  |  |  |  |  |
| <1 year/visitor | 22 (1.3) | 72.7 [51.0-87.2] | 1.00 [reference] | 59.1 [38.2-77.2] | 1.00 [reference] | 63.6 [42.3-80.7] | 1.00 [reference] |
| 1-3 year | 125 (7.1) | 87.2 [80.1-92.0] | 2.55 [0.87, 7.49] | 66.4 [57.7-74.1] | 1.37 [0.54, 3.46] | 66.4 [57.7-74.1] | 1.13 [0.44, 2.90] |
| 4-5 year | 84 (4.8) | 67.9 [57.2-76.9] | 0.79 [0.28, 2.25] | 53.6 [42.9-63.9] | 0.80 [0.31, 2.07] | 60.7 [49.9-70.5] | 0.88 [0.33, 2.34] |
| >5 year /always | 1526 (86.9) | 73.1 [70.8-75.2] | 1.02 [0.40, 2.62] | 55.4 [52.9-57.9] | 0.86 [0.37, 2.02] | 56.7 [54.2-59.2] | 0.75 [0.31, 1.79] |
| **Urban/rural** |  |  |  |  |  |  |  |
| Rural | 1374 (78.2) | 68.5 [66.0-70.9] | 1.00 [reference] | 49.2 [46.6-51.8] | 1.00 [reference] | 51.5 [48.8-54.1] | 1.00 [reference] |
| Urban | 383 (21.8) | 93.0 [89.9-95.1] | 6.07 [4.04, 9.12] * | 80.9 [76.7-84.6] | 4.38 [3.33, 5.78] * | 79.9 [75.6-83.6] | 3.75 [2.86, 4.92] * |
| **Conflict area** |  |  |  |  |  |  |  |
| Yes | 627 (35.7) | 70.8 [67.1-74.2] | 1.00 [reference] | 50.6 [46.6-54.5] | 1.00 [reference] | 53.4 [49.5-57.3] | 1.00 [reference] |
| No | 1130 (64.3) | 75.5 [72.9-77.9] | 1.27 [1.02, 1.58] * | 59.2 [56.3-62.0] | 1.42 [1.17, 1.73] * | 60.0 [57.1-62.8] | 1.31 [1.07, 1.59] * |
| **Urban slum** |  |  |  |  |  |  |  |
| Yes | 68 (3.9) | 79.4 [68.2-87.4] | 1.00 [reference] | 52.9 [41.1-64.4] | 1.00 [reference] | 55.9 [44.0-67.2] | 1.00 [reference] |
| No | 1689 (96.1) | 73.6 [71.4-75.6] | 0.72 [0.40, 1.31] | 56.2 [53.9-58.6] | 1.14 [0.70, 1.86] | 57.7 [55.4-60.1] | 1.08 [0.66, 1.76] |
| **Travel time (mins)** |  |  |  |  |  |  |  |
| Higher (122.1 – 673) | 580 (33.0) | 60.0 [56.0-63.9] | 1.00 [reference] | 38.4 [34.6-42.5] | 1.00 [reference] | 44.0 [40.0-48.0] | 1.00 [reference] |
| Medium (40.1 – 122) | 589 (33.5) | 76.7 [73.2-80.0] | 2.20 [1.71, 2.83] * | 55.9 [51.8-59.8] | 2.03 [1.60, 2.56] * | 60.3 [56.3-64.2] | 1.93 [1.53, 2.44] * |
| Lower (0 – 40) | 588 (33.5) | 84.5 [81.4-87.2] | 3.64 [2.76, 4.81] * | 73.8 [70.1-77.2] | 4.51 [3.52, 5.78] * | 68.5 [64.7-72.2] | 2.78 [2.19, 3.53] * |

*Estimated crude odds ratio significant

**Table H: Bivariate analysis of factors associated with receipt of DPT1, DTP3 and MCV1 for India, 2015-16 DHS**

|  |  | **DTP1** |  | **DTP3** |  | **MCV1** |  |
| --- | --- | --- | --- | --- | --- | --- | --- |
| **Characteristics and categories** | **Number (%) in category** | **Percentage vaccinated**  **(95% CI)** | **cOR [95% CI]** | **Percentage vaccinated**  **(95% CI)** | **cOR [95% CI]** | **Percentage vaccinated**  **(95% CI)** | **cOR [95% CI]** |
| Total number of children | 46130 |  |  |  |  |  |  |
| **Sex of child** |  |  |  |  |  |  |  |
| Male | 24167 (52.4) | 88.5 [88.1-88.9] | 1.00 [reference] | 77.2 [76.7-77.7] | 1.00 [reference] | 79.4 [78.9-79.9] | 1.00 [reference] |
| Female | 21963 (47.6) | 88.3 [87.9-88.8] | 0.98 [0.93, 1.04] | 77.4 [76.9-78.0] | 1.02 [0.97, 1.06] | 78.9 [78.4-79.5] | 0.97 [0.93, 1.01] |
| **Birth order** |  |  |  |  |  |  |  |
| 1-2 | 31432 (68.1) | 90.7 [90.4-91.0] | 1.00 [reference] | 80.6 [80.2-81.0] | 1.00 [reference] | 82.4 [82.0-82.9] | 1.00 [reference] |
| 3-5 | 12966 (28.1) | 85.1 [84.5-85.7] | 0.59 [0.55, 0.63] * | 71.8 [71.0-72.5] | 0.61 [0.58, 0.64] * | 73.9 [73.2-74.7] | 0.60 [0.57, 0.63] * |
| >5 | 1732 (3.8) | 72.7 [70.6-74.8] | 0.27 [0.25, 0.31] * | 59.0 [56.7-61.3] | 0.35 [0.31, 0.38] * | 59.5 [57.1-61.8] | 0.31 [0.28, 0.35] * |
| **Skilled birth attendance (SBA)** |  |  |  |  |  |  |  |
| No | 8731 (18.9) | 75.4 [74.4-76.3] | 1.00 [reference] | 59.8 [58.7-60.8] | 1.00 [reference] | 62.9 [61.9-63.9] | 1.00 [reference] |
| Yes | 37399 (81.1) | 91.5 [91.2-91.8] | 3.52 [3.31, 3.74] * | 81.4 [81.0-81.8] | 2.95 [2.81, 3.10] * | 83.0 [82.6-83.4] | 2.88 [2.73, 3.03] * |
| **Antenatal care attendance (ANC)** |  |  |  |  |  |  |  |
| No ANC | 8347 (18.1) | 73.2 [72.2-74.1] | 1.00 [reference] | 57.4 [56.3-58.4] | 1.00 [reference] | 60.6 [59.6-61.7] | 1.00 [reference] |
| 1-3 | 16232 (35.2) | 89.2 [88.7-89.6] | 3.02 [2.81, 3.23] * | 76.3 [75.7-77.0] | 2.40 [2.26, 2.54] * | 78.8 [78.1-79.4] | 2.41 [2.27, 2.55] * |
| >=4 | 21551 (46.7) | 93.8 [93.5-94.1] | 5.58 [5.18, 6.01] * | 85.8 [85.3-86.2] | 4.48 [4.23, 4.74] * | 86.7 [86.2-87.1] | 4.23 [3.99, 4.48] * |
| **Birth quarter** |  |  |  |  |  |  |  |
| Jan-Mar | 12016 (26.0) | 86.8 [86.1-87.4] | 1.00 [reference] | 74.8 [74.1-75.6] | 1.00 [reference] | 76.3 [75.5-77.1] | 1.00 [reference] |
| Apr-Jun | 10301 (22.3) | 88.4 [87.7-89.0] | 1.16 [1.07, 1.26] * | 77.6 [76.8-78.4] | 1.16 [1.09, 1.24] * | 78.8 [78.0-79.6] | 1.16 [1.09, 1.23] * |
| Jul-Sep | 11952 (25.9) | 89.2 [88.6-89.7] | 1.26 [1.16, 1.36] * | 78.3 [77.6-79.1] | 1.22 [1.14, 1.29] * | 81.1 [80.4-81.8] | 1.34 [1.25, 1.42] * |
| Oct-Dec | 11861 (25.7) | 89.5 [88.9-90.0] | 1.30 [1.20, 1.40] * | 78.5 [77.8-79.2] | 1.23 [1.16, 1.30] * | 80.5 [79.7-81.2] | 1.28 [1.20, 1.36] * |
| **Mother’s TT vaccinations before birth** |  |  |  |  |  |  |  |
| 0 | 4171 (9.0) | 60.5 [59.0-62.0] | 1.00 [reference] | 47.7 [46.2-49.2] | 1.00 [reference] | 50.2 [48.7-51.7] | 1.00 [reference] |
| 1-2 | 34895 (75.6) | 91.2 [90.9-91.5] | 6.79 [6.32, 7.30] * | 80.3 [79.9-80.7] | 4.47 [4.19, 4.78] * | 82.1 [81.7-82.5] | 4.55 [4.26, 4.87] * |
| >=3 | 7064 (15.3) | 91.2 [90.5-91.8] | 6.76 [6.10, 7.49] * | 79.9 [78.9-80.8] | 4.35 [4.00, 4.73] * | 81.9 [81.0-82.8] | 4.50 [4.13, 4.90] * |
| **Post-natal care** **(PNC)** |  |  |  |  |  |  |  |
| No/don't know | 29708 (64.4) | 85.9 [85.5-86.3] | 1.00 [reference] | 73.4 [72.9-73.9] | 1.00 [reference] | 75.7 [75.2-76.2] | 1.00 [reference] |
| Yes | 16422 (35.6) | 93.1 [92.7-93.5] | 2.23 [2.08, 2.39] * | 84.4 [83.9-85.0] | 1.97 [1.87, 2.07] * | 85.5 [84.9-86.0] | 1.89 [1.79, 1.98] * |
| **Sex of head of household** |  |  |  |  |  |  |  |
| Female | 5629 (12.2) | 89.7 [88.9-90.4] | 1.00 [reference] | 79.1 [78.0-80.2] | 1.00 [reference] | 80.0 [79.0-81.1] | 1.00 [reference] |
| Male | 40501 (87.8) | 88.3 [88.0-88.6] | 0.87 [0.79, 0.95] * | 77.1 [76.6-77.5] | 0.89 [0.83, 0.95] * | 79.1 [78.7-79.5] | 0.94 [0.88, 1.01] |
| **Maternal age (years)** |  |  |  |  |  |  |  |
| 15-19 | 1602 (3.5) | 85.9 [84.1-87.5] | 1.00 [reference] | 73.5 [71.3-75.6] | 1.00 [reference] | 76.0 [73.9-78.1] | 1.00 [reference] |
| 20-29 | 33614 (72.9) | 89.5 [89.1-89.8] | 1.40 [1.21, 1.61] * | 78.4 [78.0-78.9] | 1.31 [1.17, 1.47] * | 80.4 [80.0-80.8] | 1.29 [1.15, 1.46] * |
| 30-39 | 10130 (22.0) | 86.2 [85.5-86.9] | 1.03 [0.88, 1.20] | 74.9 [74.1-75.8] | 1.08 [0.96, 1.21] | 76.6 [75.8-77.5] | 1.03 [0.91, 1.17] |
| 40-49 | 784 (1.7) | 78.3 [75.3-81.1] | 0.59 [0.48, 0.74] * | 66.7 [63.3-69.9] | 0.72 [0.60, 0.87] * | 66.6 [63.2-69.8] | 0.63 [0.52, 0.76] * |
| **Maternal marital status** |  |  |  |  |  |  |  |
| Never married /divorced/ widowed/separated | 612 (1.3) | 84.3 [81.2-87.0] | 1.00 [reference] | 71.7 [68.0-75.2] | 1.00 [reference] | 73.2 [69.6-76.6] | 1.00 [reference] |
| Married/with partner | 45518 (98.7) | 88.5 [88.2-88.8] | 1.43 [1.15, 1.78] * | 77.4 [77.0-77.8] | 1.35 [1.13, 1.61] * | 79.3 [78.9-79.6] | 1.40 [1.17, 1.68] * |
| **Mother had problem seeking medical advice or treatment** |  |  |  |  |  |  |  |
| Had problem | 23250 (50.4) | 90.8 [90.5-91.2] | 1.00 [reference] | 80.6 [80.1-81.1] | 1.00 [reference] | 82.3 [81.8-82.8] | 1.00 [reference] |
| Had no problem | 22880 (49.6) | 86.0 [85.6-86.5] | 0.62 [0.58, 0.66] * | 74.0 [73.4-74.5] | 0.68 [0.66, 0.72] * | 76.0 [75.4-76.5] | 0.68 [0.65, 0.71] * |
| **Maternal education** |  |  |  |  |  |  |  |
| None | 13096 (28.4) | 81.7 [81.1-82.4] | 1.00 [reference] | 67.2 [66.4-68.0] | 1.00 [reference] | 69.9 [69.1-70.7] | 1.00 [reference] |
| Primary | 6531 (14.2) | 87.2 [86.4-88.0] | 1.53 [1.40, 1.66] * | 75.0 [73.9-76.0] | 1.46 [1.37, 1.56] * | 76.3 [75.3-77.3] | 1.39 [1.29, 1.48] * |
| Sec./higher | 26503 (57.5) | 92.1 [91.7-92.4] | 2.60 [2.44, 2.77] * | 82.9 [82.4-83.3] | 2.35 [2.24, 2.47] * | 84.5 [84.0-84.9] | 2.34 [2.23, 2.46] * |
| **Mother’s religion** |  |  |  |  |  |  |  |
| Hindu | 33169 (71.9) | 90.6 [90.3-90.9] | 1.00 [reference] | 79.8 [79.4-80.2] | 1.00 [reference] | 82.2 [81.8-82.6] | 1.00 [reference] |
| Budd | 440 (1.0) | 86.1 [82.6-89.1] | 0.64 [0.49, 0.84] * | 76.1 [71.9-79.9] | 0.81 [0.65, 1.01] | 78.9 [74.8-82.4] | 0.81 [0.64, 1.02] |
| Christian | 3771 (8.2) | 77.2 [75.9-78.6] | 0.35 [0.32, 0.38] * | 64.8 [63.3-66.3] | 0.47 [0.43, 0.50] * | 64.7 [63.1-66.2] | 0.40 [0.37, 0.43] * |
| Muslim | 7381 (16.0) | 83.5 [82.7-84.4] | 0.52 [0.49, 0.56] * | 70.8 [69.7-71.8] | 0.61 [0.58, 0.65] * | 71.8 [70.7-72.8] | 0.55 [0.52, 0.58] * |
| Others | 617 (1.3) | 88.3 [85.5-90.6] | 0.78 [0.61, 1.00] | 77.5 [74.0-80.6] | 0.87 [0.72, 1.05] | 76.7 [73.2-79.8] | 0.71 [0.59, 0.86] * |
| Sikh | 752 (1.6) | 98.3 [97.0-99.0] | 5.88 [3.39, 10.19] * | 94.9 [93.1-96.3] | 4.76 [3.43, 6.60] * | 94.4 [92.5-95.8] | 3.66 [2.68, 5.01] * |
| **Mother’s media exposure** |  |  |  |  |  |  |  |
| No | 16760 (36.3) | 83.1 [82.5-83.6] | 1.00 [reference] | 69.3 [68.6-70.0] | 1.00 [reference] | 71.5 [70.8-72.2] | 1.00 [reference] |
| Yes | 29370 (63.7) | 91.5 [91.2-91.8] | 2.20 [2.07, 2.33] * | 81.9 [81.5-82.3] | 2.01 [1.92, 2.10] * | 83.6 [83.2-84.0] | 2.03 [1.94, 2.13] * |
| **Mother had health insurance** |  |  |  |  |  |  |  |
| No | 39831 (86.3) | 87.8 [87.5-88.2] | 1.00 [reference] | 76.2 [75.8-76.6] | 1.00 [reference] | 78.3 [77.9-78.7] | 1.00 [reference] |
| Yes | 6299 (13.7) | 92.3 [91.6-92.9] | 1.65 [1.49, 1.82] * | 84.2 [83.2-85.1] | 1.66 [1.54, 1.78] * | 85.0 [84.1-85.9] | 1.58 [1.47, 1.70] * |
| **Household owned a mosquito bednet** |  |  |  |  |  |  |  |
| No | 26040 (56.4) | 88.7 [88.3-89.0] | 1.00 [reference] | 77.1 [76.6-77.6] | 1.00 [reference] | 80.1 [79.6-80.6] | 1.00 [reference] |
| Yes | 20090 (43.6) | 88.2 [87.7-88.6] | 0.95 [0.90, 1.01] | 77.6 [77.0-78.1] | 1.02 [0.98, 1.07] | 78.0 [77.4-78.6] | 0.88 [0.84, 0.92] * |
| **Mother’s ethnicity** |  |  |  |  |  |  |  |
| Tribe | 7540 (16.3) | 81.7 [80.8-82.6] | 1.00 [reference] | 68.6 [67.6-69.7] | 1.00 [reference] | 70.6 [69.6-71.6] | 1.00 [reference] |
| Caste/Don't know | 36759 (79.7) | 89.8 [89.5-90.1] | 1.97 [1.84, 2.11] * | 79.1 [78.6-79.5] | 1.73 [1.63, 1.82] * | 80.9 [80.5-81.3] | 1.77 [1.67, 1.87] * |
| No caste/tribe | 1831 (4.0) | 88.9 [87.3-90.2] | 1.78 [1.52, 2.09] * | 77.9 [76.0-79.8] | 1.61 [1.43, 1.82] * | 79.9 [78.0-81.7] | 1.66 [1.46, 1.88] * |
| **Household wealth** |  |  |  |  |  |  |  |
| Poorer/poorest | 22348 (48.4) | 84.7 [84.2-85.1] | 1.00 [reference] | 71.4 [70.8-72.0] | 1.00 [reference] | 73.5 [73.0-74.1] | 1.00 [reference] |
| Middle | 9348 (20.3) | 90.4 [89.8-91.0] | 1.72 [1.59, 1.86] * | 80.2 [79.4-81.0] | 1.63 [1.53, 1.72] * | 81.6 [80.8-82.4] | 1.59 [1.50, 1.69] * |
| Richer/richest | 14434 (31.3) | 93.0 [92.6-93.4] | 2.42 [2.25, 2.61] * | 84.6 [84.0-85.2] | 2.21 [2.09, 2.33] * | 86.4 [85.8-86.9] | 2.28 [2.16, 2.41] * |
| **Household size** |  |  |  |  |  |  |  |
| Large (>=9) | 8741 (18.9) | 87.1 [86.3-87.7] | 1.00 [reference] | 74.4 [73.5-75.3] | 1.00 [reference] | 77.2 [76.3-78.1] | 1.00 [reference] |
| Medium (5-8) | 25617 (55.5) | 88.6 [88.2-89.0] | 1.16 [1.07, 1.24] * | 77.6 [77.1-78.1] | 1.19 [1.12, 1.26] * | 79.6 [79.1-80.0] | 1.15 [1.08, 1.22] * |
| Small (<=4) | 11772 (25.5) | 89.1 [88.6-89.7] | 1.22 [1.12, 1.33] * | 78.8 [78.1-79.6] | 1.28 [1.20, 1.37] * | 79.9 [79.1-80.6] | 1.17 [1.09, 1.25] * |
| **Length of stay in place of residence** |  |  |  |  |  |  |  |
| <1 year/visitor | 3111 (6.7) | 88.7 [87.6-89.8] | 1.00 [reference] | 73.6 [72.0-75.1] | 1.00 [reference] | 80.8 [79.4-82.1] | 1.00 [reference] |
| 1-3 year | 12459 (27.0) | 90.6 [90.1-91.1] | 1.22 [1.08, 1.39] * | 81.3 [80.6-82.0] | 1.56 [1.43, 1.71] * | 82.3 [81.7-83.0] | 1.11 [1.00, 1.23] * |
| 4-5 year | 8676 (18.8) | 90.4 [89.8-91.0] | 1.20 [1.05, 1.37] * | 79.7 [78.8-80.5] | 1.41 [1.28, 1.55] * | 81.2 [80.3-82.0] | 1.03 [0.92, 1.14] |
| >5 year/always | 21884 (47.4) | 86.4 [85.9-86.8] | 0.80 [0.71, 0.90] * | 74.6 [74.0-75.2] | 1.06 [0.97, 1.15] | 76.4 [75.8-76.9] | 0.77 [0.70, 0.85] * |
| **Urban/rural** |  |  |  |  |  |  |  |
| Rural | 35005 (75.9) | 87.8 [87.5-88.2] | 1.00 [reference] | 76.4 [76.0-76.9] | 1.00 [reference] | 78.4 [77.9-78.8] | 1.00 [reference] |
| Urban | 11125 (24.1) | 90.4 [89.8-90.9] | 1.30 [1.21, 1.40] * | 80.0 [79.3-80.8] | 1.23 [1.17, 1.30] * | 81.8 [81.1-82.5] | 1.24 [1.17, 1.31] * |
| **Conflict area** |  |  |  |  |  |  |  |
| Yes | 1964 (4.3) | 89.4 [87.9-90.6] | 1.00 [reference] | 81.3 [79.5-83.0] | 1.00 [reference] | 79.1 [77.3-80.9] | 1.00 [reference] |
| No | 44166 (95.7) | 88.4 [88.1-88.7] | 0.91 [0.78, 1.05] | 77.1 [76.7-77.5] | 0.78 [0.69, 0.87] * | 79.2 [78.8-79.6] | 1.00 [0.90, 1.12] |
| **Urban slum** |  |  |  |  |  |  |  |
| Yes | 360 (0.8) | 86.7 [82.7-89.8] | 1.00 [reference] | 71.1 [66.2-75.6] | 1.00 [reference] | 74.4 [69.7-78.7] | 1.00 [reference] |
| No | 45770 (99.2) | 88.5 [88.2-88.8] | 1.18 [0.87, 1.60] | 77.4 [77.0-77.7] | 1.39 [1.10, 1.75] * | 79.2 [78.9-79.6] | 1.31 [1.03, 1.66] * |
| **Travel time (mins)** |  |  |  |  |  |  |  |
| Higher (28.1 - 1486.6) | 15383 (33.3) | 84.7 [84.2-85.3] | 1.00 [reference] | 72.1 [71.4-72.8] | 1.00 [reference] | 74.7 [74.0-75.4] | 1.00 [reference] |
| Medium (10.5 – 28) | 15383 (33.3) | 89.5 [89.0-90.0] | 1.54 [1.44, 1.65] * | 78.4 [77.8-79.1] | 1.40 [1.33, 1.48] * | 80.0 [79.4-80.6] | 1.35 [1.28, 1.43] * |
| Lower (0 - 10.4) | 15364 (33.3) | 91.1 [90.6-91.5] | 1.84 [1.72, 1.98] * | 81.4 [80.8-82.0] | 1.69 [1.60, 1.78] * | 82.8 [82.2-83.4] | 1.63 [1.54, 1.73] * |

*Estimated crude odds ratio significant

**Table I: Bivariate analysis of factors associated with receipt of DPT1, DTP3 and MCV1 for Madagascar, 2008-09 DHS**

|  |  | **DTP1** |  | **DTP3** |  | **MCV1** |  |
| --- | --- | --- | --- | --- | --- | --- | --- |
| **Characteristics and categories** | **Number (%) in category** | **Percentage vaccinated**  **(95% CI)** | **cOR [95% CI]** | **Percentage vaccinated**  **(95% CI)** | **cOR [95% CI]** | **Percentage vaccinated**  **(95% CI)** | **cOR [95% CI]** |
| Total number of children | 2039 |  |  |  |  |  |  |
| **Sex of child** |  |  |  |  |  |  |  |
| Male | 1006 (49.3) | 81.8 [79.3-84.1] | 1.00 [reference] | 70.9 [68.0-73.6] | 1.00 [reference] | 66.2 [63.2-69.1] | 1.00 [reference] |
| Female | 1033 (50.7) | 83.4 [81.0-85.5] | 1.11 [0.89, 1.40] | 70.9 [68.0-73.6] | 1.00 [0.83, 1.21] | 68.3 [65.4-71.1] | 1.10 [0.92, 1.33] |
| **Birth order** |  |  |  |  |  |  |  |
| 1-2 | 912 (44.7) | 85.7 [83.3-87.9] | 1.00 [reference] | 74.7 [71.7-77.4] | 1.00 [reference] | 72.9 [69.9-75.7] | 1.00 [reference] |
| 3-5 | 739 (36.2) | 80.0 [76.9-82.7] | 0.66 [0.51, 0.86] * | 68.1 [64.6-71.3] | 0.72 [0.58, 0.90] * | 65.4 [61.9-68.7] | 0.70 [0.57, 0.86] * |
| >5 | 388 (19.0) | 80.2 [75.9-83.8] | 0.67 [0.49, 0.92] * | 67.3 [62.4-71.8] | 0.70 [0.54, 0.90] * | 57.7 [52.8-62.6] | 0.51 [0.40, 0.65] * |
| **Skilled birth attendance (SBA)** |  |  |  |  |  |  |  |
| No | 1095 (53.7) | 74.2 [71.5-76.7] | 1.00 [reference] | 59.7 [56.8-62.6] | 1.00 [reference] | 54.9 [51.9-57.8] | 1.00 [reference] |
| Yes | 944 (46.3) | 92.4 [90.5-93.9] | 4.22 [3.20, 5.56] * | 83.8 [81.3-86.0] | 3.49 [2.82, 4.31] * | 81.7 [79.1-84.0] | 3.66 [2.99, 4.49] * |
| **Antenatal care attendance (ANC)** |  |  |  |  |  |  |  |
| No ANC | 218 (10.7) | 44.5 [38.0-51.2] | 1.00 [reference] | 30.7 [25.0-37.2] | 1.00 [reference] | 28.9 [23.3-35.3] | 1.00 [reference] |
| 1-3 | 786 (38.5) | 83.8 [81.1-86.3] | 6.47 [4.66, 8.98] * | 70.2 [66.9-73.3] | 5.32 [3.84, 7.36] * | 64.5 [61.1-67.8] | 4.47 [3.22, 6.20] * |
| >=4 | 1035 (50.8) | 89.7 [87.7-91.4] | 10.82 [7.75, 15.11] * | 79.8 [77.2-82.1] | 8.91 [6.43, 12.33] * | 77.5 [74.8-79.9] | 8.47 [6.11, 11.75] * |
| **Mother’s TT vaccinations before birth** |  |  |  |  |  |  |  |
| 0 | 685 (33.6) | 67.7 [64.1-71.1] | 1.00 [reference] | 55.2 [51.4-58.9] | 1.00 [reference] | 51.5 [47.8-55.3] | 1.00 [reference] |
| 1-2 | 992 (48.7) | 90.3 [88.3-92.0] | 4.45 [3.41, 5.79] * | 77.8 [75.1-80.3] | 2.85 [2.30, 3.52] * | 73.8 [71.0-76.4] | 2.65 [2.15, 3.25] * |
| 3-4 | 320 (15.7) | 90.3 [86.6-93.1] | 4.44 [2.97, 6.65] * | 82.5 [77.9-86.3] | 3.83 [2.77, 5.30] * | 80.6 [75.9-84.6] | 3.91 [2.86, 5.36] * |
| >=5 | 42 (2.1) | 83.3 [68.9-91.8] | 2.38 [1.04, 5.45] * | 73.8 [58.6-84.9] | 2.29 [1.13, 4.63] * | 69.0 [53.7-81.1] | 2.10 [1.07, 4.10] * |
| **Birth quarter** |  |  |  |  |  |  |  |
| Jan-Mar | 514 (25.2) | 80.0 [76.3-83.2] | 1.00 [reference] | 68.5 [64.3-72.4] | 1.00 [reference] | 62.5 [58.2-66.5] | 1.00 [reference] |
| Apr-Jun | 542 (26.6) | 82.8 [79.4-85.8] | 1.21 [0.89, 1.65] | 70.5 [66.5-74.2] | 1.10 [0.85, 1.43] | 68.3 [64.2-72.1] | 1.29 [1.00, 1.67] * |
| Jul-Sep | 457 (22.4) | 86.9 [83.5-89.7] | 1.66 [1.17, 2.35] * | 75.7 [71.6-79.4] | 1.43 [1.08, 1.90] * | 74.6 [70.4-78.4] | 1.77 [1.34, 2.33] * |
| Oct-Dec | 526 (25.8) | 81.2 [77.6-84.3] | 1.08 [0.79, 1.47] | 69.4 [65.3-73.2] | 1.04 [0.80, 1.36] | 64.6 [60.5-68.6] | 1.10 [0.85, 1.41] |
| **Sex of head of household** |  |  |  |  |  |  |  |
| Female | 332 (16.3) | 81.0 [76.4-84.9] | 1.00 [reference] | 67.2 [61.9-72.0] | 1.00 [reference] | 64.5 [59.2-69.4] | 1.00 [reference] |
| Male | 1707 (83.7) | 82.9 [81.0-84.6] | 1.13 [0.84, 1.53] | 71.6 [69.4-73.7] | 1.23 [0.96, 1.58] | 67.8 [65.6-70.0] | 1.16 [0.91, 1.49] |
| **Maternal age (years)** |  |  |  |  |  |  |  |
| 15-19 | 276 (13.5) | 81.9 [76.9-86.0] | 1.00 [reference] | 69.2 [63.5-74.4] | 1.00 [reference] | 67.0 [61.3-72.3] | 1.00 [reference] |
| 20-29 | 1046 (51.3) | 82.0 [79.6-84.2] | 1.01 [0.72, 1.43] | 70.3 [67.4-73.0] | 1.05 [0.79, 1.40] | 67.8 [64.9-70.5] | 1.03 [0.78, 1.37] |
| 30-39 | 586 (28.7) | 82.6 [79.3-85.5] | 1.05 [0.72, 1.53] | 71.0 [67.2-74.5] | 1.09 [0.80, 1.49] | 65.7 [61.8-69.4] | 0.94 [0.70, 1.28] |
| 40-49 | 131 (6.4) | 88.5 [81.9-93.0] | 1.71 [0.92, 3.18] | 78.6 [70.8-84.8] | 1.64 [1.00, 2.67] * | 71.0 [62.7-78.1] | 1.20 [0.77, 1.89] |
| **Maternal marital status** |  |  |  |  |  |  |  |
| Never in union | 68 (3.3) | 88.2 [78.2-94.0] | 1.00 [reference] | 75.0 [63.4-83.9] | 1.00 [reference] | 70.6 [58.7-80.2] | 1.00 [reference] |
| Divorced/widowed/Separated/ | 265 (13.0) | 75.5 [69.9-80.3] | 0.41 [0.19, 0.90] * | 61.9 [55.9-67.5] | 0.54 [0.30, 0.99] * | 59.2 [53.2-65.0] | 0.61 [0.34, 1.08] |
| married/with partner | 1706 (83.7) | 83.5 [81.6-85.2] | 0.67 [0.32, 1.42] | 72.1 [69.9-74.2] | 0.86 [0.49, 1.51] | 68.4 [66.2-70.6] | 0.90 [0.53, 1.54] |
| **Mother’s religion** |  |  |  |  |  |  |  |
| Muslim/Trad./ others | 725 (35.6) | 70.8 [67.3-74.0] | 1.00 [reference] | 54.6 [51.0-58.2] | 1.00 [reference] | 52.3 [48.6-55.9] | 1.00 [reference] |
| Christian | 1314 (64.4) | 89.1 [87.3-90.7] | 3.38 [2.67, 4.29] * | 79.8 [77.6-81.9] | 3.29 [2.70, 4.01] * | 75.6 [73.2-77.8] | 2.82 [2.33, 3.42] * |
| **Mother’s media exposure** |  |  |  |  |  |  |  |
| No | 1115 (54.7) | 75.4 [72.8-77.9] | 1.00 [reference] | 61.5 [58.6-64.3] | 1.00 [reference] | 57.0 [54.1-59.9] | 1.00 [reference] |
| Yes | 924 (45.3) | 91.2 [89.2-92.9] | 3.39 [2.60, 4.42] * | 82.1 [79.5-84.5] | 2.88 [2.34, 3.54] * | 79.7 [76.9-82.1] | 2.95 [2.42, 3.60] * |
| **Mother’s employment** |  |  |  |  |  |  |  |
| No | 176 (8.6) | 84.7 [78.5-89.3] | 1.00 [reference] | 76.7 [69.9-82.4] | 1.00 [reference] | 72.7 [65.7-78.8] | 1.00 [reference] |
| Yes (currently/in the past 1 year) | 1863 (91.4) | 82.4 [80.6-84.1] | 0.85 [0.55, 1.30] | 70.3 [68.2-72.3] | 0.72 [0.50, 1.03] | 66.8 [64.6-68.9] | 0.75 [0.53, 1.06] |
| **Maternal education** |  |  |  |  |  |  |  |
| None | 553 (27.1) | 65.5 [61.4-69.3] | 1.00 [reference] | 49.5 [45.4-53.7] | 1.00 [reference] | 47.0 [42.9-51.2] | 1.00 [reference] |
| Primary | 1064 (52.2) | 86.8 [84.7-88.7] | 3.48 [2.71, 4.47] * | 75.3 [72.6-77.8] | 3.10 [2.50, 3.85] * | 70.4 [67.6-73.1] | 2.68 [2.17, 3.31] * |
| Sec./higher | 422 (20.7) | 94.3 [91.7-96.2] | 8.75 [5.59, 13.69] * | 87.7 [84.2-90.5] | 7.25 [5.18, 10.13] * | 86.0 [82.4-89.0] | 6.93 [5.03, 9.57] * |
| **Mother’s use of mobile phone** |  |  |  |  |  |  |  |
| No | 1622 (79.5) | 79.8 [77.8-81.7] | 1.00 [reference] | 66.2 [63.9-68.5] | 1.00 [reference] | 62.4 [60.0-64.7] | 1.00 [reference] |
| Yes | 417 (20.5) | 93.3 [90.4-95.3] | 3.51 [2.35, 5.25] * | 89.0 [85.6-91.6] | 4.12 [2.98, 5.69] * | 86.3 [82.7-89.3] | 3.81 [2.83, 5.12] * |
| **Household owned a mosquito bednet** |  |  |  |  |  |  |  |
| No | 561 (27.5) | 83.4 [80.1-86.3] | 1.00 [reference] | 73.3 [69.4-76.8] | 1.00 [reference] | 68.3 [64.3-72.0] | 1.00 [reference] |
| Yes | 1478 (72.5) | 82.3 [80.2-84.1] | 0.92 [0.71, 1.20] | 70.0 [67.6-72.2] | 0.85 [0.68, 1.06] | 66.9 [64.5-69.3] | 0.94 [0.76, 1.16] |
| **Household wealth** |  |  |  |  |  |  |  |
| Poorer/poorest | 1017 (49.9) | 73.3 [70.4-75.9] | 1.00 [reference] | 58.7 [55.6-61.7] | 1.00 [reference] | 54.5 [51.4-57.5] | 1.00 [reference] |
| Middle | 363 (17.8) | 89.3 [85.6-92.1] | 3.03 [2.12, 4.35] * | 74.9 [70.2-79.1] | 2.10 [1.61, 2.75] * | 72.2 [67.3-76.5] | 2.17 [1.67, 2.81] * |
| Richer/richest | 659 (32.3) | 93.3 [91.1-95.0] | 5.10 [3.65, 7.14] * | 87.4 [84.6-89.7] | 4.88 [3.76, 6.34] * | 84.4 [81.4-86.9] | 4.51 [3.54, 5.76] * |
| **Household size** |  |  |  |  |  |  |  |
| Large (>=9) | 291 (14.3) | 83.8 [79.2-87.6] | 1.00 [reference] | 71.5 [66.0-76.4] | 1.00 [reference] | 69.1 [63.5-74.1] | 1.00 [reference] |
| Medium (5-8) | 1019 (50.0) | 81.6 [79.2-83.9] | 0.86 [0.60, 1.22] | 71.1 [68.3-73.9] | 0.98 [0.74, 1.31] | 65.7 [62.7-68.5] | 0.86 [0.65, 1.13] |
| Small (<=4) | 729 (35.8) | 83.4 [80.5-85.9] | 0.97 [0.67, 1.40] | 70.2 [66.8-73.4] | 0.94 [0.70, 1.27] | 68.9 [65.4-72.1] | 0.99 [0.74, 1.33] |
| **Length of stay in place of residence** |  |  |  |  |  |  |  |
| <1 year/visitor | 51 (2.5) | 88.2 [76.2-94.6] | 1.00 [reference] | 78.4 [65.1-87.6] | 1.00 [reference] | 74.5 [60.9-84.6] | 1.00 [reference] |
| 1-3 year | 205 (10.1) | 90.7 [85.9-94.0] | 1.31 [0.49, 3.46] | 76.6 [70.3-81.9] | 0.90 [0.43, 1.89] | 78.5 [72.4-83.6] | 1.25 [0.61, 2.55] |
| 4-5 year | 132 (6.5) | 84.1 [76.8-89.4] | 0.70 [0.27, 1.86] | 72.7 [64.5-79.6] | 0.73 [0.34, 1.58] | 65.9 [57.4-73.5] | 0.66 [0.32, 1.37] |
| >5 year/always | 1651 (81.0) | 81.3 [79.3-83.1] | 0.58 [0.24, 1.37] | 69.8 [67.5-71.9] | 0.63 [0.32, 1.25] | 65.8 [63.5-68.0] | 0.66 [0.35, 1.24] |
| **Urban/rural** |  |  |  |  |  |  |  |
| Rural | 1685 (82.6) | 80.2 [78.2-82.0] | 1.00 [reference] | 67.4 [65.1-69.6] | 1.00 [reference] | 63.3 [61.0-65.6] | 1.00 [reference] |
| Urban | 354 (17.4) | 94.1 [91.1-96.1] | 3.92 [2.48, 6.19] * | 87.3 [83.4-90.4] | 3.32 [2.39, 4.61] * | 86.2 [82.2-89.4] | 3.61 [2.62, 4.95] * |
| **Region** |  |  |  |  |  |  |  |
| Anal./Vaki./Ita./Bon./Diana/Sava | 557 (27.3) | 91.2 [88.5-93.3] | 1.00 [reference] | 82.8 [79.4-85.7] | 1.00 [reference] | 76.7 [73.0-80.0] | 1.00 [reference] |
| Atsin./Analan./Alao. Man. | 229 (11.2) | 90.0 [85.3-93.2] | 0.86 [0.51, 1.45] | 81.7 [76.1-86.2] | 0.93 [0.62, 1.38] | 80.8 [75.2-85.4] | 1.28 [0.87, 1.88] |
| Ats. And./Androy/Anosy/Menabe | 366 (17.9) | 67.8 [62.8-72.4] | 0.20 [0.14, 0.29] * | 53.8 [48.7-58.9] | 0.24 [0.18, 0.33] * | 52.5 [47.3-57.5] | 0.34 [0.25, 0.45] * |
| Boeny/Sofia/Bets./Melaky | 335 (16.4) | 81.5 [77.0-85.3] | 0.42 [0.28, 0.64] * | 63.9 [58.6-68.9] | 0.37 [0.27, 0.50] * | 61.2 [55.9-66.3] | 0.48 [0.36, 0.64] * |
| Hau. Mat./Amo. Man./Vat. Fit./Ihor./Ats. Ats. | 552 (27.1) | 81.3 [77.9-84.4] | 0.42 [0.29, 0.60] * | 69.9 [66.0-73.6] | 0.48 [0.36, 0.64] * | 65.8 [61.7-69.6] | 0.58 [0.45, 0.76] * |
| **Conflict area** |  |  |  |  |  |  |  |
| No | 1817 (89.1) | 83.9 [82.1-85.5] | 2.02 [1.47, 2.77] * | 72.4 [70.3-74.4] | 1.00 [reference] | 68.7 [66.6-70.8] | 1.00 [reference] |
| Yes | 222 (10.9) | 72.1 [65.8-77.6] | 1.00 [reference] | 58.1 [51.5-64.4] | 0.53 [0.40, 0.70] * | 55.4 [48.8-61.8] | 0.57 [0.43, 0.75] * |
| **Urban slum** |  |  |  |  |  |  |  |
| No | 1969 (96.6) | 82.3 [80.5-83.9] | 0.44 [0.19, 1.01] | 70.5 [68.4-72.5] | 1.00 [reference] | 66.9 [64.8-68.9] | 1.00 [reference] |
| Yes | 70 (3.4) | 91.4 [82.2-96.1] | 1.00 [reference] | 81.4 [70.6-88.9] | 1.84 [1.00, 3.38] | 78.6 [67.4-86.7] | 1.82 [1.02, 3.24] * |
| **Travel time (mins)** |  |  |  |  |  |  |  |
| Higher (303.1 - 1340.75) | 678 (33.3) | 74.0 [70.6-77.2] | 1.00 [reference] | 57.1 [53.3-60.8] | 1.00 [reference] | 53.7 [49.9-57.4] | 1.00 [reference] |
| Medium (119.1 – 303) | 675 (33.1) | 83.3 [80.2-85.9] | 1.74 [1.34, 2.27] * | 71.9 [68.3-75.1] | 1.92 [1.53, 2.41] * | 68.7 [65.1-72.1] | 1.90 [1.52, 2.37] * |
| Lower (0 – 119) | 686 (33.6) | 90.4 [87.9-92.4] | 3.29 [2.42, 4.47] * | 83.5 [80.6-86.1] | 3.81 [2.96, 4.91] * | 79.3 [76.1-82.2] | 3.30 [2.60, 4.20] * |

*Estimated crude odds ratio significant

**Table J: Bivariate analysis of factors associated with receipt of DPT1, DTP3 and MCV1 for Mozambique, 2011 DHS**

|  |  | **DTP1** |  | **DTP3** |  | **MCV1** |  |
| --- | --- | --- | --- | --- | --- | --- | --- |
| **Characteristics and categories** | **Number (%) in category** | **Percentage vaccinated**  **(95% CI)** | **cOR [95% CI]** | **Percentage vaccinated**  **(95% CI)** | **cOR [95% CI]** | **Percentage vaccinated**  **(95% CI)** | **cOR [95% CI]** |
| Total number of children | 2110 |  |  |  |  |  |  |
| **Sex of child** |  |  |  |  |  |  |  |
| Male | 1019 (48.3) | 92.2 [90.3-93.7] | 1.00 [reference] | 78.9 [76.3-81.3] | 1.00 [reference] | 83.0 [80.6-85.2] | 1.00 [reference] |
| Female | 1091 (51.7) | 92.1 [90.4-93.6] | 1.00 [0.73, 1.37] | 80.4 [77.9-82.6] | 1.10 [0.89, 1.35] | 84.0 [81.7-86.0] | 1.07 [0.85, 1.35] |
| **Birth order** |  |  |  |  |  |  |  |
| 1-2 | 898 (42.6) | 93.7 [91.9-95.1] | 1.00 [reference] | 83.3 [80.7-85.6] | 1.00 [reference] | 87.1 [84.7-89.1] | 1.00 [reference] |
| 3-5 | 811 (38.4) | 90.9 [88.7-92.7] | 0.68 [0.47, 0.97] * | 78.2 [75.2-80.9] | 0.72 [0.56, 0.91] * | 83.2 [80.5-85.6] | 0.74 [0.56, 0.96] * |
| >5 | 401 (19.0) | 91.3 [88.1-93.7] | 0.71 [0.46, 1.10] | 74.6 [70.1-78.6] | 0.59 [0.44, 0.78] * | 76.1 [71.6-80.0] | 0.47 [0.35, 0.64] * |
| **Skilled birth attendance (SBA)** |  |  |  |  |  |  |  |
| No | 787 (37.3) | 85.6 [83.0-87.9] | 1.00 [reference] | 66.8 [63.5-70.0] | 1.00 [reference] | 73.2 [70.0-76.2] | 1.00 [reference] |
| Yes | 1323 (62.7) | 96.0 [94.8-96.9] | 4.02 [2.86, 5.64] * | 87.3 [85.4-89.0] | 3.41 [2.74, 4.25] * | 89.6 [87.9-91.2] | 3.17 [2.50, 4.02] * |
| **Antenatal care attendance (ANC)** |  |  |  |  |  |  |  |
| No ANC | 142 (6.7) | 54.2 [46.0-62.2] | 1.00 [reference] | 33.1 [25.9-41.2] | 1.00 [reference] | 50.0 [41.8-58.2] | 1.00 [reference] |
| 1-3 | 804 (38.1) | 93.4 [91.5-94.9] | 11.96 [7.77, 18.42] * | 80.2 [77.3-82.8] | 8.20 [5.55, 12.11] * | 83.2 [80.5-85.6] | 4.96 [3.40, 7.23] * |
| >=4 | 1164 (55.2) | 95.9 [94.6-96.9] | 19.63 [12.66, 30.43] * | 85.0 [82.8-86.9] | 11.42 [7.78, 16.78] * | 87.8 [85.8-89.6] | 7.20 [4.96, 10.45] * |
| **Birth quarter** |  |  |  |  |  |  |  |
| Jan-Mar | 579 (27.4) | 92.1 [89.6-94.0] | 1.00 [reference] | 81.3 [78.0-84.3] | 1.00 [reference] | 85.7 [82.6-88.3] | 1.00 [reference] |
| Apr-Jun | 579 (27.4) | 91.4 [88.8-93.4] | 0.91 [0.60, 1.39] | 79.8 [76.3-82.9] | 0.91 [0.68, 1.21] | 82.6 [79.2-85.4] | 0.79 [0.58, 1.09] |
| Jul-Sep | 508 (24.1) | 93.3 [90.8-95.2] | 1.20 [0.76, 1.91] | 77.4 [73.5-80.8] | 0.78 [0.58, 1.05] | 79.9 [76.2-83.2] | 0.67 [0.48, 0.92] * |
| Oct-Dec | 444 (21.0) | 91.9 [89.0-94.1] | 0.98 [0.62, 1.54] | 80.0 [76.0-83.4] | 0.91 [0.67, 1.25] | 86.0 [82.5-89.0] | 1.03 [0.72, 1.47] |
| **Mother’s TT vaccinations before birth** |  |  |  |  |  |  |  |
| 0 | 409 (19.4) | 78.0 [73.7-81.7] | 1.00 [reference] | 62.6 [57.8-67.2] | 1.00 [reference] | 69.2 [64.5-73.5] | 1.00 [reference] |
| 1-2 | 1065 (50.5) | 95.8 [94.4-96.8] | 6.39 [4.38, 9.34] * | 82.8 [80.4-85.0] | 2.88 [2.23, 3.72] * | 85.4 [83.2-87.4] | 2.61 [1.99, 3.43] * |
| 3-4 | 563 (26.7) | 95.7 [93.7-97.1] | 6.34 [3.96, 10.15] * | 85.8 [82.7-88.4] | 3.61 [2.65, 4.92] * | 89.9 [87.1-92.1] | 3.95 [2.80, 5.58] * |
| >=5 | 73 (3.5) | 90.4 [81.2-95.4] | 2.66 [1.18, 6.00] * | 82.2 [71.7-89.4] | 2.76 [1.47, 5.19] * | 86.3 [76.4-92.5] | 2.80 [1.39, 5.65] * |
| **Sex of head of household** |  |  |  |  |  |  |  |
| Female | 651 (30.9) | 92.9 [90.7-94.7] | 1.00 [reference] | 81.0 [77.8-83.8] | 1.00 [reference] | 85.4 [82.5-87.9] | 1.00 [reference] |
| Male | 1459 (69.1) | 91.8 [90.2-93.1] | 0.85 [0.60, 1.21] | 79.1 [76.9-81.1] | 0.89 [0.71, 1.12] | 82.7 [80.6-84.5] | 0.81 [0.63, 1.05] |
| **Maternal age (years)** |  |  |  |  |  |  |  |
| 15-19 | 267 (12.7) | 94.4 [90.9-96.6] | 1.00 [reference] | 85.8 [81.0-89.5] | 1.00 [reference] | 85.8 [81.0-89.5] | 1.00 [reference] |
| 20-29 | 1070 (50.7) | 92.4 [90.7-93.9] | 0.73 [0.41, 1.28] | 80.3 [77.8-82.6] | 0.68 [0.46, 0.98] * | 85.2 [83.0-87.2] | 0.96 [0.65, 1.40] |
| 30-39 | 631 (29.9) | 91.3 [88.8-93.2] | 0.62 [0.35, 1.12] | 78.0 [74.6-81.0] | 0.59 [0.40, 0.87] * | 81.3 [78.1-84.2] | 0.72 [0.48, 1.07] |
| 40-49 | 142 (6.7) | 89.4 [83.2-93.5] | 0.50 [0.24, 1.06] | 71.1 [63.1-78.0] | 0.41 [0.25, 0.67] * | 76.1 [68.4-82.4] | 0.53 [0.31, 0.88] * |
| **Maternal marital status** |  |  |  |  |  |  |  |
| Never in union | 129 (6.1) | 94.6 [89.1-97.4] | 1.00 [reference] | 86.8 [79.8-91.7] | 1.00 [reference] | 91.5 [85.3-95.2] | 1.00 [reference] |
| Divorced/widowed/ separated | 199 (9.4) | 93.0 [88.5-95.8] | 0.76 [0.30, 1.93] | 79.4 [73.2-84.5] | 0.58 [0.32, 1.08] | 87.4 [82.1-91.4] | 0.65 [0.31, 1.37] |
| Married/with partner | 1782 (84.5) | 91.9 [90.5-93.0] | 0.65 [0.30, 1.41] | 79.2 [77.2-81.0] | 0.58 [0.34, 0.97] * | 82.5 [80.7-84.2] | 0.44 [0.23, 0.82] * |
| **Mother had problem seeking medical advice or treatment** |  |  |  |  |  |  |  |
| Had problem | 813 (38.5) | 96.4 [94.9-97.5] | 1.00 [reference] | 86.5 [83.9-88.7] | 1.00 [reference] | 89.3 [87.0-91.2] | 1.00 [reference] |
| Had no problem | 1297 (61.5) | 89.4 [87.6-91.0] | 0.31 [0.21, 0.47] * | 75.4 [73.0-77.7] | 0.48 [0.38, 0.61] * | 79.9 [77.6-82.0] | 0.48 [0.37, 0.62] * |
| **Maternal education** |  |  |  |  |  |  |  |
| None | 697 (33.0) | 88.7 [86.1-90.8] | 1.00 [reference] | 73.6 [70.2-76.7] | 1.00 [reference] | 76.0 [72.7-79.1] | 1.00 [reference] |
| Primary | 1066 (50.5) | 93.1 [91.4-94.4] | 1.71 [1.23, 2.39] * | 80.9 [78.4-83.1] | 1.52 [1.21, 1.90] * | 84.6 [82.3-86.7] | 1.73 [1.36, 2.20] * |
| Sec./higher | 347 (16.4) | 96.3 [93.7-97.8] | 3.28 [1.80, 5.99] * | 88.2 [84.3-91.2] | 2.68 [1.85, 3.86] * | 95.1 [92.3-96.9] | 6.12 [3.65, 10.26] * |
| **Mother’s employment status** |  |  |  |  |  |  |  |
| No | 1155 (54.7) | 91.7 [90.0-93.1] | 1.00 [reference] | 80.4 [78.0-82.6] | 1.00 [reference] | 83.5 [81.3-85.6] | 1.00 [reference] |
| Yes (currently/in the past 1 year) | 955 (45.3) | 92.7 [90.8-94.2] | 1.15 [0.83, 1.58] | 78.7 [76.0-81.2] | 0.90 [0.73, 1.11] | 83.5 [81.0-85.7] | 0.99 [0.79, 1.25] |
| **Mother’s media exposure** |  |  |  |  |  |  |  |
| No | 1091 (51.7) | 89.6 [87.6-91.2] | 1.00 [reference] | 75.3 [72.6-77.7] | 1.00 [reference] | 79.5 [77.0-81.8] | 1.00 [reference] |
| Yes | 1019 (48.3) | 94.9 [93.4-96.1] | 2.17 [1.54, 3.05] * | 84.4 [82.0-86.5] | 1.78 [1.43, 2.21] * | 87.8 [85.7-89.7] | 1.86 [1.47, 2.37] * |
| **Mother’s use of mobile phone** |  |  |  |  |  |  |  |
| No | 1203 (57.0) | 89.2 [87.3-90.8] | 1.00 [reference] | 73.6 [71.0-76.0] | 1.00 [reference] | 78.2 [75.8-80.5] | 1.00 [reference] |
| Yes | 907 (43.0) | 96.0 [94.5-97.1] | 2.93 [2.01, 4.29] * | 87.8 [85.5-89.7] | 2.58 [2.03, 3.26] * | 90.5 [88.4-92.3] | 2.66 [2.05, 3.45] * |
| **Mother’s land ownership** |  |  |  |  |  |  |  |
| Does not own land | 866 (41.0) | 93.2 [91.3-94.7] | 1.00 [reference] | 82.1 [79.4-84.5] | 1.00 [reference] | 85.2 [82.7-87.4] | 1.00 [reference] |
| Alone or jointly or both | 1244 (59.0) | 91.4 [89.7-92.8] | 0.78 [0.56, 1.08] | 78.0 [75.6-80.2] | 0.77 [0.62, 0.96] * | 82.3 [80.1-84.3] | 0.81 [0.64, 1.02] |
| **Mother had health insurance** |  |  |  |  |  |  |  |
| No | 2071 (98.2) | 92.0 [90.8-93.1] | 1.00 [reference] | 79.4 [77.6-81.1] | 1.00 [reference] | 83.2 [81.5-84.7] | 1.00 [reference] |
| Yes | 39 (1.8) | 97.4 [83.9-99.6] | 3.29 [0.45, 24.11] | 94.9 [81.7-98.7] | 4.81 [1.15, 20.02] * | 0.0 [empty] | [empty] |
| **Household owned a mosquito bednet** |  |  |  |  |  |  |  |
| No | 760 (36.0) | 88.9 [86.5-91.0] | 1.00 [reference] | 75.0 [71.8-78.0] | 1.00 [reference] | 78.7 [75.6-81.5] | 1.00 [reference] |
| Yes | 1350 (64.0) | 93.9 [92.5-95.1] | 1.92 [1.40, 2.64] * | 82.3 [80.2-84.2] | 1.55 [1.25, 1.92] * | 86.2 [84.3-88.0] | 1.70 [1.34, 2.14] * |
| **Mother’s ethnicity** |  |  |  |  |  |  |  |
| Emakhuwa/Portuguese | 507 (24.0) | 92.9 [90.3-94.8] | 1.00 [reference] | 80.5 [76.8-83.7] | 1.00 [reference] | 85.0 [81.6-87.9] | 1.00 [reference] |
| Elomwe/Echuwabo | 264 (12.5) | 79.5 [74.2-84.0] | 0.30 [0.19, 0.47] * | 63.6 [57.7-69.2] | 0.42 [0.30, 0.59] * | 69.3 [63.5-74.6] | 0.40 [0.28, 0.57] * |
| Others | 501 (23.7) | 93.0 [90.4-94.9] | 1.02 [0.63, 1.65] | 80.4 [76.7-83.7] | 1.00 [0.73, 1.36] | 86.4 [83.1-89.2] | 1.12 [0.79, 1.60] |
| Shona/Cinyungwe | 235 (11.1) | 95.7 [92.3-97.7] | 1.72 [0.84, 3.53] | 80.4 [74.9-85.0] | 1.00 [0.68, 1.47] | 84.3 [79.0-88.4] | 0.94 [0.62, 1.45] |
| Xichangana/Cisena | 603 (28.6) | 94.9 [92.8-96.4] | 1.41 [0.86, 2.31] | 85.1 [82.0-87.7] | 1.38 [1.01, 1.89] * | 85.7 [82.7-88.3] | 1.06 [0.76, 1.48] |
| **Household wealth** |  |  |  |  |  |  |  |
| Poorer/poorest | 806 (38.2) | 87.1 [84.6-89.2] | 1.00 [reference] | 69.2 [66.0-72.3] | 1.00 [reference] | 74.8 [71.7-77.7] | 1.00 [reference] |
| Middle | 419 (19.9) | 91.9 [88.9-94.1] | 1.68 [1.12, 2.52] * | 81.4 [77.4-84.8] | 1.94 [1.46, 2.59] * | 82.3 [78.4-85.7] | 1.57 [1.17, 2.11] * |
| Richer/richest | 885 (41.9) | 96.8 [95.5-97.8] | 4.53 [2.95, 6.96] * | 88.4 [86.1-90.3] | 3.37 [2.62, 4.35] * | 92.0 [90.0-93.6] | 3.86 [2.89, 5.16] * |
| **Mother owned a bank account** |  |  |  |  |  |  |  |
| No | 1727 (81.8) | 91.1 [89.7-92.4] | 1.00 [reference] | 77.5 [75.5-79.4] | 1.00 [reference] | 81.1 [79.2-82.9] | 1.00 [reference] |
| Yes | 383 (18.2) | 96.6 [94.2-98.0] | 2.77 [1.55, 4.93] * | 89.3 [85.8-92.0] | 2.42 [1.72, 3.41] * | 94.3 [91.4-96.2] | 3.82 [2.44, 5.97] * |
| **Household size** |  |  |  |  |  |  |  |
| Large (>=9) | 326 (15.5) | 94.8 [91.8-96.7] | 1.00 [reference] | 83.7 [79.3-87.4] | 1.00 [reference] | 86.5 [82.3-89.8] | 1.00 [reference] |
| Medium (5-8) | 1111 (52.7) | 90.7 [88.9-92.3] | 0.54 [0.32, 0.91] * | 78.2 [75.7-80.5] | 0.70 [0.50, 0.97] * | 82.4 [80.0-84.5] | 0.73 [0.51, 1.04] |
| Small (<=4) | 673 (31.9) | 93.2 [91.0-94.8] | 0.75 [0.42, 1.33] | 80.1 [76.9-82.9] | 0.78 [0.55, 1.11] | 84.0 [81.0-86.5] | 0.82 [0.56, 1.19] |
| **Urban/rural** |  |  |  |  |  |  |  |
| Rural | 1449 (68.7) | 90.2 [88.6-91.6] | 1.00 [reference] | 76.0 [73.7-78.1] | 1.00 [reference] | 79.6 [77.4-81.6] | 1.00 [reference] |
| Urban | 661 (31.3) | 96.4 [94.6-97.6] | 2.88 [1.85, 4.49] * | 87.7 [85.0-90.0] | 2.26 [1.74, 2.94] * | 92.1 [89.8-94.0] | 3.01 [2.20, 4.10] * |
| **Conflict area** |  |  |  |  |  |  |  |
| Yes | 13 (0.6) | 92.3 [60.9-98.9] | 1.00 [reference] | 69.2 [40.9-88.0] | 1.00 [reference] | 92.3 [60.9-98.9] | 1.00 [reference] |
| No | 2097 (99.4) | 92.1 [90.9-93.2] | 0.98 [0.13, 7.55] | 79.7 [78.0-81.4] | 1.75 [0.54, 5.71] | 83.5 [81.8-85.0] | 0.42 [0.05, 3.24] |
| **Urban slum** |  |  |  |  |  |  |  |
| Yes | 128 (6.1) | 95.3 [90.0-97.9] | 1.00 [reference] | 89.8 [83.3-94.0] | 1.00 [reference] | 87.5 [80.6-92.2] | 1.00 [reference] |
| No | 1982 (93.9) | 91.9 [90.6-93.0] | 0.56 [0.24, 1.29] | 79.0 [77.2-80.7] | 0.43 [0.24, 0.76] * | 83.2 [81.5-84.8] | 0.71 [0.42, 1.21] |
| **Travel time (mins)** |  |  |  |  |  |  |  |
| Higher (72.0 – 396) | 697 (33.0) | 94.7 [92.8-96.1] | 1.00 [reference] | 85.7 [82.8-88.1] | 1.00 [reference] | 86.8 [84.1-89.1] | 1.00 [reference] |
| Medium (10.2 - 71.9) | 700 (33.2) | 94.0 [92.0-95.5] | 0.88 [0.56, 1.38] | 83.7 [80.8-86.3] | 0.86 [0.64, 1.15] | 85.7 [82.9-88.1] | 0.91 [0.67, 1.24] |
| Lower (0 - 10.1) | 713 (33.8) | 87.8 [85.2-90.0] | 0.40 [0.27, 0.60] * | 69.8 [66.4-73.1] | 0.39 [0.30, 0.51] * | 78.1 [74.9-81.0] | 0.54 [0.41, 0.72] * |

*Estimated crude odds ratio significant

**Table K: Bivariate analysis of factors associated with receipt of DPT1, DTP3 and MCV1 for Nigeria, 2018 DHS**

|  |  | **DTP1** |  | **DTP3** |  | **MCV1** |  |
| --- | --- | --- | --- | --- | --- | --- | --- |
| **Characteristics and categories** | **Number (%) in category** | **Percentage vaccinated**  **(95% CI)** | **cOR [95% CI]** | **Percentage vaccinated**  **(95% CI)** | **cOR [95% CI]** | **Percentage vaccinated**  **(95% CI)** | **cOR [95% CI]** |
| Total number of children | 5704 |  |  |  |  |  |  |
| **Sex of child** |  |  |  |  |  |  |  |
| Male | 2966 (52.0) | 63.5 [61.8-65.2] | 1.00 [reference] | 49.4 [47.6-51.2] | 1.00 [reference] | 52.5 [50.7-54.3] | 1.00 [reference] |
| Female | 2738 (48.0) | 64.2 [62.4-66.0] | 1.03 [0.93, 1.15] | 50.0 [48.1-51.9] | 1.02 [0.92, 1.14] | 52.8 [51.0-54.7] | 1.01 [0.91, 1.13] |
| **Birth order** |  |  |  |  |  |  |  |
| 1-2 | 2115 (37.1) | 69.2 [67.2-71.1] | 1.00 [reference] | 55.5 [53.3-57.6] | 1.00 [reference] | 56.3 [54.1-58.4] | 1.00 [reference] |
| 3-5 | 2236 (39.2) | 65.8 [63.8-67.8] | 0.86 [0.76, 0.98] * | 51.1 [49.0-53.1] | 0.84 [0.74, 0.94] * | 55.9 [53.8-57.9] | 0.98 [0.87, 1.11] |
| >5 | 1353 (23.7) | 52.3 [49.7-55.0] | 0.49 [0.42, 0.56] * | 38.4 [35.8-41.0] | 0.50 [0.43, 0.57] * | 41.8 [39.2-44.4] | 0.56 [0.49, 0.64] * |
| **Skilled birth attendance (SBA)** |  |  |  |  |  |  |  |
| No | 3186 (55.9) | 46.0 [44.2-47.7] | 1.00 [reference] | 31.3 [29.7-32.9] | 1.00 [reference] | 37.2 [35.5-38.9] | 1.00 [reference] |
| Yes | 2518 (44.1) | 86.5 [85.1-87.8] | 7.56 [6.61, 8.64] * | 73.0 [71.2-74.7] | 5.92 [5.28, 6.65] * | 72.2 [70.5-74.0] | 4.39 [3.92, 4.92] * |
| **Antenatal care attendance (ANC)** |  |  |  |  |  |  |  |
| No ANC | 1420 (24.9) | 26.6 [24.4-29.0] | 1.00 [reference] | 16.7 [14.8-18.7] | 1.00 [reference] | 22.7 [20.6-24.9] | 1.00 [reference] |
| 1-3 | 962 (16.9) | 58.8 [55.7-61.9] | 3.94 [3.31, 4.69] * | 41.9 [38.8-45.0] | 3.60 [2.98, 4.35] * | 46.3 [43.1-49.4] | 2.94 [2.46, 3.50] * |
| >=4 | 3322 (58.2) | 81.2 [79.9-82.5] | 11.94 [10.32, 13.83] * | 66.0 [64.4-67.6] | 9.71 [8.30, 11.36] * | 67.3 [65.7-68.9] | 7.03 [6.09, 8.12] * |
| **Birth quarter** |  |  |  |  |  |  |  |
| Jan-Mar | 1283 (22.5) | 65.2 [62.5-67.7] | 1.00 [reference] | 49.8 [47.1-52.5] | 1.00 [reference] | 54.2 [51.4-56.9] | 1.00 [reference] |
| Apr-Jun | 1594 (27.9) | 64.9 [62.5-67.2] | 0.99 [0.85, 1.15] | 51.0 [48.5-53.5] | 1.05 [0.91, 1.22] | 54.0 [51.6-56.5] | 0.99 [0.86, 1.15] |
| Jul-Sep | 1558 (27.3) | 59.3 [56.8-61.7] | 0.78 [0.67, 0.91] * | 44.5 [42.1-47.0] | 0.81 [0.70, 0.94] * | 46.3 [43.8-48.8] | 0.73 [0.63, 0.85] * |
| Oct-Dec | 1269 (22.2) | 66.9 [64.3-69.4] | 1.08 [0.92, 1.27] | 54.2 [51.5-56.9] | 1.19 [1.02, 1.39] * | 57.3 [54.5-60.0] | 1.13 [0.97, 1.33] |
| **Mother’s TT vaccinations before birth** |  |  |  |  |  |  |  |
| 0 | 1706 (29.9) | 32.1 [29.9-34.4] | 1.00 [reference] | 20.8 [18.9-22.8] | 1.00 [reference] | 26.6 [24.5-28.7] | 1.00 [reference] |
| 1-2 | 2984 (52.3) | 75.2 [73.7-76.8] | 6.42 [5.63, 7.32] * | 58.9 [57.1-60.6] | 5.45 [4.75, 6.25] * | 61.2 [59.5-63.0] | 4.37 [3.83, 4.98] * |
| 3-4 | 944 (16.5) | 84.1 [81.6-86.3] | 11.19 [9.14, 13.69] * | 71.7 [68.8-74.5] | 9.65 [8.03, 11.59] * | 71.4 [68.4-74.2] | 6.90 [5.78, 8.25] * |
| >=5 | 70 (1.2) | 80.0 [69.0-87.8] | 8.45 [4.66, 15.32] * | 64.3 [52.5-74.6] | 6.85 [4.14, 11.32] * | 71.4 [59.8-80.8] | 6.92 [4.07, 11.74] * |
| **Sex of head of household** |  |  |  |  |  |  |  |
| Female | 568 (10.0) | 79.6 [76.1-82.7] | 1.00 [reference] | 63.7 [59.7-67.6] | 1.00 [reference] | 63.6 [59.5-67.4] | 1.00 [reference] |
| Male | 5136 (90.0) | 62.1 [60.8-63.4] | 0.42 [0.34, 0.52] * | 48.1 [46.8-49.5] | 0.53 [0.44, 0.63] * | 51.5 [50.1-52.8] | 0.61 [0.51, 0.73] * |
| **Maternal age (years)** |  |  |  |  |  |  |  |
| 15-19 | 342 (6.0) | 47.7 [42.4-53.0] | 1.00 [reference] | 31.3 [26.6-36.4] | 1.00 [reference] | 32.2 [27.4-37.3] | 1.00 [reference] |
| 20-29 | 2835 (49.7) | 64.0 [62.2-65.7] | 1.95 [1.55, 2.44] * | 48.6 [46.8-50.4] | 2.08 [1.63, 2.64] * | 52.3 [50.5-54.1] | 2.31 [1.82, 2.94] * |
| 30-39 | 2100 (36.8) | 68.6 [66.6-70.5] | 2.40 [1.90, 3.02] * | 55.9 [53.7-58.0] | 2.78 [2.18, 3.55] * | 58.3 [56.2-60.4] | 2.95 [2.31, 3.76] * |
| 40-49 | 427 (7.5) | 53.2 [48.4-57.9] | 1.25 [0.94, 1.66] | 41.2 [36.6-46.0] | 1.54 [1.14, 2.08] * | 43.8 [39.2-48.5] | 1.64 [1.22, 2.21] * |
| **Maternal marital status** |  |  |  |  |  |  |  |
| Never in union | 148 (2.6) | 79.7 [72.5-85.4] | 1.00 [reference] | 62.2 [54.1-69.6] | 1.00 [reference] | 64.2 [56.2-71.5] | 1.00 [reference] |
| Divorced/widowed/separated | 134 (2.3) | 63.4 [55.0-71.1] | 0.44 [0.26, 0.75] * | 46.3 [38.0-54.7] | 0.52 [0.33, 0.84] * | 58.2 [49.7-66.3] | 0.78 [0.48, 1.26] |
| Married/with partner | 5422 (95.1) | 63.4 [62.2-64.7] | 0.44 [0.29, 0.66] * | 49.4 [48.1-50.8] | 0.59 [0.42, 0.83] * | 52.2 [50.9-53.5] | 0.61 [0.43, 0.86] * |
| **Mother had problem seeking medical advice or treatment** |  |  |  |  |  |  |  |
| Had problem | 2518 (44.1) | 71.6 [69.8-73.3] | 1.00 [reference] | 57.9 [56.0-59.8] | 1.00 [reference] | 60.1 [58.2-62.0] | 1.00 [reference] |
| Had no problem | 3186 (55.9) | 57.8 [56.0-59.5] | 0.54 [0.48, 0.61] * | 43.2 [41.5-44.9] | 0.55 [0.50, 0.61] * | 46.8 [45.1-48.5] | 0.58 [0.53, 0.65] * |
| **Maternal education** |  |  |  |  |  |  |  |
| None | 2517 (44.1) | 39.3 [37.4-41.2] | 1.00 [reference] | 24.9 [23.3-26.6] | 1.00 [reference] | 32.9 [31.1-34.8] | 1.00 [reference] |
| Primary | 832 (14.6) | 71.8 [68.6-74.7] | 3.93 [3.31, 4.66] * | 54.2 [50.8-57.6] | 3.57 [3.03, 4.20] * | 54.6 [51.2-57.9] | 2.45 [2.08, 2.87] * |
| Sec./higher | 2355 (41.3) | 87.4 [86.0-88.7] | 10.72 [9.27, 12.40] * | 74.6 [72.8-76.3] | 8.84 [7.76, 10.06] * | 73.1 [71.2-74.8] | 5.53 [4.89, 6.25] * |
| **Mother’s religion** |  |  |  |  |  |  |  |
| Islam | 3398 (59.6) | 49.4 [47.8-51.1] | 1.00 [reference] | 34.8 [33.3-36.5] | 1.00 [reference] | 41.3 [39.6-42.9] | 1.00 [reference] |
| Christian/Trad./others | 2306 (40.4) | 85.1 [83.6-86.5] | 5.85 [5.12, 6.68] * | 71.6 [69.7-73.4] | 4.70 [4.19, 5.28] * | 69.5 [67.6-71.3] | 3.24 [2.90, 3.62] * |
| **Mother’s media exposure** |  |  |  |  |  |  |  |
| No | 3522 (61.7) | 54.3 [52.6-55.9] | 1.00 [reference] | 39.8 [38.2-41.4] | 1.00 [reference] | 43.7 [42.0-45.3] | 1.00 [reference] |
| Yes | 2182 (38.3) | 79.3 [77.6-81.0] | 3.23 [2.86, 3.65] * | 65.6 [63.6-67.6] | 2.89 [2.58, 3.23] * | 67.2 [65.2-69.1] | 2.64 [2.36, 2.95] * |
| **Mother’s use of mobile phone or internet** |  |  |  |  |  |  |  |
| No | 2916 (51.1) | 47.9 [46.1-49.8] | 1.00 [reference] | 33.2 [31.5-34.9] | 1.00 [reference] | 38.5 [36.8-40.3] | 1.00 [reference] |
| Yes | 2788 (48.9) | 80.5 [79.0-82.0] | 4.49 [3.99, 5.05] * | 67.0 [65.2-68.7] | 4.09 [3.66, 4.56] * | 67.4 [65.7-69.1] | 3.30 [2.96, 3.68] * |
| **Mother’s land ownership** |  |  |  |  |  |  |  |
| Does not own land | 4933 (86.5) | 62.2 [60.8-63.5] | 1.00 [reference] | 47.8 [46.4-49.2] | 1.00 [reference] | 51.3 [49.9-52.7] | 1.00 [reference] |
| Alone or jointly or both | 771 (13.5) | 74.6 [71.4-77.5] | 1.78 [1.50, 2.12] * | 62.0 [58.5-65.4] | 1.78 [1.53, 2.09] * | 61.6 [58.1-65.0] | 1.53 [1.31, 1.78] * |
| **Mother had knowledge of malaria** |  |  |  |  |  |  |  |
| No | 370 (6.5) | 61.4 [56.3-66.2] | 1.00 [reference] | 43.5 [38.5-48.6] | 1.00 [reference] | 47.6 [42.5-52.7] | 1.00 [reference] |
| Yes | 5334 (93.5) | 64.0 [62.7-65.3] | 1.12 [0.90, 1.39] | 50.1 [48.8-51.5] | 1.30 [1.05, 1.61] * | 53.0 [51.7-54.4] | 1.24 [1.01, 1.54] * |
| **Mother’s employment status** |  |  |  |  |  |  |  |
| No | 1677 (29.4) | 50.6 [48.2-53.0] | 1.00 [reference] | 37.6 [35.3-39.9] | 1.00 [reference] | 41.0 [38.7-43.4] | 1.00 [reference] |
| Yes (currently/in the past 1 year) | 4027 (70.6) | 69.4 [67.9-70.8] | 2.21 [1.97, 2.48] * | 54.7 [53.2-56.3] | 2.01 [1.79, 2.26] * | 57.5 [56.0-59.0] | 1.95 [1.73, 2.18] * |
| **Mother had health insurance** |  |  |  |  |  |  |  |
| No | 5577 (97.8) | 63.4 [62.1-64.7] | 1.00 [reference] | 49.2 [47.9-50.5] | 1.00 [reference] | 52.1 [50.8-53.4] | 1.00 [reference] |
| Yes | 127 (2.2) | 84.3 [76.8-89.6] | 3.09 [1.91, 4.99] * | 71.7 [63.2-78.8] | 2.61 [1.77, 3.86] * | 78.0 [69.9-84.3] | 3.25 [2.13, 4.96] * |
| **Mother’s ethnicity** |  |  |  |  |  |  |  |
| Hausa/Fulani | 2284 (40.0) | 41.9 [39.9-43.9] | 1.00 [reference] | 27.7 [25.9-29.6] | 1.00 [reference] | 35.2 [33.3-37.2] | 1.00 [reference] |
| Igbo | 757 (13.3) | 92.3 [90.2-94.0] | 16.71 [12.62, 22.12] * | 81.6 [78.7-84.2] | 11.60 [9.44, 14.24] * | 75.3 [72.1-78.2] | 5.60 [4.65, 6.75] * |
| Others | 2072 (36.3) | 71.4 [69.4-73.3] | 3.47 [3.05, 3.93] * | 55.6 [53.4-57.7] | 3.27 [2.88, 3.70] * | 58.1 [55.9-60.2] | 2.54 [2.25, 2.87] * |
| Yoruba | 591 (10.4) | 85.8 [82.7-88.4] | 8.37 [6.55, 10.70] * | 72.9 [69.2-76.4] | 7.03 [5.73, 8.61] * | 72.1 [68.3-75.6] | 4.74 [3.89, 5.79] * |
| **Household wealth** |  |  |  |  |  |  |  |
| Poorer/poorest | 2669 (46.8) | 45.7 [43.8-47.6] | 1.00 [reference] | 30.9 [29.1-32.7] | 1.00 [reference] | 36.5 [34.7-38.3] | 1.00 [reference] |
| Middle | 1160 (20.3) | 69.4 [66.7-72.0] | 2.70 [2.33, 3.12] * | 54.0 [51.1-56.8] | 2.62 [2.28, 3.02] * | 55.3 [52.4-58.1] | 2.15 [1.87, 2.47] * |
| Richer/richest | 1875 (32.9) | 86.3 [84.7-87.8] | 7.52 [6.46, 8.76] * | 73.8 [71.8-75.8] | 6.31 [5.53, 7.20] * | 74.1 [72.0-76.0] | 4.97 [4.37, 5.66] * |
| **Mother owned a bank account** |  |  |  |  |  |  |  |
| No | 4755 (83.4) | 57.9 [56.5-59.3] | 1.00 [reference] | 43.2 [41.8-44.6] | 1.00 [reference] | 46.7 [45.3-48.1] | 1.00 [reference] |
| Yes | 949 (16.6) | 93.7 [91.9-95.1] | 10.77 [8.24, 14.07] * | 82.2 [79.6-84.5] | 6.07 [5.09, 7.24] * | 82.6 [80.1-84.9] | 5.43 [4.54, 6.48] * |
| **Household size** |  |  |  |  |  |  |  |
| Large (>=9) | 1382 (24.2) | 50.5 [47.9-53.1] | 1.00 [reference] | 37.3 [34.8-39.8] | 1.00 [reference] | 42.4 [39.8-45.0] | 1.00 [reference] |
| Medium (5-8) | 2580 (45.2) | 66.1 [64.2-67.9] | 1.91 [1.67, 2.18] * | 51.4 [49.5-53.4] | 1.78 [1.56, 2.04] * | 54.5 [52.6-56.4] | 1.63 [1.43, 1.86] * |
| Small (<=4) | 1742 (30.5) | 71.2 [69.0-73.3] | 2.42 [2.09, 2.81] * | 56.9 [54.6-59.3] | 2.23 [1.93, 2.57] * | 58.1 [55.8-60.4] | 1.88 [1.63, 2.17] * |
| **Length of stay in place of residence** |  |  |  |  |  |  |  |
| <1 year/visitor | 130 (2.3) | 83.1 [75.6-88.6] | 1.00 [reference] | 66.9 [58.4-74.5] | 1.00 [reference] | 63.1 [54.5-70.9] | 1.00 [reference] |
| 1-3 years | 657 (11.5) | 73.5 [70.0-76.8] | 0.57 [0.35, 0.92] * | 62.9 [59.1-66.5] | 0.84 [0.56, 1.25] | 62.4 [58.6-66.0] | 0.97 [0.66, 1.43] |
| 4-5 years | 415 (7.3) | 71.6 [67.0-75.7] | 0.51 [0.31, 0.85] * | 60.2 [55.5-64.8] | 0.75 [0.49, 1.13] | 58.1 [53.3-62.7] | 0.81 [0.54, 1.22] |
| >5 years/always | 4502 (78.9) | 61.2 [59.8-62.6] | 0.32 [0.20, 0.51] * | 46.3 [44.8-47.8] | 0.43 [0.29, 0.62] * | 50.4 [49.0-51.9] | 0.60 [0.42, 0.85] * |
| **Urban/rural** |  |  |  |  |  |  |  |
| Rural | 3741 (65.6) | 55.4 [53.8-57.0] | 1.00 [reference] | 40.2 [38.6-41.8] | 1.00 [reference] | 44.9 [43.3-46.5] | 1.00 [reference] |
| Urban | 1963 (34.4) | 80.0 [78.2-81.7] | 3.23 [2.84, 3.67] * | 67.8 [65.7-69.8] | 3.13 [2.79, 3.51] * | 67.4 [65.3-69.5] | 2.54 [2.27, 2.85] * |
| **Conflict area** |  |  |  |  |  |  |  |
| Yes | 1189 (20.8) | 70.7 [68.1-73.2] | 1.00 [reference] | 54.6 [51.7-57.4] | 1.00 [reference] | 55.8 [52.9-58.6] | 1.00 [reference] |
| No | 4515 (79.2) | 62.1 [60.6-63.5] | 0.68 [0.59, 0.78] * | 48.4 [46.9-49.9] | 0.78 [0.69, 0.89] * | 51.8 [50.4-53.3] | 0.85 [0.75, 0.97] * |
| **Urban slum** |  |  |  |  |  |  |  |
| Yes | 151 (2.6) | 57.6 [49.6-65.2] | 1.00 [reference] | 49.7 [41.8-57.6] | 1.00 [reference] | 49.7 [41.8-57.6] | 1.00 [reference] |
| No | 5553 (97.4) | 64.0 [62.8-65.3] | 1.31 [0.94, 1.82] | 49.7 [48.4-51.0] | 1.00 [0.72, 1.38] | 52.7 [51.4-54.1] | 1.13 [0.82, 1.56] |
| **Travel time (mins)** |  |  |  |  |  |  |  |
| Higher (32.2 – 592) | 1921 (33.7) | 49.4 [47.2-51.6] | 1.00 [reference] | 35.3 [33.2-37.5] | 1.00 [reference] | 40.9 [38.7-43.1] | 1.00 [reference] |
| Medium (9.87 - 32.1) | 1899 (33.3) | 61.2 [59.0-63.4] | 1.61 [1.42, 1.84] * | 45.0 [42.7-47.2] | 1.50 [1.32, 1.71] * | 48.5 [46.3-50.7] | 1.36 [1.20, 1.55] * |
| Lower (0 - 9.86) | 1884 (33.0) | 81.3 [79.5-83.0] | 4.46 [3.85, 5.16] * | 69.1 [67.0-71.2] | 4.10 [3.58, 4.70] * | 68.9 [66.8-70.9] | 3.21 [2.81, 3.66] * |

*Estimated crude odds ratio significant

**Table L: Bivariate analysis of factors associated with receipt of DPT1, DTP3 and MCV1 for Pakistan, 2017-18 DHS**

|  |  | **DTP1** |  | **DTP3** |  | **MCV1** |  |
| --- | --- | --- | --- | --- | --- | --- | --- |
| **Characteristics and categories** | **Number (%) in category** | **Percentage vaccinated**  **(95% CI)** | **cOR [95% CI]** | **Percentage vaccinated**  **(95% CI)** | **cOR [95% CI]** | **Percentage vaccinated**  **(95% CI)** | **cOR [95% CI]** |
| Total number of children | 2035 |  |  |  |  |  |  |
| **Sex of child** |  |  |  |  |  |  |  |
| Male | 1030 (50.6) | 82.8 [80.4-85.0] | 1.00 [reference] | 71.9 [69.1-74.6] | 1.00 [reference] | 68.4 [65.5-71.2] | 1.00 [reference] |
| Female | 1005 (49.4) | 80.3 [77.7-82.6] | 0.85 [0.68, 1.06] | 69.9 [66.9-72.6] | 0.90 [0.75, 1.09] | 66.7 [63.7-69.5] | 0.92 [0.77, 1.11] |
| **Birth order** |  |  |  |  |  |  |  |
| 1-2 | 906 (44.5) | 84.8 [82.3-87.0] | 1.00 [reference] | 74.3 [71.3-77.0] | 1.00 [reference] | 71.1 [68.0-73.9] | 1.00 [reference] |
| 3-5 | 808 (39.7) | 82.2 [79.4-84.7] | 0.83 [0.64, 1.07] | 72.2 [69.0-75.1] | 0.90 [0.72, 1.11] | 69.1 [65.8-72.2] | 0.91 [0.74, 1.12] |
| >5 | 321 (15.8) | 71.0 [65.8-75.7] | 0.44 [0.33, 0.60] * | 58.3 [52.8-63.5] | 0.48 [0.37, 0.63] * | 53.9 [48.4-59.3] | 0.48 [0.37, 0.62] * |
| **Skilled birth attendance (SBA)** |  |  |  |  |  |  |  |
| No | 633 (31.1) | 65.6 [61.8-69.2] | 1.00 [reference] | 51.7 [47.8-55.5] | 1.00 [reference] | 49.0 [45.1-52.9] | 1.00 [reference] |
| Yes | 1402 (68.9) | 88.8 [87.0-90.4] | 4.17 [3.30, 5.26] * | 79.6 [77.4-81.6] | 3.65 [2.98, 4.47] * | 76.0 [73.7-78.1] | 3.29 [2.70, 4.01] * |
| **Antenatal care attendance (ANC)** |  |  |  |  |  |  |  |
| No ANC | 312 (15.3) | 52.9 [47.3-58.4] | 1.00 [reference] | 42.0 [36.6-47.5] | 1.00 [reference] | 39.1 [33.8-44.6] | 1.00 [reference] |
| 1-3 | 704 (34.6) | 80.8 [77.7-83.6] | 3.76 [2.81, 5.02] * | 68.3 [64.8-71.7] | 2.98 [2.26, 3.92] * | 64.5 [60.9-67.9] | 2.83 [2.15, 3.72] * |
| >=4 | 1019 (50.1) | 90.9 [88.9-92.5] | 8.87 [6.52, 12.07] * | 81.6 [79.0-83.8] | 6.11 [4.64, 8.04] * | 78.4 [75.8-80.8] | 5.66 [4.31, 7.42] * |
| **Birth quarter** |  |  |  |  |  |  |  |
| Jan-Mar | 468 (23.0) | 79.7 [75.8-83.1] | 1.00 [reference] | 68.6 [64.2-72.6] | 1.00 [reference] | 65.2 [60.7-69.4] | 1.00 [reference] |
| Apr-Jun | 409 (20.1) | 82.4 [78.4-85.8] | 1.19 [0.85, 1.67] | 70.7 [66.1-74.9] | 1.10 [0.83, 1.47] | 68.2 [63.5-72.6] | 1.15 [0.87, 1.52] |
| Jul-Sep | 569 (28.0) | 83.3 [80.0-86.1] | 1.27 [0.93, 1.74] | 72.1 [68.2-75.6] | 1.18 [0.90, 1.54] | 68.9 [65.0-72.6] | 1.18 [0.91, 1.54] |
| Oct-Dec | 589 (28.9) | 80.8 [77.4-83.8] | 1.07 [0.79, 1.45] | 71.8 [68.0-75.3] | 1.17 [0.90, 1.52] | 67.7 [63.9-71.4] | 1.12 [0.87, 1.45] |
| **Mother’s TT vaccinations before birth** |  |  |  |  |  |  |  |
| 0 | 683 (33.6) | 57.5 [53.8-61.2] | 1.00 [reference] | 45.1 [41.4-48.8] | 1.00 [reference] | 42.3 [38.7-46.1] | 1.00 [reference] |
| 1-2 | 944 (46.4) | 93.9 [92.1-95.2] | 11.27 [8.30, 15.31] * | 83.5 [81.0-85.7] | 6.15 [4.89, 7.73] * | 81.0 [78.4-83.4] | 5.83 [4.66, 7.28] * |
| 3-4 | 363 (17.8) | 94.8 [91.9-96.6] | 13.36 [8.22, 21.72] * | 86.0 [82.0-89.2] | 7.45 [5.34, 10.38] * | 80.2 [75.7-84.0] | 5.51 [4.08, 7.43] * |
| >=5 | 45 (2.2) | 82.2 [68.3-90.9] | 3.41 [1.57, 7.44] * | 77.8 [63.4-87.6] | 4.26 [2.08, 8.74] * | 66.7 [51.8-78.8] | 2.73 [1.44, 5.16] * |
| **Post-natal care (PNC)** |  |  |  |  |  |  |  |
| No/don't know | 1488 (73.1) | 79.3 [77.2-81.3] | 1.00 [reference] | 68.9 [66.5-71.2] | 1.00 [reference] | 64.5 [62.0-66.9] | 1.00 [reference] |
| Yes | 547 (26.9) | 87.8 [84.7-90.2] | 1.87 [1.41, 2.49] * | 76.4 [72.7-79.8] | 1.46 [1.17, 1.83] * | 75.9 [72.1-79.3] | 1.73 [1.38, 2.16] * |
| **Sex of head of household** |  |  |  |  |  |  |  |
| Female | 214 (10.5) | 89.7 [84.9-93.1] | 1.00 [reference] | 81.8 [76.0-86.4] | 1.00 [reference] | 76.6 [70.5-81.8] | 1.00 [reference] |
| Male | 1821 (89.5) | 80.6 [78.7-82.4] | 0.48 [0.30, 0.75] * | 69.6 [67.5-71.7] | 0.51 [0.36, 0.73] * | 66.5 [64.3-68.6] | 0.61 [0.43, 0.84] * |
| **Maternal age (years)** |  |  |  |  |  |  |  |
| 15-19 | 86 (4.2) | 70.9 [60.5-79.5] | 1.00 [reference] | 57.0 [46.4-67.0] | 1.00 [reference] | 53.5 [42.9-63.7] | 1.00 [reference] |
| 20-29 | 1100 (54.1) | 82.2 [79.8-84.3] | 1.89 [1.16, 3.09] * | 71.5 [68.7-74.0] | 1.89 [1.21, 2.95] * | 68.6 [65.8-71.3] | 1.90 [1.22, 2.96] * |
| 30-39 | 776 (38.1) | 82.0 [79.1-84.5] | 1.86 [1.13, 3.07] * | 72.2 [68.9-75.2] | 1.96 [1.24, 3.09] * | 68.0 [64.7-71.2] | 1.85 [1.18, 2.90] * |
| 40-49 | 73 (3.6) | 80.8 [70.2-88.3] | 1.73 [0.82, 3.64] | 65.8 [54.2-75.7] | 1.45 [0.76, 2.76] | 63.0 [51.4-73.3] | 1.48 [0.78, 2.80] |
| **Mother’s employment status** |  |  |  |  |  |  |  |
| No | 1790 (88.0) | 81.7 [79.8-83.4] | 1.00 [reference] | 71.0 [68.9-73.1] | 1.00 [reference] | 67.5 [65.3-69.6] | 1.00 [reference] |
| Yes (currently/in the past 1 year) | 245 (12.0) | 80.8 [75.4-85.3] | 0.95 [0.67, 1.33] | 70.2 [64.2-75.6] | 0.96 [0.72, 1.29] | 68.2 [62.1-73.7] | 1.03 [0.77, 1.37] |
| **Mother had problem seeking medical advice or treatment** |  |  |  |  |  |  |  |
| Had problem | 556 (27.3) | 92.3 [89.7-94.2] | 1.00 [reference] | 82.9 [79.6-85.8] | 1.00 [reference] | 78.4 [74.8-81.6] | 1.00 [reference] |
| Had no problem | 1479 (72.7) | 77.6 [75.4-79.6] | 0.29 [0.21, 0.40] * | 66.4 [63.9-68.8] | 0.41 [0.32, 0.52] * | 63.5 [61.0-65.9] | 0.48 [0.38, 0.60] * |
| **Maternal education** |  |  |  |  |  |  |  |
| None | 978 (48.1) | 67.4 [64.4-70.3] | 1.00 [reference] | 53.3 [50.1-56.4] | 1.00 [reference] | 50.0 [46.9-53.1] | 1.00 [reference] |
| Primary | 257 (12.6) | 92.2 [88.2-94.9] | 5.74 [3.57, 9.23] * | 82.5 [77.3-86.7] | 4.13 [2.93, 5.84] * | 79.8 [74.4-84.2] | 3.94 [2.84, 5.48] * |
| Sec./higher | 800 (39.3) | 95.5 [93.8-96.7] | 10.27 [7.17, 14.72] * | 88.8 [86.4-90.8] | 6.92 [5.37, 8.91] * | 85.1 [82.5-87.4] | 5.72 [4.54, 7.21] * |
| **Mother’s media exposure** |  |  |  |  |  |  |  |
| No | 1056 (51.9) | 72.7 [70.0-75.3] | 1.00 [reference] | 60.9 [57.9-63.8] | 1.00 [reference] | 58.0 [55.0-60.9] | 1.00 [reference] |
| Yes | 979 (48.1) | 91.1 [89.2-92.7] | 3.84 [2.97, 4.98] * | 81.7 [79.2-84.0] | 2.87 [2.34, 3.52] * | 77.9 [75.2-80.4] | 2.56 [2.11, 3.11] * |
| **Mother’s use of mobile phone** |  |  |  |  |  |  |  |
| No | 1159 (57.0) | 72.7 [70.1-75.2] | 1.00 [reference] | 60.9 [58.1-63.7] | 1.00 [reference] | 57.9 [55.0-60.7] | 1.00 [reference] |
| Yes | 876 (43.0) | 93.3 [91.4-94.7] | 5.19 [3.87, 6.97] * | 84.1 [81.6-86.4] | 3.40 [2.74, 4.22] * | 80.4 [77.6-82.9] | 2.98 [2.43, 3.65] * |
| **Mother’s land ownership** |  |  |  |  |  |  |  |
| Does not own land | 2006 (98.6) | 81.5 [79.7-83.1] | 1.00 [reference] | 70.7 [68.7-72.7] | 1.00 [reference] | 67.4 [65.3-69.4] | 1.00 [reference] |
| Alone or jointly or both | 29 (1.4) | 86.2 [68.5-94.7] | 1.42 [0.49, 4.10] | 82.8 [64.7-92.6] | 1.99 [0.75, 5.23] | 79.3 [60.9-90.4] | 1.85 [0.75, 4.58] |
| **Mother had health insurance** |  |  |  |  |  |  |  |
| No | 2003 (98.4) | 81.4 [79.7-83.1] | 1.00 [reference] | 70.8 [68.8-72.7] | 1.00 [reference] | 67.5 [65.5-69.6] | 1.00 [reference] |
| Yes | 32 (1.6) | 90.6 [74.6-96.9] | 2.20 [0.67, 7.28] | 78.1 [60.7-89.2] | 1.47 [0.63, 3.43] | 68.8 [51.0-82.3] | 1.06 [0.50, 2.24] |
| **Household owned a mosquito bednet** |  |  |  |  |  |  |  |
| No | 1537 (75.5) | 80.6 [78.6-82.5] | 1.00 [reference] | 70.1 [67.8-72.4] | 1.00 [reference] | 66.3 [63.9-68.6] | 1.00 [reference] |
| Yes | 498 (24.5) | 84.5 [81.1-87.5] | 1.32 [1.00, 1.73] | 73.3 [69.2-77.0] | 1.17 [0.93, 1.47] | 71.5 [67.4-75.3] | 1.27 [1.02, 1.59] * |
| **Household wealth** |  |  |  |  |  |  |  |
| Poorer/poorest | 874 (42.9) | 69.2 [66.1-72.2] | 1.00 [reference] | 57.2 [53.9-60.5] | 1.00 [reference] | 54.2 [50.9-57.5] | 1.00 [reference] |
| Middle | 435 (21.4) | 86.4 [82.9-89.3] | 2.83 [2.08, 3.86] * | 73.3 [69.0-77.3] | 2.06 [1.60, 2.64] * | 71.5 [67.1-75.5] | 2.12 [1.65, 2.71] * |
| Richer/richest | 726 (35.7) | 93.5 [91.5-95.1] | 6.42 [4.62, 8.92] * | 86.0 [83.2-88.3] | 4.58 [3.57, 5.87] * | 81.3 [78.3-83.9] | 3.66 [2.91, 4.60] * |
| **Mother owned a bank account** |  |  |  |  |  |  |  |
| No | 1932 (94.9) | 80.9 [79.1-82.6] | 1.00 [reference] | 69.9 [67.8-71.9] | 1.00 [reference] | 66.5 [64.3-68.5] | 1.00 [reference] |
| Yes | 103 (5.1) | 94.2 [87.6-97.4] | 3.82 [1.66, 8.77] * | 90.3 [82.9-94.7] | 4.01 [2.07, 7.75] * | 88.3 [80.6-93.3] | 3.83 [2.08, 7.04] * |
| **Household size** |  |  |  |  |  |  |  |
| Large (>=9) | 960 (47.2) | 78.1 [75.4-80.6] | 1.00 [reference] | 66.7 [63.6-69.6] | 1.00 [reference] | 63.1 [60.0-66.1] | 1.00 [reference] |
| Medium (5-8) | 871 (42.8) | 84.4 [81.8-86.6] | 1.51 [1.19, 1.92] * | 75.7 [72.7-78.4] | 1.55 [1.27, 1.91] * | 72.8 [69.7-75.6] | 1.56 [1.28, 1.91] * |
| Small (<=4) | 204 (10.0) | 85.8 [80.3-89.9] | 1.69 [1.11, 2.58] * | 70.6 [64.0-76.4] | 1.20 [0.86, 1.67] | 66.2 [59.4-72.3] | 1.14 [0.83, 1.57] |
| **Urban/rural** |  |  |  |  |  |  |  |
| Rural | 1128 (55.4) | 75.6 [73.0-78.0] | 1.00 [reference] | 64.9 [62.1-67.6] | 1.00 [reference] | 62.9 [60.1-65.7] | 1.00 [reference] |
| Urban | 907 (44.6) | 89.0 [86.8-90.9] | 2.60 [2.03, 3.33] * | 78.4 [75.6-81.0] | 1.96 [1.61, 2.40] * | 73.3 [70.3-76.1] | 1.62 [1.34, 1.96] * |
| **Conflict area** |  |  |  |  |  |  |  |
| Yes | 351 (17.2) | 51.3 [46.1-56.5] | 1.00 [reference] | 39.9 [34.9-45.1] | 1.00 [reference] | 35.6 [30.8-40.8] | 1.00 [reference] |
| No | 1684 (82.8) | 87.9 [86.2-89.4] | 6.89 [5.34, 8.90] * | 77.4 [75.3-79.3] | 5.15 [4.05, 6.57] * | 74.2 [72.1-76.3] | 5.21 [4.08, 6.65] * |
| **Urban slum** |  |  |  |  |  |  |  |
| Yes | 105 (5.2) | 76.2 [67.1-83.4] | 1.00 [reference] | 61.9 [52.3-70.7] | 1.00 [reference] | 58.1 [48.5-67.1] | 1.00 [reference] |
| No | 1930 (94.8) | 81.9 [80.1-83.5] | 1.41 [0.89, 2.24] | 71.4 [69.3-73.4] | 1.54 [1.02, 2.31] * | 68.1 [66.0-70.1] | 1.54 [1.03, 2.29] * |
| **Travel time (mins)** |  |  |  |  |  |  |  |
| Higher (35.3 – 827) | 694 (34.1) | 67.1 [63.6-70.5] | 1.00 [reference] | 59.1 [55.4-62.7] | 1.00 [reference] | 56.9 [53.2-60.6] | 1.00 [reference] |
| Medium (5.14 - 35.2) | 670 (32.9) | 86.4 [83.6-88.8] | 3.11 [2.37, 4.09] * | 74.0 [70.6-77.2] | 1.97 [1.57, 2.48] * | 70.7 [67.2-74.1] | 1.83 [1.46, 2.29] * |
| Lower (0 - 5.13) | 671 (33.0) | 91.7 [89.3-93.5] | 5.37 [3.92, 7.37] * | 80.0 [76.8-82.9] | 2.78 [2.18, 3.54] * | 75.4 [72.0-78.5] | 2.32 [1.84, 2.93] * |

*Estimated crude odds ratio significant

**Table M: Bivariate analysis of factors associated with receipt of DTP3 and MCV1 for Zambia, 2018 DHS**

|  |  | **DTP3** |  | **MCV1** |  |
| --- | --- | --- | --- | --- | --- |
| **Characteristics and categories** | **Number (%) in category** | **Percentage vaccinated**  **(95% CI)** | **cOR [95% CI]** | **Percentage vaccinated**  **(95% CI)** | **cOR [95% CI]** |
| Total number of children | 1818 |  |  |  |  |
| **Sex of child** |  |  |  |  |  |
| Male | 905 (49.8) | 90.7 [88.6-92.4] | 1.00 [reference] | 90.3 [88.2-92.0] | 1.00 [reference] |
| Female | 913 (50.2) | 93.3 [91.5-94.8] | 1.43 [1.01, 2.01] * | 91.0 [89.0-92.7] | 1.09 [0.80, 1.50] |
| **Birth order** |  |  |  |  |  |
| 1-2 | 789 (43.4) | 93.0 [91.0-94.6] | 1.00 [reference] | 91.9 [89.8-93.6] | 1.00 [reference] |
| 3-5 | 655 (36.0) | 92.4 [90.1-94.2] | 0.91 [0.61, 1.35] | 90.7 [88.2-92.7] | 0.86 [0.60, 1.24] |
| >5 | 374 (20.6) | 89.3 [85.7-92.1] | 0.63 [0.41, 0.96] * | 88.0 [84.3-90.9] | 0.65 [0.43, 0.97] * |
| **Skilled birth attendance (SBA)** |  |  |  |  |  |
| No | 347 (19.1) | 82.7 [78.4-86.3] | 1.00 [reference] | 81.3 [76.8-85.0] | 1.00 [reference] |
| Yes | 1471 (80.9) | 94.2 [92.9-95.3] | 3.41 [2.39, 4.86] * | 92.9 [91.4-94.1] | 3.00 [2.15, 4.19] * |
| **Antenatal care attendance (ANC)** |  |  |  |  |  |
| >=4 | 1201 (66.1) | 93.7 [92.1-94.9] | 1.00 [reference] | 91.4 [89.7-92.9] | 1.00 [reference] |
| 1-3 | 594 (32.7) | 90.2 [87.6-92.4] | 0.62 [0.44, 0.89] * | 90.4 [87.8-92.5] | 0.88 [0.63, 1.24] |
| No ANC | 23 (1.3) | 52.2 [32.5-71.2] | 0.07 [0.03, 0.17] * | 56.5 [36.3-74.8] | 0.12 [0.05, 0.28] * |
| **Post-natal care** **(PNC)** |  |  |  |  |  |
| No/don't know | 669 (36.8) | 87.6 [84.9-89.9] | 1.00 [reference] | 86.5 [83.7-88.9] | 1.00 [reference] |
| Yes | 1149 (63.2) | 94.6 [93.1-95.8] | 2.48 [1.76, 3.50] * | 93.0 [91.4-94.4] | 2.08 [1.51, 2.85] * |
| **Birth quarter** |  |  |  |  |  |
| Jan-Mar | 474 (26.1) | 91.1 [88.2-93.4] | 1.00 [reference] | 88.6 [85.4-91.2] | 1.00 [reference] |
| Apr-Jun | 425 (23.4) | 92.5 [89.5-94.6] | 1.19 [0.74, 1.93] | 90.6 [87.4-93.0] | 1.24 [0.80, 1.91] |
| Jul-Sep | 453 (24.9) | 92.1 [89.2-94.2] | 1.13 [0.71, 1.79] | 92.5 [89.7-94.6] | 1.58 [1.01, 2.48] * |
| Oct-Dec | 466 (25.6) | 92.5 [89.7-94.6] | 1.20 [0.75, 1.91] | 91.0 [88.0-93.3] | 1.30 [0.85, 1.99] |
| **Mother’s TT vaccinations before birth** |  |  |  |  |  |
| 0 | 382 (21.0) | 86.9 [83.1-89.9] | 1.00 [reference] | 86.6 [82.9-89.7] | 1.00 [reference] |
| 1-2 | 1315 (72.3) | 93.7 [92.2-94.9] | 2.24 [1.54, 3.24] * | 91.8 [90.2-93.2] | 1.72 [1.21, 2.45] * |
| >=3 | 121 (6.7) | 90.1 [83.3-94.3] | 1.37 [0.70, 2.66] | 90.9 [84.3-94.9] | 1.54 [0.78, 3.06] |
| **Sex of head of household** |  |  |  |  |  |
| Female | 361 (19.9) | 91.1 [87.7-93.7] | 1.00 [reference] | 88.9 [85.2-91.8] | 1.00 [reference] |
| Male | 1457 (80.1) | 92.2 [90.8-93.5] | 1.16 [0.77, 1.74] | 91.1 [89.5-92.4] | 1.27 [0.87, 1.85] |
| **Maternal age (years)** |  |  |  |  |  |
| 15-19 | 197 (10.8) | 90.4 [85.4-93.8] | 1.00 [reference] | 88.3 [83.0-92.1] | 1.00 [reference] |
| 20-29 | 922 (50.7) | 92.7 [90.9-94.2] | 1.36 [0.80, 2.32] | 91.8 [89.8-93.4] | 1.47 [0.90, 2.41] |
| 30-49 | 699 (38.5) | 91.6 [89.3-93.4] | 1.15 [0.67, 1.99] | 89.8 [87.3-91.9] | 1.17 [0.71, 1.93] |
| **Mother’s marital status** |  |  |  |  |  |
| Never in union | 223 (12.3) | 94.2 [90.2-96.6] | 1.00 [reference] | 93.3 [89.1-95.9] | 1.00 [reference] |
| Divorced/widowed/separated | 177 (9.7) | 90.4 [85.1-93.9] | 0.58 [0.27, 1.23] | 88.1 [82.5-92.1] | 0.54 [0.27, 1.07] |
| Married/with partner | 1418 (78.0) | 91.9 [90.3-93.2] | 0.70 [0.39, 1.27] | 90.6 [88.9-92.0] | 0.69 [0.40, 1.20] |
| **Mother had problem seeking medical advice or treatment** |  |  |  |  |  |
| Had problem | 1011 (55.6) | 94.1 [92.4-95.4] | 1.00 [reference] | 92.3 [90.5-93.8] | 1.00 [reference] |
| Had no problem | 807 (44.4) | 89.5 [87.2-91.4] | 0.54 [0.38, 0.76] * | 88.6 [86.2-90.6] | 0.65 [0.47, 0.89] * |
| **Maternal education** |  |  |  |  |  |
| None | 187 (10.3) | 82.9 [76.8-87.6] | 1.00 [reference] | 77.5 [71.0-83.0] | 1.00 [reference] |
| Primary | 957 (52.6) | 91.0 [89.0-92.7] | 2.09 [1.35, 3.25] * | 90.3 [88.2-92.0] | 2.69 [1.80, 4.03] * |
| Sec./higher | 674 (37.1) | 96.0 [94.2-97.2] | 4.95 [2.88, 8.50] * | 94.8 [92.9-96.2] | 5.29 [3.26, 8.58] * |
| **Mother’s media exposure** |  |  |  |  |  |
| No | 1035 (56.9) | 89.6 [87.5-91.3] | 1.00 [reference] | 88.7 [86.6-90.5] | 1.00 [reference] |
| Yes | 783 (43.1) | 95.3 [93.5-96.6] | 2.35 [1.60, 3.45] * | 93.2 [91.2-94.8] | 1.76 [1.25, 2.46] * |
| **Mother’s use of mobile phone** |  |  |  |  |  |
| No | 1076 (59.2) | 89.9 [87.9-91.5] | 1.00 [reference] | 88.6 [86.5-90.3] | 1.00 [reference] |
| Yes | 742 (40.8) | 95.1 [93.3-96.5] | 2.21 [1.50, 3.26] * | 93.7 [91.7-95.2] | 1.91 [1.34, 2.71] * |
| **Mother’s land ownership** |  |  |  |  |  |
| Does not own land | 1112 (61.2) | 93.1 [91.4-94.4] | 1.00 [reference] | 91.7 [90.0-93.2] | 1.00 [reference] |
| Alone or jointly or both | 706 (38.8) | 90.4 [88.0-92.3] | 0.70 [0.50, 0.98] * | 89.0 [86.4-91.1] | 0.73 [0.53, 1.00] * |
| **Mother’s employment status** |  |  |  |  |  |
| No | 783 (43.1) | 91.7 [89.5-93.4] | 1.00 [reference] | 91.1 [88.8-92.9] | 1.00 [reference] |
| Yes (currently/in the past 1 year) | 1035 (56.9) | 92.3 [90.5-93.7] | 1.08 [0.77, 1.52] | 90.3 [88.4-92.0] | 0.92 [0.67, 1.26] |
| **Mother had health insurance** |  |  |  |  |  |
| No | 1784 (98.1) | 91.9 [90.6-93.1] | 1.00 [reference] | 90.6 [89.1-91.9] | 1.00 [reference] |
| Yes | 34 (1.9) | 97.1 [81.8-99.6] | 2.90 [0.39, 21.34] | 94.1 [79.3-98.5] | 1.66 [0.40, 7.00] |
| **Household owned a mosquito bednet** |  |  |  |  |  |
| No | 282 (15.5) | 89.4 [85.2-92.5] | 1.00 [reference] | 83.0 [78.1-86.9] | 1.00 [reference] |
| Yes | 1536 (84.5) | 92.5 [91.1-93.7] | 1.47 [0.96, 2.25] | 92.1 [90.6-93.3] | 2.38 [1.66, 3.41] * |
| **Household wealth** |  |  |  |  |  |
| Poorer/poorest | 956 (52.6) | 89.9 [87.8-91.6] | 1.00 [reference] | 88.8 [86.6-90.7] | 1.00 [reference] |
| Middle | 358 (19.7) | 92.7 [89.5-95.0] | 1.44 [0.92, 2.26] | 91.3 [87.9-93.8] | 1.33 [0.87, 2.02] |
| Richer/richest | 504 (27.7) | 95.6 [93.5-97.1] | 2.47 [1.54, 3.98] * | 93.7 [91.2-95.5] | 1.86 [1.23, 2.80] * |
| **Mother owned a bank account** |  |  |  |  |  |
| No | 1676 (92.2) | 91.6 [90.2-92.8] | 1.00 [reference] | 90.2 [88.7-91.5] | 1.00 [reference] |
| Yes | 142 (7.8) | 97.2 [92.7-98.9] | 3.17 [1.16, 8.69] * | 95.8 [90.9-98.1] | 2.46 [1.07, 5.66] * |
| **Household size** |  |  |  |  |  |
| Large (>=9) | 335 (18.4) | 90.7 [87.1-93.4] | 1.00 [reference] | 91.3 [87.8-93.9] | 1.00 [reference] |
| Medium (5-8) | 978 (53.8) | 93.4 [91.6-94.8] | 1.43 [0.92, 2.24] | 90.9 [88.9-92.5] | 0.95 [0.61, 1.47] |
| Small (<=4) | 505 (27.8) | 90.3 [87.4-92.6] | 0.95 [0.59, 1.52] | 89.7 [86.7-92.1] | 0.83 [0.51, 1.33] |
| **Length of stay in place of residence** |  |  |  |  |  |
| >5 years /always | 1112 (61.2) | 91.5 [89.7-93.0] | 1.00 [reference] | 89.8 [87.9-91.5] | 1.00 [reference] |
| <1 year /visitor | 166 (9.1) | 93.4 [88.4-96.3] | 1.32 [0.69, 2.51] | 86.1 [80.0-90.6] | 0.70 [0.43, 1.14] |
| 1-5 years | 540 (29.7) | 92.8 [90.3-94.7] | 1.20 [0.81, 1.77] | 97.5 [93.6-99.1] | 1.68 [1.13, 2.51] * |
| **Urban/rural** |  |  |  |  |  |
| Rural | 1256 (69.1) | 90.8 [89.1-92.3] | 1.00 [reference] | 90.1 [88.3-91.7] | 1.00 [reference] |
| Urban | 562 (30.9) | 94.7 [92.5-96.2] | 1.79 [1.18, 2.71] * | 91.8 [89.2-93.8] | 1.23 [0.86, 1.75] |
| **Region** |  |  |  |  |  |
| West./South./North W. | 509 (28.0) | 90.0 [87.1-92.3] | 1.00 [reference] | 90.2 [87.3-92.5] | 1.00 [reference] |
| Cent./Cop./East./Lus. | 759 (41.7) | 94.3 [92.4-95.8] | 1.85 [1.22, 2.83] * | 92.2 [90.1-93.9] | 1.29 [0.87, 1.92] |
| North./Much./Luap. | 550 (30.3) | 90.7 [88.0-92.9] | 1.09 [0.72, 1.64] | 88.9 [86.0-91.3] | 0.87 [0.59, 1.30] |
| **Urban slum** |  |  |  |  |  |
| Yes | 37 (2.0) | 94.6 [80.8-98.6] | 1.00 [reference] | 91.9 [77.7-97.4] | 1.00 [reference] |
| No | 1781 (98.0) | 92.0 [90.6-93.1] | 0.65 [0.16, 2.75] | 90.6 [89.2-91.9] | 0.85 [0.26, 2.81] |
| **Travel time (mins)** |  |  |  |  |  |
| Higher (106.1 – 756) | 595 (32.7) | 87.7 [84.8-90.1] | 1.00 [reference] | 88.2 [85.4-90.6] | 1.00 [reference] |
| Medium (27.7 – 106) | 614 (33.8) | 94.0 [91.8-95.6] | 2.18 [1.44, 3.30] * | 91.2 [88.7-93.2] | 1.38 [0.95, 2.01] |
| Lower (0 - 27.6) | 609 (33.5) | 94.3 [92.1-95.8] | 2.29 [1.51, 3.49] * | 92.4 [90.1-94.3] | 1.63 [1.10, 2.41] * |

*Estimated crude odds ratio significant

**Table N: Bivariate analysis comparing the likelihood of vaccination in formal urban areas to urban slums and rural areas**

| **Place of residence** | **Number (%) in category** | **DTP1** | **DTP3** | **MCV1** |
| --- | --- | --- | --- | --- |
|  |  | **cOR [95% CI]** | **cOR [95% CI]** | **cOR [95% CI]** |
|  | **DRC** | | | |
| Formal urban | 527 (17.9) | 1.00 [reference] | 1.00 [reference] | 1.00 [reference] |
| Urban slum | 418 (14.2) | 0.30 [0.19 – 0.48]* | 0.38 [0.28 – 0.50]* | 0.49 [0.36 – 0.67]* |
| Rural | 2003 (67.9) | 0.15 [0.10 – 0.22]* | 0.26 [0.21 – 0.33]* | 0.38 [0.30 – 0.49]* |
|  | **Nigeria** | | | |
| Formal urban | 1812 (31.8) | 1.00 [reference] | 1.00 [reference] | 1.00 [reference] |
| Urban slum | 151 (2.6) | 0.30 [0.21 – 0.42]* | 0.44 [0.31 – 0.61]* | 0.44 [0.32 – 0.62]* |
| Rural | 3741 (65.6) | 0.27 [0.24 – 0.31]* | 0.30 [0.26 – 0.34]* | 0.37 [0.33 – 0.41]* |
|  | **Ethiopia** | | | |
| Formal urban | 315 (17.9) | 1.00 [reference] | 1.00 [reference] | 1.00 [reference] |
| Urban slum | 68 (3.9) | 0.17 [0.07 – 0.37]* | 0.17 [0.09 – 0.30]* | 0.22 [0.13 – 0.39]* |
| Rural | 1374 (78.2) | 0.09 [0.05 – 0.15]* | 0.14 [0.10 – 0.20]* | 0.19 [0.13 – 0.26]* |
|  | **Madagascar** | | | |
| Formal urban | 284 (13.9) | 1.00 [reference] | 1.00 [reference] | 1.00 [reference] |
| Urban slum | 70 (3.4) | 0.59 [0.23 – 1.72] | 0.56 [0.28 – 1.16] | 0.50 [0.26 – 1.00] |
| Rural | 1685 (82.7) | 0.23 [0.13 – 0.37]* | 0.26 [0.18 – 0.38]* | 0.23 [0.16 – 0.34]* |
|  | **Mozambique** | | | |
| Formal urban | 533 (25.2) | 1.00 [reference] | 1.00 [reference] | 1.00 [reference] |
| Urban slum | 128 (6.1) | 0.71 [0.29 – 1.99] | 1.29 [0.71 – 2.52] | 0.51 [0.28 – 0.97]* |
| Rural | 1449 (68.7) | 0.32 [0.19 – 0.52]* | 0.46 [0.35 – 0.61]* | 0.28 [0.19 – 0.40]* |
|  | **Pakistan** | | | |
| Formal urban | 802 (39.4) | 1.00 [reference] | 1.00 [reference] | 1.00 [reference] |
| Urban slum | 105 (5.2) | 0.33 [0.20 – 0.56]* | 0.39 [0.26 – 0.61]* | 0.45 [0.30 – 0.69]* |
| Rural | 1128 (55.4) | 0.32 [0.24 – 0.42]* | 0.45 [0.36 – 0.55]* | 0.56 [0.45 – 0.68]* |
|  | **India** | | | |
| Formal urban | 10765 (23.3) | 1.00 [reference] | 1.00 [reference] | 1.00 [reference] |
| Urban slum | 360 (0.8) | 0.68 [0.50 – 0.94]* | 0.60 [0.48 – 0.76]* | 0.63 [0.50 – 0.82]* |
| Rural | 35005 (75.9) | 0.76 [0.70 – 0.81]* | 0.79 [0.75 – 0.83]* | 0.79 [0.75 – 0.83]* |
|  | **Zambia** | | | |
| Formal urban | 525 (28.9) | NA | 1.00 [reference] | 1.00 [reference] |
| Urban slum | 37 (2.0) | NA | 0.99 [0.28 – 6.26] | 1.01 [0.34 – 4.32] |
| Rural | 1256 (69.1) | NA | 0.56 [0.36 – 0.84]* | 0.81 [0.56 – 1.16] |

* Estimated crude odds ratio significant

Note: Cambodia was excluded from this analysis as no slum area was found in the data used.

Also, for Zambia, DTP1 was not included in the analysis due to small numbers of unvaccinated children.

**Table O: Bivariate analysis results for conflict based on the “narrow” definition (i.e. areas that had > 300 deaths per 1 million**

**population due to conflict). Note that this only relates to countries where conflict-affected areas were identified according to this**

**definition as shown in Table 4.**

| **Country** | **Number (%) in category** | **DTP1**  **cOR [95% CI]** | **DTP3**  **cOR [95% CI]** | **MCV1**  **cOR [95% CI]** |
| --- | --- | --- | --- | --- |
| **DRC** | | | | |
| Conflict area |  |  |  |  |
| Yes | 45 (1.5) | 1.00 [reference] | 1.00 [reference] | 1.00 [reference] |
| No | 2903 (98.5) | 0.24 [0.06, 0.68]* | 0.18 [0.07, 0.42] * | 0.27 [0.09, 0.63] * |
| **Mozambique** | | | | |
| Conflict area |  |  |  |  |
| Yes | 13 (0.6) | 1.00 [reference] | 1.00 [reference] | 1.00 [reference] |
| No | 2097 (99.4) | 0.98 [0.05, 5.00] | 1.75 [0.47, 5.40] | 0.42 [0.02, 2.14] |
| **Nigeria** | | | | |
| Conflict area |  |  |  |  |
| Yes | 313 (5.5) | 1.00 [reference] | 1.00 [reference] | 1.00 [reference] |
| No | 5391 (94.5) | 1.44 [1.14, 1.81] * | 1.75 [1.39, 2.22] * | 1.42 [1.13, 1.79] * |
| **India** | | | | |
| Conflict area |  |  |  |  |
| Yes | 300 (0.7) | 1.00 [reference] | 1.00 [reference] | 1.00 [reference] |
| No | 45830 (99.3) | 0.18 [0.08, 0.36] * | 0.36 [0.24, 0.52] * | 0.37 [0.25, 0.55] * |

*Estimated crude odds ratio significant

**Multivariate analysis results – figures and detailed interpretation of estimated relationships**

**
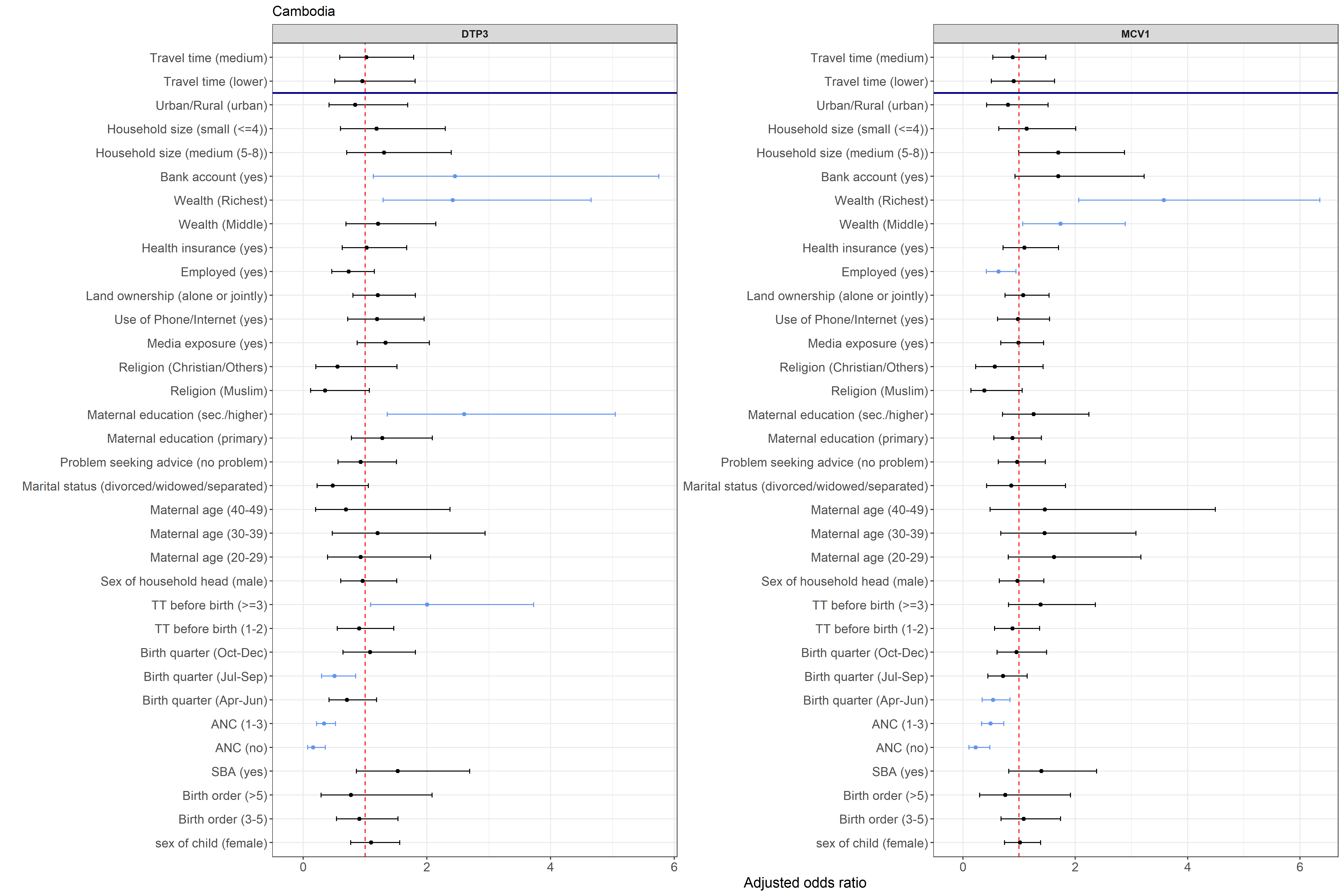
**

**Fig H: Adjusted odds ratio (aOR) and corresponding 95% credible interval (95% CI) plots for Cambodia. The vertical dotted red lines mark the odds ratio of 1. Light blue dots and lines show the aORs and 95CIs of variables that have significant associations with vaccination. A dark blue horizontal line separates the key community variables from other covariates.**

**
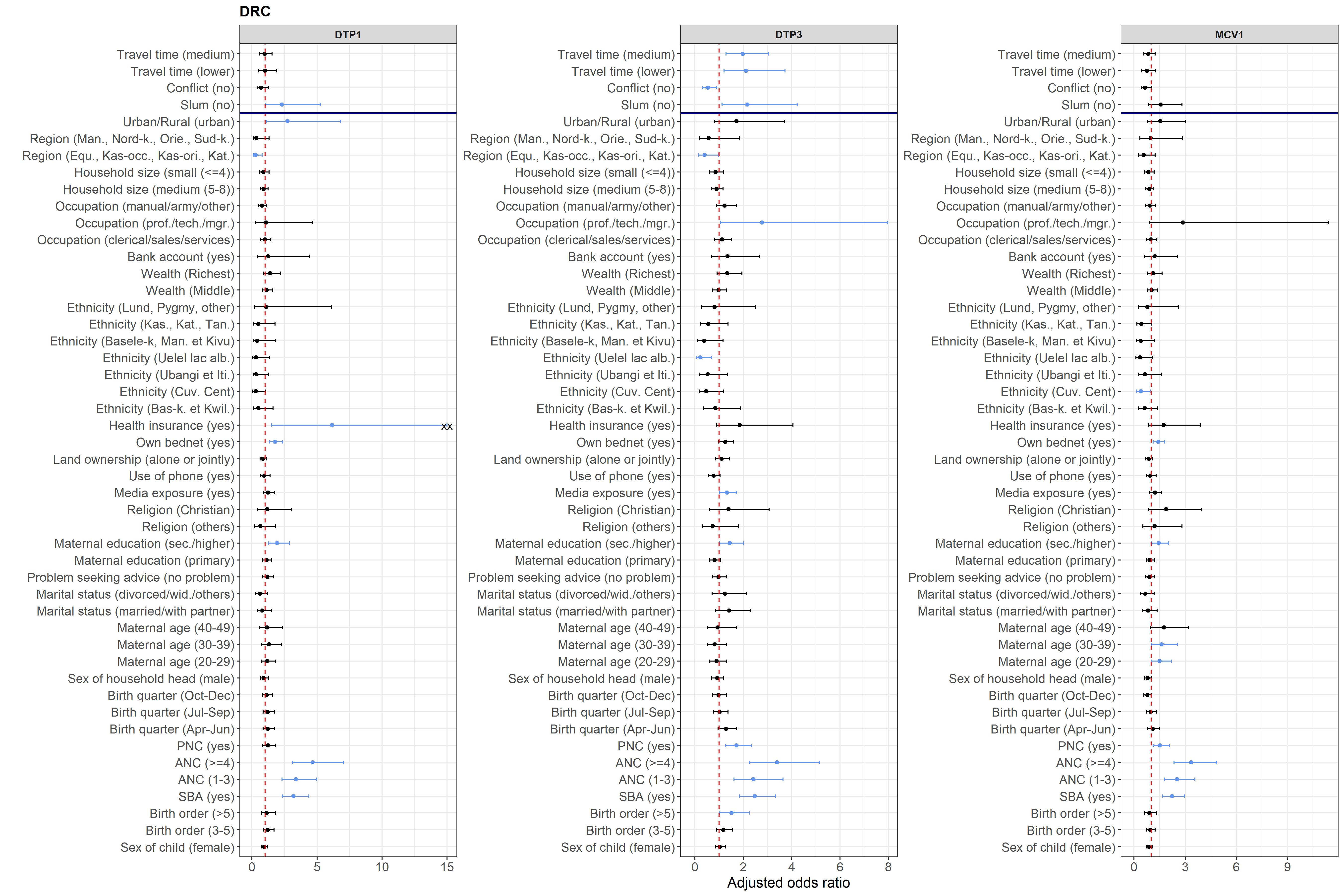
**

**Fig I: Adjusted odds ratio (aOR) and corresponding 95% credible interval (95% CI) plots for DRC. The vertical dotted red lines mark the odds ratio of 1. Light blue dots and lines show the aORs and 95CIs of variables that have significant associations with vaccination. A dark blue horizontal line separates the key community variables from other covariates.**


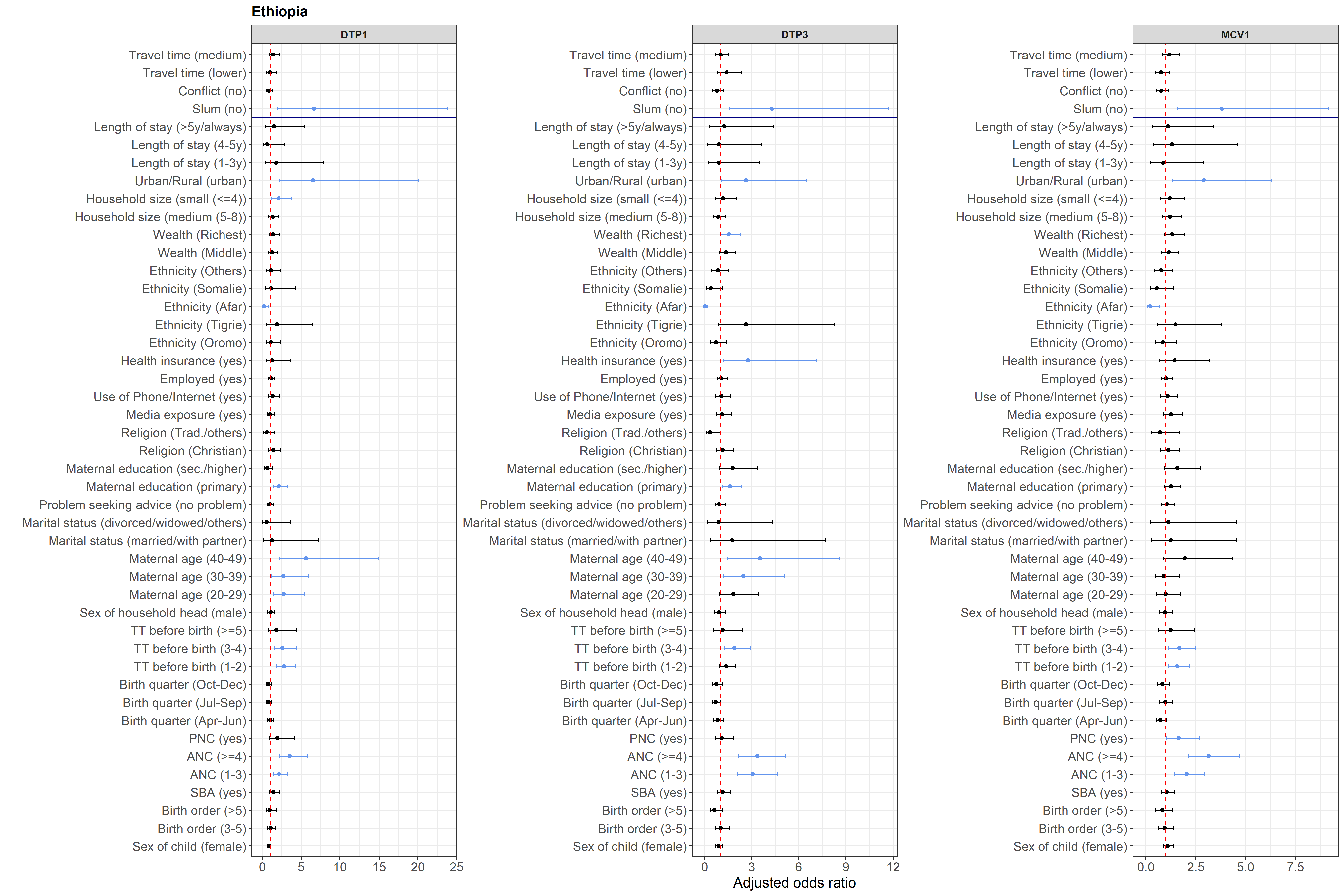


**Fig J: Adjusted odds ratio (aOR) and corresponding 95% credible interval (95% CI) plots for Ethiopia. The vertical dotted red lines mark the odds ratio of 1. Light blue dots and lines show the aORs and 95CIs of variables that have significant associations with vaccination. A dark blue horizontal line separates the key community variables from other covariates.**

**
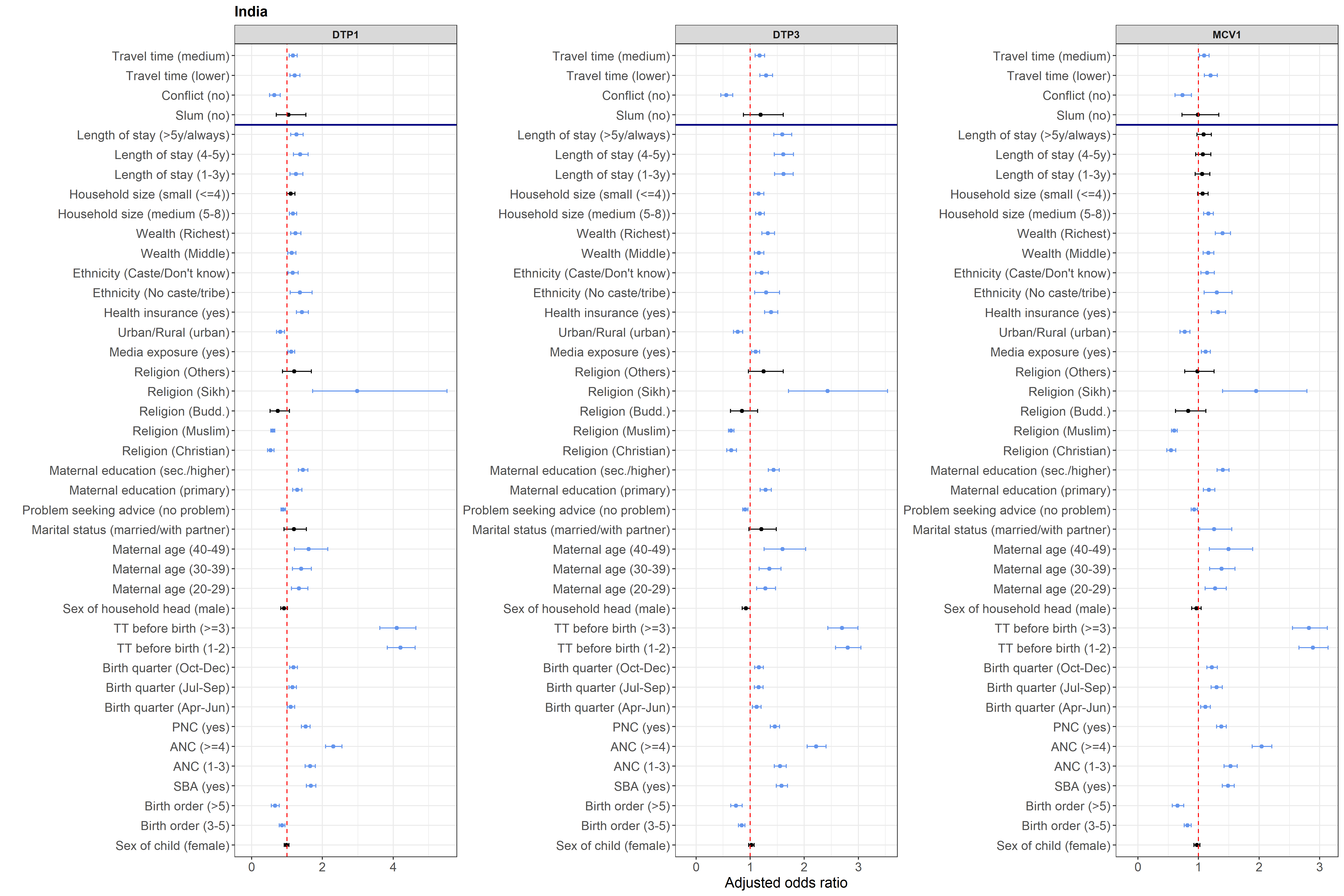
**

**Fig K: Adjusted odds ratio (aOR) and corresponding 95% credible interval (95% CI) plots for India. The vertical dotted red lines mark the odds ratio of 1. Light blue dots and lines show the aORs and 95CIs of variables that have significant associations with vaccination. A dark blue horizontal line separates the key community variables from other covariates.**

**
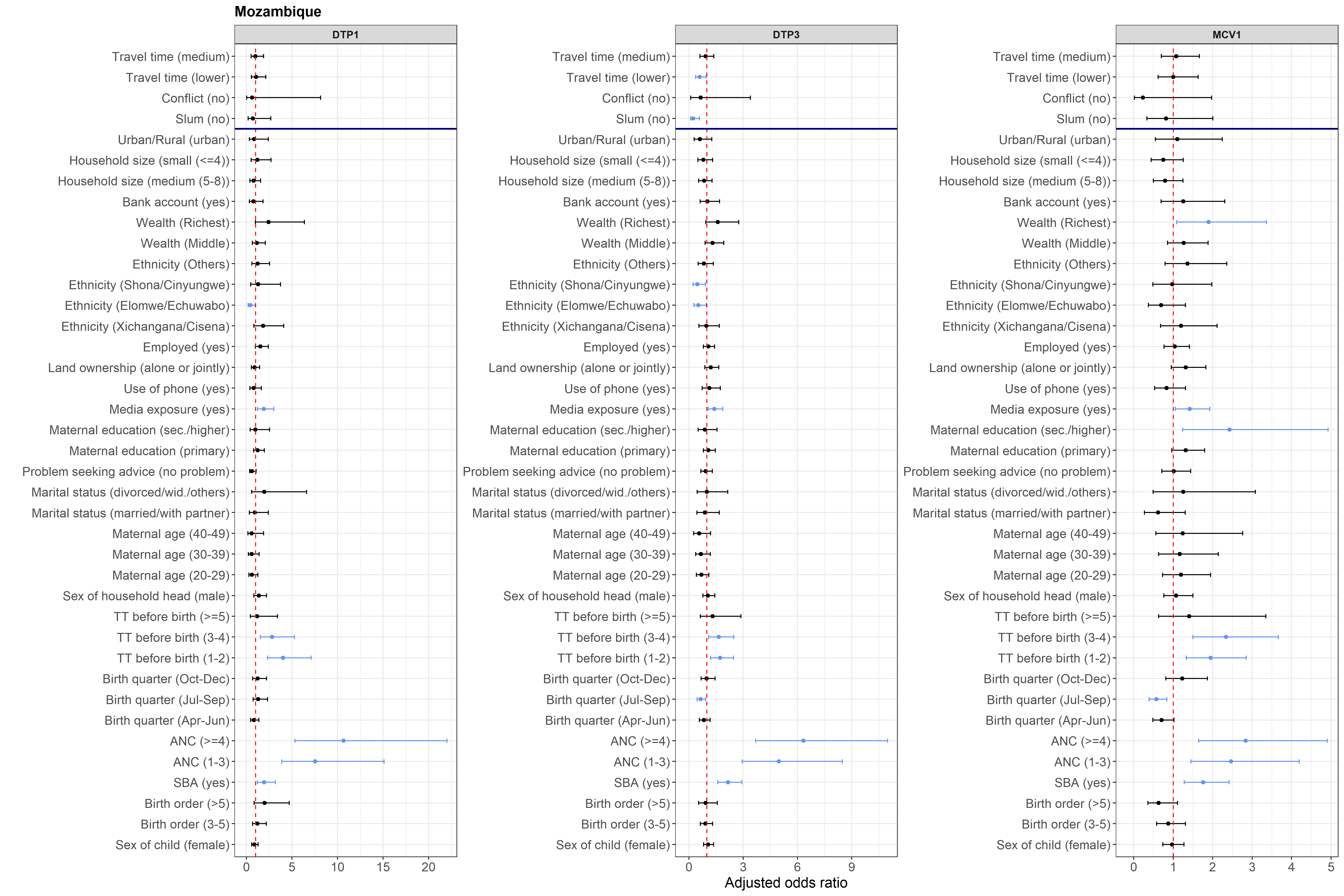
**

**Fig L: Adjusted odds ratio (aOR) and corresponding 95% credible interval (95% CI) plots for Mozambique. The vertical dotted red lines mark the odds ratio of 1. Light blue dots and lines show the aORs and 95CIs of variables that have significant associations with vaccination. A dark blue horizontal line separates the key community variables from other covariates.**

**
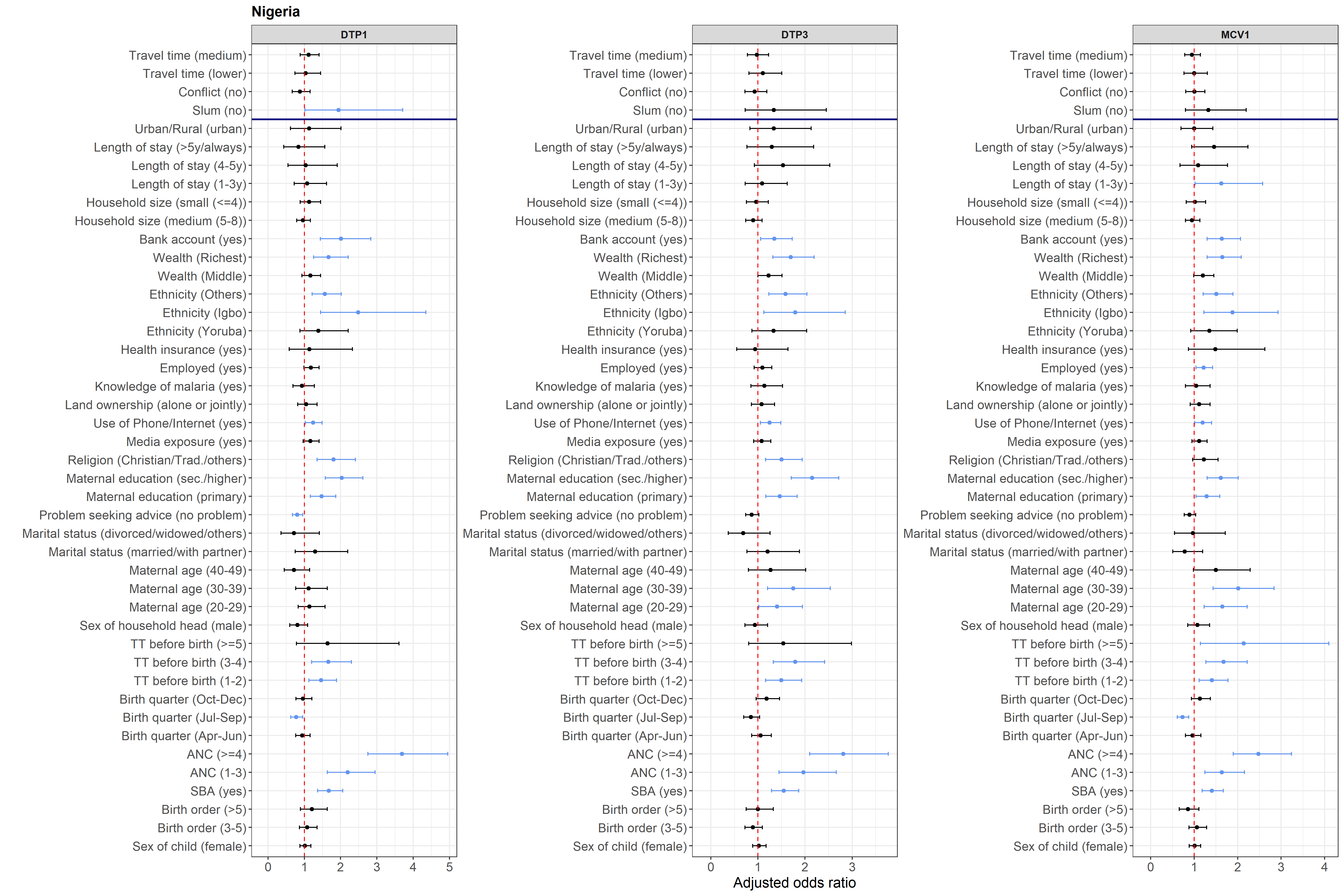
**

**Fig M: Adjusted odds ratio (aOR) and corresponding 95% credible interval (95% CI) plots for Nigeria. The vertical dotted red lines mark the odds ratio of 1. Light blue dots and lines show the aORs and 95CIs of variables that have significant associations with vaccination. A dark blue horizontal line separates the key community variables from other covariates.**

**
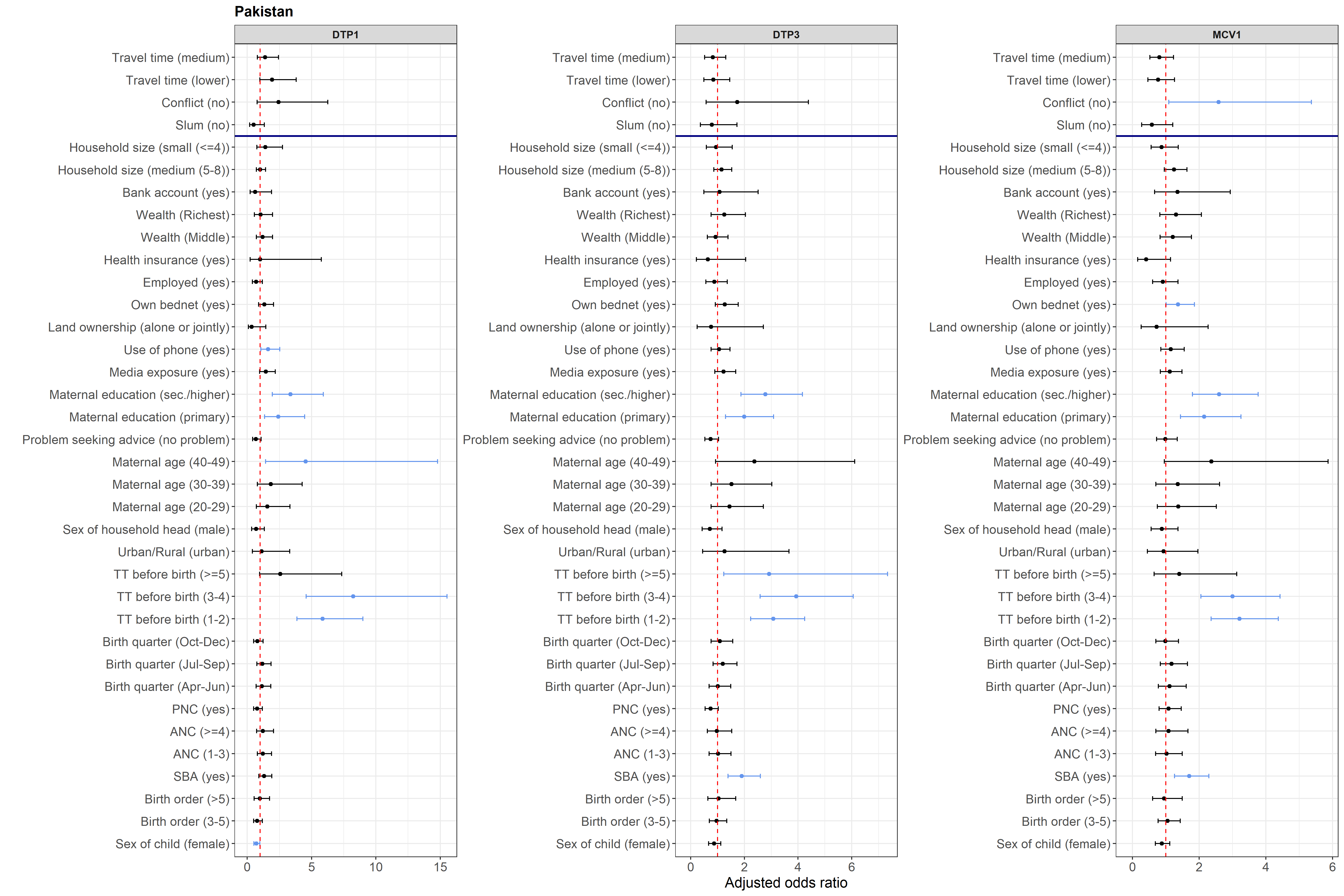
**

**Fig N: Adjusted odds ratio (aOR) and corresponding 95% credible interval (95% CI) plots for Pakistan. The vertical dotted red lines mark the odds ratio of 1. Light blue dots and lines show the aORs and 95CIs of variables that have significant associations with vaccination. A dark blue horizontal line separates the key community variables from other covariates.**

**
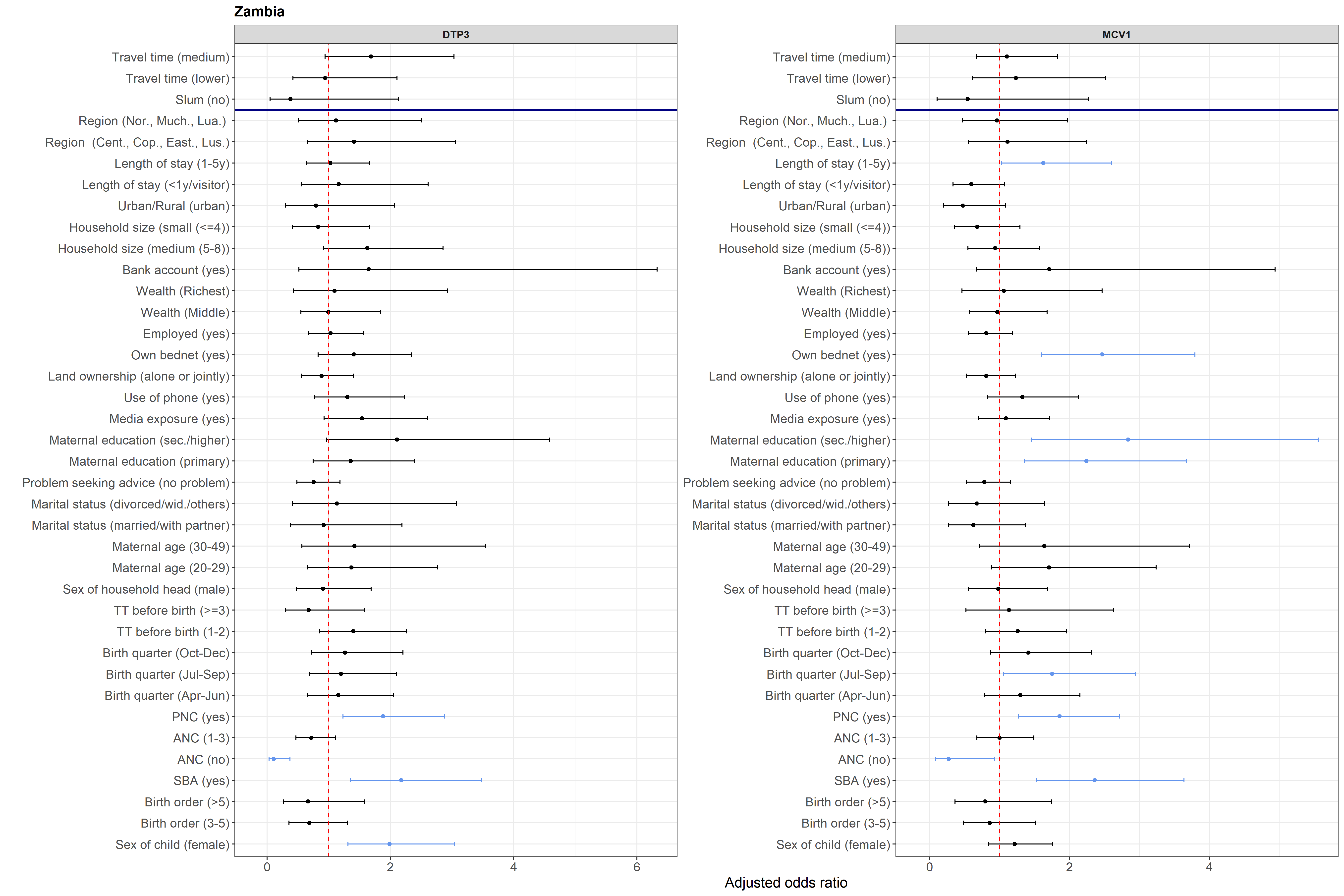
**

**Fig O: Adjusted odds ratio (aOR) and corresponding 95% credible interval (95% CI) plots for Zambia. The vertical dotted red lines mark the odds ratio of 1. Light blue dots and lines show the aORs and 95CIs of variables that have significant associations with vaccination. A dark blue horizontal line separates the key community variables from other covariates.**

**Detailed interpretation of multivariate analysis results for each country**

**Cambodia**

**MCV1**

Antenatal care attendance, birth quarter, mother’s employment status and household wealth were significantly associated with receipt of MCV1. The odds of receiving MCV1 were significantly higher among children from households belonging to the middle (aOR = 1.74, 95% CI: 1.06 – 2.89) and richer/richest wealth index categories (aOR = 3.58, 95% CI: 2.06 – 6.35).

The likelihood of receiving MCV1 vaccination was significantly lower among children born to mothers who made no (aOR = 0.23, 95% CI: 0.11 – 0.48) and between 1-3 antenatal care visits (aOR = 0.49, 95% CI: 0.33 – 0.73) compared to those who made at least 4 antenatal care visits, children who were born between April and June (aOR = 0.54, 95% CI: 0.34 – 0.84) and children born to mothers in employment (aOR = 0.63, 95% CI: 0.42 – 0.94).

**DTP3**

Antenatal care attendance, birth quarter, mother’s receipt of tetanus toxoid (TT) injections, maternal education, household wealth and bank account were significant predictors of being fully vaccinated with all DTP vaccine doses. The odds of DTP3 vaccination were significantly higher among children born to mothers who had received at least 3 TT injections (aOR = 2.00, 95% CI: 1.09 – 3.72), children born to mothers who had at least a secondary education (aOR = 2.60, 95% CI: 1.36 – 5.04), Children from richer/richest households (aOR: 2.42, 95% CI: 1.29 – 4.66) and children born to mothers who owned a bank account (aOR: 2.45, 95% CI: 1.14 - 5.75).

The likelihood of DTP3 vaccination was significantly lower among children born to mothers who made no (aOR = 0.16, 95% CI: 0.07 – 0.36) and between 1-3 antenatal care visits (aOR = 0.34, 95% CI: 0.22 – 0.52), compared to those who made at least 4 antenatal care visits and children born between July and September (aOR = 0.51, 95% CI: 0.30 – 0.85).

**DRC**

**MCV1**

Skilled attendance at birth, antenatal care attendance, postnatal care attendance, maternal age, maternal education, bednet ownership and ethnicity were significant predictors of the receipt of MCV1.

The odds of MCV1 vaccination were significantly higher among children who were delivered by a skilled birth attendant (aOR = 2.23, 95% CI: 1.69 – 2.94), children born to mothers who made 1-3 (aOR = 2.52, 95% CI: 1.77 – 3.58) and at least 4 (aOR = 3.36, 95% CI: 2.34 – 4.84) antenatal care visits during pregnancy, children who had a postnatal check after 2 months of birth (aOR = 1.51, 95% CI: 1.11 – 2.06), Children born to mothers aged 20-29 years (aOR = 1.50, 95% CI: 1.03 – 2.19) and 30-39 years (aOR = 1.62, 95% CI: 1.03 – 2.56), children born to mothers who had at least a secondary education (aOR = 1.46, 95% CI: 1.05 – 2.04), children from households who owned a bednet (aOR = 1.42, 95% CI: 1.12 – 1.80),

The likelihood of MCV1 vaccination was significantly lower among children from the Cuvette Central ethnic group (aOR = 0.40, 95% CI: 0.16 – 0.99).

**DTP1**

Skilled attendance at birth, antenatal care attendance, maternal education, mosquito bednet ownership, health insurance, region (see Fig I), urban/rural residence and living in a non-slum area were significant predictors of the receipt of DTP1.

The odds of DTP1 vaccination were significantly higher among children who were delivered by a skilled birth attendant (aOR = 3.19, 95% CI: 2.34 – 4.38), children born to mothers who made 1-3 (aOR = 3.37, 95% CI: 2.30 – 4.98) and at least 4 antenatal care visits (aOR = 4.66, 95% CI: 3.11 – 7.03) during pregnancy, children born to mothers who had at least a secondary education (aOR = 1.93, 95% CI: 1.29 – 2.87), children from households who owned a bednet (aOR = 1.76, 95% CI: 1.33 – 2.34), children born to mothers who had a health insurance (aOR = 6.14, 95% CI: 1.52 - 34.67), children living in urban areas (aOR = 2.73, 95% CI: 1.13 – 6.83) and children living in non-slum areas (aOR = 2.29, 95% CI: 1.02 – 5.25).

The likelihood of DTP1 vaccination was significantly lower among children from Equateur, Kasai-occidental, Kasai-oriental and Katanga regions (aOR = 0.28, 95% CI: 0.10 – 0.77).

**DTP3**

Birth order, skilled attendance at birth, antenatal care attendance, postnatal care attendance, maternal education, media exposure, ethnicity, occupation, region, living in a non-slum area, living in a non-conflict area and travel time were significant predictors of being full vaccinated with all DTP vaccine doses.

The odds of receiving DTP3 were significantly higher among children with a birth order of at least 5 (aOR = 1.51, 95% CI: 1.02 – 2.24), children who were delivered by a skilled birth attendant (aOR = 2.47, 95% CI: 1.83 – 3.33), children whose mothers had 1-3 antenatal care visits (aOR = 2.42, 95% CI: 1.62 – 3.64) and at least 4 antenatal care visits (aOR = 3.39, 95% CI: 2.25 – 5.15), children who had a postnatal check (aOR = 1.72, 95% CI: 1.28 – 2.33), children born to mothers with at least a secondary education (aOR = 1.44, 95% CI: 1.03 – 2.01), children whose households were exposed to the media (aOR = 1.32, 95% CI: 1.01 – 1.72), children born to mothers in professional, technical or managerial occupation (aOR = 2.78, 95% CI: 1.07 – 7.97), children living in areas with lower (aOR = 2.12, 95% CI: 1.21 – 3.73) and medium travel times (aOR = 1.97, 95% CI: 1.29 – 3.04) and children living in non-slum areas (aOR = 2.17, 95% CI: 1.12 – 4.24).

The odds of being vaccinated were lower among the Uelel ethnic group (aOR = 0.22, 95% CI: 0.07 – 0.70), children living in Equateur, Kasai-occidental, Kasai-oriental and Katanga regions (aOR = 0.41, 95% CI: 0.17 – 0.97). Interestingly, children living in non-conflict areas had a lower chance (aOR = 0.55, 95% CI: 0.33 – 0.91) of being vaccinated with DTP3 compared to those living in conflict areas.

**Ethiopia**

**MCV1**

Antenatal care attendance, postnatal care attendance, maternal receipt of TT injections, ethnicity, urban/rural residence and living in a non-slum area were significantly associated with the receipt of MCV1.

The odds of MCV1 vaccination were significantly higher among children whose mothers made 1-3 (aOR = 2.04, 95% CI: 1.42 – 2.93) and at least 4 (aOR = 3.15, 95% CI: 2.12 – 4.69) antenatal care visits, children who had a postnatal check (aOR = 1.65, 95% CI: 1.04 – 2.68), children whose mothers had 1-2 (aOR = 1.56, 95% CI: 1.13 – 2.17) and 3-4 (aOR =1.68 , 95% CI: 1.14 – 2.48) TT injections, children living in urban areas (aOR = 2.90, 95% CI: 1.34 – 6.32) and children living in non-slum areas (aOR = 3.79, 95% CI: 1.59 – 9.17). MCV1 vaccination was significantly lower among children from Afar ethnic group (aOR = 0.22, 95% CI: 0.07 – 0.67).

**DTP1**

Antenatal care attendance, maternal receipt of TT injections, maternal age, maternal education, ethnicity, urban/rural residence and living in a non-slum area were significantly associated with the receipt of DTP1.

The odds of DTP1 vaccination were significantly higher among children whose mothers made 1-3 (aOR = 2.16, 95% CI: 1.41 – 3.31) and at least 4 (aOR = 3.52, 95% CI: 2.15 – 5.84) antenatal care visits, children whose mothers had 1-2 (aOR = 1.56, 95% CI: 1.13 – 2.17) and 3-4 (aOR =1.68 , 95% CI: 1.14 – 2.48) TT injections, children born to mothers aged 20-29 (aOR = 2.74, 95% CI: 1.37 – 5.43), 30-39 (aOR = 2.68, 95% CI: 1.22 – 5.90) and 40-49 (aOR = 5.61, 95% CI: 2.13 – 14.97), children whose mothers had primary education (aOR = 2.10, 95% CI: 1.37 – 3.25), children from smaller (<=4) households (aOR = 2.08, 95% CI: 1.16 – 3.71), children living urban areas (aOR = 6.51, 95% CI: 2.23 – 20.11) and children living in non-slum areas (aOR = 6.61, 95% CI: 1.88 – 23.84).

DTP1 vaccination was significantly lower among Afar ethnic group (aOR = 0.21, 95% CI: 0.06 – 0.79).

**DTP3**

Antenatal care attendance, maternal receipt of TT injections, maternal age, maternal education, health insurance, ethnicity, household wealth, urban/rural residence and living in a non-slum area were significantly associated with the receipt all DTP vaccine doses.

The odds of DTP3 vaccination were significantly higher among children whose mothers made 1-3 (aOR = 3.08, 95% CI: 2.07 – 4.61) and at least 4 (aOR = 3.35, 95% CI: 2.18 – 5.15) antenatal care visits, children whose mothers had 3-4 TT injections (aOR = 1.90, 95% CI: 1.24 – 2.92), children whose mothers were aged 30-39 years (aOR = 2.47, 95% CI: 1.20 – 5.08) and 40-49 years (aOR = 3.54, 95% CI: 1.47 – 8.56), children whose mothers had a primary education (aOR = 1.63, 95% CI: 1.14 – 2.33), children whose mothers had a health insurance (aOR = 2.77, 95% CI: 1.17 – 7.15), children from the richer/richest households (aOR = 1.54, 95% CI: 1.03 – 2.31), children living in urban areas (aOR = 2.63, 95% CI: 1.08 – 6.46) and children living in non-slum areas (aOR = 4.26, 95% CI: 1.58 – 11.69).

The likelihood of receiving all three DTP doses was found to be significantly lower among the Afar ethnic group (aOR = 0.04, 95% CI: 0.01 – 0.17).

**India**

**MCV1**

Birth order, skilled birth attendance, antenatal care attendance, postnatal care attendance, birth quarter, maternal receipt of TT injections, maternal age, maternal marital status, problem seeking medical advice or treatment, maternal education, religion, media exposure, urban/rural residence, health insurance, ethnicity, household wealth, household size, conflict and travel time were significantly associated with the receipt of MCV1.

The odds of MCV1 vaccination were significantly higher among children delivered by a skilled birth attendant (aOR = 1.49, 95% CI: 1.39 - 1.59), children whose mothers made 1-3 (aOR = 1.53, 95% CI: 1.42 - 1.64) and at least 4 (aOR = 2.04, 95% CI: 1.88 - 2.21) antenatal care visits, children who had a postnatal check (aOR = 1.38, 95% CI: 1.30 - 1.46), children born in April – June (aOR = 1.11, 95% CI: 1.03 - 1.19), July – September (aOR = 1.30, 95% CI: 1.21 - 1.39) and October – December (aOR = 1.22, 95% CI: 1.14 - 1.31), children whose mothers had 1-2 (aOR = 2.89, 95% CI: 2.66 - 3.14) and at least 3 (aOR = 2.82, 95% CI: 2.55 - 3.13) TT injections, children whose mothers were aged 20-29 (aOR = 1.27, 95% CI: 1.11 - 1.46), 30-39 (aOR = 1.38, 95% CI: 1.18 - 1.60) and 40 – 49 (aOR = 1.49, 95% CI: 1.18 - 1.89) years, children whose mothers were married/with their partners (aOR = 1.26, 95% CI: 1.02 - 1.55), children whose mothers had primary (aOR = 1.17, 95% CI: 1.08 - 1.27) and at least secondary education (aOR = 1.40, 95% CI: 1.31 - 1.50), children born to Sikh mothers (aOR = 1.95, 95% CI: 1.40 - 2.79), children whose mothers had media exposure (aOR = 1.12, 95% CI: 1.05 - 1.19), children whose mothers had health insurance (aOR = 1.32, 95% CI: 1.21 - 1.45), children from no caste/tribe (aOR = 1.30, 95% CI: 1.09 - 1.55) and caste ( aOR = 1.14, 95% CI: 1.04 - 1.26) ethnic groups, children from the middle (aOR = 1.16, 95% CI: 1.07 - 1.25) and richer/richest (aOR = 1.40, 95% CI: 1.28 - 1.53) household wealth index categories, children from medium-sized (5-8 members) households (aOR = 1.16, 95% CI: 1.08 - 1.24), and children living in areas with lower (aOR = 1.20, 95% CI: 1.10 - 1.31) and medium travel times (aOR = 1.09, 95% CI: 1.02 - 1.17).

The likelihood of MCV1 vaccination was significantly lower among children with a birth order of 3-5 (aOR = 0.82, 95% CI: 0.76 - 0.88) and > 5 (aOR = 0.65, 95% CI: 0.57 - 0.75), children born to Christian (aOR = 0.55, 95% CI: 0.48 - 0.63) and Muslim (aOR = 0.60, 95% CI: 0.55 - 0.65) mothers. Interestingly, the odds of receiving MCV1 was also significantly lower among children born to mothers who had no problem seeking medical advice/treatment (aOR = 0.93, 95% CI: 0.88 - 0.98 ), children living in urban areas (aOR = 0.77, 95% CI: 0.69 - 0.86) and non-conflict areas (aOR = 0.74, 95% CI: 0.61 - 0.88).

**DTP1**

Birth order, skilled birth attendance, antenatal care attendance, postnatal care attendance, birth quarter, maternal receipt of TT injections, maternal age, problem seeking medical advice or treatment, maternal education, religion, media exposure, urban/rural residence, health insurance, ethnicity, household wealth, household size, length of stay, conflict and travel time were significantly associated with the receipt of DTP1.

The odds of DTP1 vaccination were significantly higher among children delivered by a skilled birth attendant (aOR = 1.67, 95% CI: 1.54 - 1.82), children whose mothers made 1-3 (aOR = 1.65, 95% CI: 1.51 - 1.80) and at least 4 (aOR = 2.31, 95% CI: 2.09 - 2.55 ) antenatal care visits, children who had a postnatal check (aOR = 1.52, 95% CI: 1.40 - 1.65 ), children born in April – June (aOR = 1.11, 95% CI: 1.01 - 1.21 ), July – September (aOR = 1.15, 95% CI: 1.05 - 1.26 ) and October – December (aOR = 1.18, 95% CI: 1.07 - 1.29 ), children whose mothers had 1-2 (aOR = 4.21, 95% CI: 3.83 - 4.62 ) and at least 3 (aOR = 4.10, 95% CI: 3.62 - 4.64 ) TT injections, children whose mothers were aged 20-29 (aOR = 1.34, 95% CI: 1.12 - 1.59 ), 30-39 (aOR = 1.40, 95% CI: 1.15 - 1.69) and 40 – 49 (aOR = 1.61, 95% CI: 1.20 - 2.15 ) years, children whose mothers had primary (aOR = 1.28, 95% CI: 1.16 - 1.42 ) and at least secondary education (aOR = 1.45, 95% CI: 1.32 - 1.59 ), children born to Sikh mothers (aOR = 2.98, 95% CI: 1.72 - 5.52 ), children whose mothers had media exposure (aOR = 1.11, 95% CI: 1.02 - 1.21 ), children whose mothers had health insurance (aOR = 1.42, 95% CI: 1.26 - 1.60), children from no caste/tribe (aOR = 1.36, 95% CI: 1.09 - 1.71 ) and caste (aOR = 1.16, 95% CI: 1.03 - 1.31 ) ethnic groups, children from the middle (aOR = 1.13, 95% CI: 1.02 - 1.25 ) and richer/richest (aOR = 1.24, 95% CI: 1.10 - 1.39 ) household wealth index categories, children from medium-sized (5-8 members) households (aOR = 1.17, 95% CI: 1.07 - 1.27 ), children from households that lived in an area for 1-3 years (aOR = 1.25, 95% CI: 1.08 - 1.45 ), 4-5 years (aOR = 1.37, 95% CI: 1.18 - 1.59 ) and at least 5 years (aOR = 1.27, 95% CI: 1.10 - 1.46), and children living in areas with lower (aOR = 1.21, 95% CI: 1.08 - 1.36) and medium travel times (aOR = 1.17, 95% CI: 1.06 - 1.28).

The likelihood of DTP1 vaccination was significantly lower among children with a birth order of 3-5 (aOR = 0.86, 95% CI: 0.78 -0.94) and > 5 (aOR = 0.66, 95% CI: 0.55 - 0.78), children born to Christian (aOR = 0.53, 95% CI: 0.45- 0.63) and Muslim (aOR = 0.59, 95% CI: 0.54 - 0.66) mothers. Interestingly, the odds of receiving DTP1 was also significantly lower among children born to mothers who had no problem seeking medical advice/treatment (aOR = 0.89, 95% CI: 0.83 - 0.95 ), children living in urban areas (aOR = 0.80, 95% CI: 0.70 - 0.92 ) and non-conflict areas (aOR = 0.64, 95% CI: 0.50 - 0.81).

**DTP3**

Birth order, skilled birth attendance, antenatal care attendance, postnatal care attendance, birth quarter, maternal receipt of TT injections, maternal age, problem seeking medical advice or treatment, maternal education, religion, media exposure, urban/rural residence, health insurance, ethnicity, household wealth, household size, length of stay, conflict and travel time were significantly associated with the receipt of all DTP doses.

The odds of DTP3 vaccination were significantly higher among children delivered by a skilled birth attendant (aOR = 1.58, 95% CI: 1.48 - 1.69), children whose mothers made 1-3 (aOR = 1.55, 95% CI: 1.45 - 1.67) and at least 4 (aOR = 2.22, 95% CI: 2.05 - 2.40) antenatal care visits, children who had a postnatal check (aOR = 1.46, 95% CI: 1.37 - 1.54), children born in April – June (aOR = 1.12, 95% CI: 1.04 - 1.20 ), July – September (aOR = 1.16, 95% CI: 1.08 - 1.24) and October – December (aOR = 1.16, 95% CI: 1.08 - 1.25), children whose mothers had 1-2 (aOR = 2.80, 95% CI: 2.58 - 3.05) and at least 3 (aOR = 2.70, 95% CI: 2.44 - 2.99) TT injections, children whose mothers were aged 20-29 (aOR = 1.28, 95% CI: 1.12 - 1.47), 30-39 (aOR = 1.35, 95% CI: 1.16 -1.57) and 40 – 49 (aOR = 1.60, 95% CI: 1.26 - 2.03) years, children whose mothers had primary (aOR = 1.28, 95% CI: 1.18 - 1.39) and at least secondary education (aOR = 1.43, 95% CI: 1.34 - 1.54), children born to Sikh mothers (aOR = 2.43, 95% CI: 1.71 - 3.54), children whose mothers had media exposure (aOR = 1.10, 95% CI: 1.03 - 1.17 ), children whose mothers had health insurance (aOR = 1.39, 95% CI: 1.27 - 1.51), children from no caste/tribe (aOR = 1.29, 95% CI: 1.09 - 1.54 ) and caste (aOR = 1.21, 95% CI: 1.10 - 1.33 ) ethnic groups, children from the middle (aOR = 1.16, 95% CI: 1.08 - 1.25 ) and richer/richest (aOR = 1.33, 95% CI: 1.22 - 1.45) household wealth index categories, children from medium-sized (5-8 members) (aOR = 1.18, 95% CI: 1.10 - 1.26) and small (<=4 members) (aOR = 1.15, 95% CI: 1.07 - 1.25) households, children whose households had been living in the area for 1-3 years (aOR = 1.62, 95% CI: 1.45 - 1.80), 4-5 years (aOR = 1.61, 95% CI: 1.45 - 1.80 ) and at least 5 years (aOR = 1.59, 95% CI: 1.44 - 1.77) and children living in areas with lower (aOR = 1.29, 95% CI: 1.18 - 1.42) and medium travel times (aOR = 1.18, 95% CI: 1.09 - 1.26).

The likelihood of DTP3 vaccination was significantly lower among children with a birth order of 3-5 (aOR = 0.84, 95% CI: 0.78 - 0.90) and > 5 (aOR = 0.74, 95% CI: 0.64 - 0.85), children born to Christian (aOR = 0.65, 95% CI: 0.57 - 0.75) and Muslim (aOR = 0.65, 95% CI: 0.60 - 0.70) mothers. Interestingly, the odds of receiving DTP3 was also significantly lower among children born to mothers who had no problem seeking medical advice/treatment (aOR = 0.91, 95% CI: 0.86 - 0.96), children living in urban areas (aOR = 0.77, 95% CI: 0.69 - 0.86) and non-conflict areas (aOR = 0.56, 95% CI: 0.46 - 0.68).

**Madagascar**

**MCV1**

Birth order, antenatal care attendance, birth quarter, maternal receipt of TT injections, maternal education, religion, maternal employment status, household size, region and travel time were significantly associated with the receipt of MCV1.

The odds of MCV1 vaccination were significantly higher among children whose mothers made 1-3 (aOR = 2.04, 95% CI: 1.34 - 3.12 ) and at least 4 (aOR = 2.95, 95% CI: 1.92 - 4.56) antenatal care visits, children born in April – June (aOR = 1.38, 95% CI: 1.01- 1.88) and July – September (aOR = 1.99, 95% CI: 1.42 - 2.81), children whose mothers had 1-2 (aOR = 1.97, 95% CI: 1.50 - 2.61) and 3 – 4 (aOR = 2.76, 95% CI: 1.85 - 4.16) TT injections, children whose mothers had primary (aOR = 1.51, 95% CI: 1.14 - 2.01) and at least secondary education (aOR = 2.13, 95% CI: 1.37 - 3.35), children born to Christian mothers (aOR = 1.42, 95% CI: 1.07 - 1.89), children whose mothers were in employment (aOR = 2.31, 95% CI: 1.45 - 3.67), children living in areas with lower (aOR = 2.28, 95% CI: 1.49 - 3.52) and medium travel times (aOR = 1.89, 95% CI: 1.32 - 2.71), and children from the Atsimo Atsinanana, Atsinanana, Analanjirofo and Alaotra Mangoro regions (aOR = 2.50, 95% CI: 1.18 - 5.60).

The likelihood of MCV1 vaccination was significantly lower among children with a birth order > 5 (aOR = 0.48, 95% CI: 0.30 - 0.77), children from medium-sized (5-8 members) (aOR = 0.69, 95% CI: 0.47 - 0.99) and small (<= 4 members) (aOR = 0.56, 95% CI: 0.37 - 0.84) households.

**DTP1**

Skilled birth attendance, antenatal care attendance, birth quarter, maternal receipt of TT injections, maternal marital status, maternal education, religion, maternal employment status, household size, and travel time were significantly associated with the receipt of DTP1.

The odds of DTP1 vaccination were significantly higher among children delivered by a skilled birth attendant (aOR = 1.62, 95% CI: 1.11 - 2.38), children whose mothers made 1-3 (aOR = 3.05, 95% CI: 1.92 - 4.87) and at least 4 (aOR = 3.93, 95% CI: 2.43 - 6.41) antenatal care visits, children born in July – September (aOR = 1.84, 95% CI: 1.18 - 2.90), children whose mothers had 1-2 (aOR = 3.43, 95% CI: 2.39 - 4.95) and 3 – 4 (aOR = 3.27, 95% CI: 1.95 - 5.63) TT injections, children whose mothers had primary (aOR = 1.60, 95% CI: 1.12 - 2.28) and at least secondary education (aOR = 2.27, 95%CI: 1.23 - 4.31), children born to Christian mothers (aOR = 1.75, 95% CI: 1.21 - 2.54), children whose mothers were in employment (aOR = 2.80, 95% CI: 1.52 - 5.06) and children living in areas with lower (aOR = 2.88, 95% CI: 1.60 - 5.30) and medium travel times (aOR = 1.96, 95% CI: 1.22 - 3.18).

The likelihood of DTP1 vaccination was significantly lower among children from small (<= 4 members) (aOR = 0.54, 95% CI: 0.31 - 0.93) households and children whose mothers were divorced/widowed/separated (aOR = 0.37, 95% CI: 0.13 - 0.96).

**DTP3**

Skilled birth attendance, antenatal care attendance, birth quarter, maternal receipt of TT injections, maternal education, religion, mobile phone ownership, maternal employment status, household size, region and travel time were significantly associated with the receipt of all three DTP doses.

The odds of DTP3 vaccination were significantly higher among children delivered by a skilled birth attendant (aOR = 1.61, 95% CI: 1.19 - 2.19), children whose mothers made 1-3 (aOR = 2.39, 95% CI: 1.53 - 3.78) and at least 4 (aOR = 3.48, 95% CI: 2.19 - 5.57) antenatal care visits, children born in July – September (aOR = 1.62, 95% CI: 1.12 - 2.36), children whose mothers had 1-2 (aOR = 2.24, 95% CI: 1.65 - 3.05) and 3 – 4 (aOR = 2.87, 95% CI: 1.86 - 4.48) TT injections, children whose mothers had primary (aOR = 1.67, 95% CI: 1.22 - 2.28) and at least secondary education (aOR = 2.04, 95% CI: 1.25 - 3.35), children born to Christian mothers (aOR = 1.64, 95% CI: 1.19 - 2.26), children whose mothers owned mobile phones (aOR = 1.82, 95% CI: 1.12 - 2.99), children whose mothers were in employment (aOR = 1.82, 95% CI: 1.09 - 3.02) and children living in areas with lower (aOR = 3.32, 95% CI: 1.95 - 5.75) and medium travel times (aOR = 2.05, 95% CI: 1.32 - 3.20).

The likelihood of DTP3 vaccination was significantly lower among children from small (<= 4 members) (aOR = 0.61, 95% CI: 0.39 - 0.95) households and children from Atsimo Andrefana, Androy, Anosy and Menabe regions (aOR = 0.29, 95% CI: 0.11 - 0.79).

**Mozambique**

**MCV1**

Skilled birth attendance, antenatal care attendance, maternal receipt of TT injections, maternal education and household wealth were significantly associated with the receipt of MCV1.

The odds of MCV1 vaccination were significantly higher among children who were delivered by a skilled birth attendant (aOR = 1.76, 95% CI: 1.28 - 2.41), children whose mothers made 1-3 (aOR = 2.47, 95% CI: 1.45 - 4.19) and at least 4 (aOR = 2.84, 95% CI: 1.65 - 4.90) antenatal care visits, children whose mothers had 1-2 (aOR = 1.95, 95% CI: 1.33 - 2.85) and 3 – 4 (aOR = 2.34, 95% CI: 1.50 - 3.66) TT injections, children whose mothers had at least secondary education (aOR = 2.43, 95% CI: 1.24 - 4.92) and children from households in the richer/richest wealth category (aOR = 1.90, 95% CI: 1.09 - 3.36).

**DTP1**

Skilled birth attendance, antenatal care attendance, maternal receipt of TT injections, media exposure and ethnicity were significantly associated with the receipt of DTP1. The odds of DTP1 vaccination were significantly higher among children who were delivered by a skilled birth attendant (aOR = 1.94, 95% CI: 1.20 - 3.16), children whose mothers made 1-3 (aOR = 7.53, 95% CI: 3.88 - 15.11) and at least 4 (aOR = 10.65, 95% CI: 5.33 - 22.00) antenatal care visits, children whose mothers had 1-2 (aOR = 4.02, 95% CI: 2.30 - 7.11) and 3 – 4 (aOR = 2.79, 95% CI: 1.51 - 5.26) TT injections and children whose mothers were exposed to the media (aOR = 1.90, 95% CI: 1.22 - 3.01).

The likelihood of DTP3 vaccination was significantly lower among children from the Elome and Echuwabo ethnic groups (aOR = 0.43, 95% CI: 0.19 - 0.99).

**DTP3**

Skilled birth attendance, antenatal care attendance, birth quarter, maternal receipt of TT injections, media exposure, ethnicity, living in a non-slum area and travel time were significantly associated with the receipt of all 3 DTP doses.

The odds of DTP3 vaccination were significantly higher among children who were delivered by a skilled birth attendant (aOR = 2.16, 95% CI: 1.60 - 2.93), children whose mothers made 1-3 (aOR = 4.96, 95% CI: 2.94 - 8.47) and at least 4 (aOR = 6.32, 95% CI: 3.68 - 10.97) antenatal care visits, children born between July and September (aOR = 0.65, 95% CI: 0.45 - 0.93), children whose mothers had 1-2 (aOR = 1.72, 95% CI: 1.20 - 2.46) and 3 – 4 (aOR = 1.65, 95% CI: 1.10 - 2.48) TT injections and children whose mothers were exposed to the media (aOR = 1.41, 95% CI: 1.06 - 1.87).

The likelihood of DTP3 vaccination was significantly lower among children from the Elome/Echuwabo (aOR = 0.52, 95% CI: 0.28 - 0.97) and Shona/Cinyungwe (aOR = 0.47, 95% CI: 0.24 - 0.91) ethnic groups. Interestingly, DTP3 vaccination was also significantly lower among children living in non-slum areas (aOR = 0.24, 95% CI: 0.10 - 0.59) and children from areas with lower travel times (aOR = 0.60, 95% CI: 0.37 - 0.96).

**Nigeria**

**MCV1**

Skilled birth attendance, antenatal care attendance, birth quarter, maternal receipt of TT injections, maternal age, maternal education, maternal use of phone/internet, maternal employment status, ethnicity, household wealth, ownership of a bank account and length of stay were significantly associated with the receipt of MCV1.

The odds of MCV1 vaccination were significantly higher among children who were delivered by a skilled birth attendant (aOR = 1.41, 95% CI: 1.18 - 1.67), children whose mothers made 1-3 (aOR = 1.64, 95% CI: 1.24 - 2.16) and at least 4 (aOR = 2.48, 95% CI: 1.90 - 3.24) antenatal care visits, children whose mothers had 1-2 (aOR = 1.41, 95% CI: 1.11 - 1.78), 3 – 4 (aOR = 1.68, 95% CI: 1.27 - 2.22) and at least 5 (aOR = 2.14, 95% CI: 1.14 - 4.10) TT injections, children born to mothers aged 20-29 years (aOR = 1.65, 95% CI: 1.23 - 2.22) and 30 – 39 years (aOR = 2.02, 95% CI: 1.43 - 2.84), children whose mothers had primary (aOR = 1.29, 95% CI: 1.04 - 1.59) and at least secondary education (aOR = 1.62, 95% CI: 1.30 - 2.01), children whose mothers had access to phones/internet (aOR = 1.19, 95% CI: 1.01 - 1.40), children whose mothers were in employment (aOR = 1.22, 95% CI: 1.04 - 1.42), children from the Igbo (aOR = 1.88, 95% CI: 1.22 - 2.93) and Other (aOR = 1.51, 95% CI: 1.21 - 1.90) ethnic groups, children from households in the richer/richest wealth category (aOR = 1.64, 95% CI: 1.30 - 2.08), children whose mothers owned a bank account (aOR = 1.63, 95% CI: 1.30 - 2.06) and children whose households had lived in the area for 1-3 years (aOR = 1.63, 95% CI: 1.02 - 2.57).

The likelihood of MCV1 vaccination was significantly lower among children born between July and September (aOR = 0.73, 95% CI: 0.61 - 0.88).

**DTP1**

Skilled birth attendance, antenatal care attendance, birth quarter, maternal receipt of TT injections, problem getting medical advice/treatment, maternal education, religion, maternal use of phone/internet, ethnicity, household wealth, ownership of a bank account and living in a non-slum area were significantly associated with the receipt of DTP1.

The odds of DTP1 vaccination were significantly higher among children who were delivered by a skilled birth attendant (aOR = 1.67, 95% CI: 1.37 - 2.06), children whose mothers made 1-3 (aOR = 2.19, 95% CI: 1.63 - 2.95) and at least 4 (aOR = 3.69, 95% CI: 2.75 - 4.95) antenatal care visits, children whose mothers had 1-2 (aOR = 1.46, 95% CI: 1.13 - 1.89) and 3 – 4 (aOR = 1.66, 95% CI: 1.20 - 2.30) TT injections, children whose mothers had primary (aOR = 1.47, 95% CI: 1.17 - 1.87) and at least secondary education (aOR = 2.03, 95% CI: 1.58 - 2.61), children born to mothers practising Christian/tradition/other religions (aOR = 1.80, 95% CI: 1.35 - 2.41), children whose mothers had access to phones/internet (aOR = 1.24, 95% CI: 1.03 - 1.49), children from the Igbo (aOR = 2.49, 95% CI: 1.44 - 4.35) and Other (aOR = 1.56, 95% CI: 1.21 - 2.02) ethnic groups, children from households in the richer/richest wealth category (aOR = 1.66, 95% CI: 1.26 - 2.21), children whose mothers owned a bank account (aOR = 2.01, 95% CI: 1.44 - 2.83) and children living in non-slum areas (aOR = 1.94, 95% CI: 1.02 - 3.71).

The likelihood of DTP1 vaccination was significantly lower among children born between July and September (aOR = 0.77, 95% CI: 0.63 - 0.95). Interestingly, was also significantly lower among children whose mothers had no problem seeking medical advice/treatment (aOR = 0.80, 95% CI: 0.67 - 0.95).

**DTP3**

Skilled birth attendance, antenatal care attendance, maternal receipt of TT injections, maternal age, maternal education, religion, maternal use of phone/internet, ethnicity, household wealth and ownership of a bank account were significantly associated with the receipt of all three DTP doses.

The odds of DTP3 vaccination were significantly higher among children who were delivered by a skilled birth attendant (aOR = 1.55, 95% CI: 1.29 - 1.86), children whose mothers made 1-3 (aOR = 1.97, 95% CI: 1.45 - 2.66) and at least 4 (aOR = 2.81, 95% CI: 2.10 - 3.77) antenatal care visits, children whose mothers had 1-2 (aOR = 1.50, 95% CI: 1.16 - 1.93) and 3 – 4 (aOR = 1.79, 95% CI: 1.33 - 2.41) TT injections, children born to mothers aged 20-29 years (aOR = 1.41, 95% CI: 1.02 - 1.94) and 30-39 years (aOR = 1.75, 95% CI: 1.21 -2.54), children whose mothers had primary (aOR = 1.46, 95% CI: 1.17 - 1.83) and at least secondary education (aOR = 2.15, 95% CI: 1.71 - 2.71), children born to mothers practising the Christian/traditional/other religions (aOR = 1.50, 95% CI: 1.16 -1.94), children whose mothers had access to phones/internet (aOR = 1.25, 95% CI: 1.05 - 1.49), children from the Igbo (aOR = 1.79, 95% CI: 1.13 - 2.86) and Other (aOR = 1.59, 95% CI: 1.23 - 2.04) ethnic groups, children from households in the richer/richest wealth category (aOR = 1.70, 95% CI: 1.31 - 2.19) and children whose mothers owned a bank account (aOR = 1.35, 95% CI: 1.05 - 1.73).

**Pakistan**

**MCV1**

Skilled birth attendance, maternal receipt of TT injections, maternal education, ownership of a bednet and conflict were significantly associated with the receipt of MCV1.

MCV1 vaccination was significantly higher among children who were delivered by a skilled birth attendant (aOR = 1.70, 95% CI: 1.26 - 2.29),children whose mothers had 1-2 (aOR = 3.21, 95% CI: 2.36 - 4.38) and 3 – 4 (aOR = 3.00, 95% CI: 2.05 - 4.43) TT injections, children whose mothers had primary (aOR = 2.15, 95% CI: 1.44 - 3.25) and at least secondary education (aOR = 2.60, 95% CI: 1.80 - 3.77), children whose households owned a bednet (aOR = 1.36, 95% CI: 1.00 - 1.86) and children living in non-conflict areas (aOR = 2.58, 95% CI: 1.09 - 5.37).

**DTP1**

Maternal receipt of TT injections, maternal age, maternal education and ownership of a mobile phone were significantly associated with the receipt of DTP1.

DTP1 vaccination was significantly higher among children whose mothers had 1-2 (aOR = 5.85, 95% CI: 3.87 - 8.99) and 3 – 4 (aOR = 8.23, 95% CI: 4.58 - 15.52) TT injections before birth, children whose mothers were aged 40-49 years (aOR = 4.55, 95% CI: 1.43 - 14.79), children whose mothers had primary (aOR = 2.41, 95% CI: 1.35 - 4.46) and at least secondary education (aOR = 3.36, 95% CI: 1.95 - 5.91 ) and children whose mothers owned a mobile phone (aOR = 1.62, 95% CI: 1.05 - 2.53).

**DTP3**

Skilled birth attendance, maternal receipt of TT injections and maternal education were significantly associated with the receipt of all three DTP doses.

DTP3 vaccination was significantly higher among children who were delivered by a skilled birth attendant (aOR = 1.90, 95% CI: 1.40 - 2.60), children whose mothers had 1-2 (aOR = 3.07, 95% CI: 2.23 - 4.25), 3 – 4 (aOR = 3.93, 95% CI: 2.59 - 6.05) and at least 5 (aOR = 2.92, 95% CI: 1.24 - 7.33) TT injections before birth, and children whose mothers had primary (aOR = 1.99, 95% CI: 1.30 - 3.09) and at least secondary education (aOR = 2.78, 95% CI: 1.87 - 4.16).

**Zambia**

**MCV1**

Skilled birth attendance, antenatal care attendance, postnatal care attendance, birth quarter, maternal education, ownership of a bednet and length of stay were significantly associated with the receipt of MCV1.

The odds of MCV1 vaccination were significantly higher among children who were delivered by a skilled birth attendant (aOR = 2.36, 95% CI: 1.53 - 3.64), children who had a postnatal check (aOR = 1.86, 95% CI: 1.27 - 2.72), children born between July and September (aOR = 1.75, 95% CI: 1.05 - 2.94), children whose mothers had primary (aOR = 2.24, 95% CI: 1.36 - 3.67) and at least secondary education (aOR = 2.84, 95% CI: 1.46 - 5.56), children whose households owned a bednet (aOR = 2.47, 95% CI: 1.60 - 3.79) and children whose households had been in the area for 1-5 years (aOR = 1.63, 95% CI: 1.03 - 2.61).

The likelihood of MCV1 vaccination was significantly lower among children whose mothers made no antenatal care visits (aOR = 0.28, 95% CI: 0.08 - 0.93).

**DTP3**

Sex of child, skilled birth attendance, antenatal care attendance and postnatal care attendance were significantly associated with the receipt of all 3 DTP doses.

The odds of DTP3 vaccination were significantly higher among female children (aOR = 1.99, 95% CI: 1.31 - 3.04), children who were delivered by a skilled birth attendant (aOR = 2.18, 95% CI: 1.35 - 3.48) and children who had a postnatal check (aOR = 1.88, 95% CI: 1.23 - 2.88).

The likelihood of DTP3 vaccination was significantly lower among children whose mothers made no antenatal care visits (aOR = 0.11, 95% CI: 0.03 - 0.37).

**References:**

1. Assaf S, Juan C. Stunting and Anemia in Children from Urban Poor Environments in 28 Low and Middle-income Countries: A Meta-analysis of Demographic and Health Survey Data. Nutrients. 2020;12(11):3539.

2. Fink G, Günther I, Hill K. Slum Residence and Child Health in Developing Countries. Demography. 2014;51(4):1175-97.

3. Weiss DJ, Nelson A, Gibson HS, Temperley W, Peedell S, Lieber A, et al. A global map of travel time to cities to assess inequalities in accessibility in 2015. Nature. 2018;553(7688):333-6.

4. ICF International. Demographic and Health Surveys (various) [Datasets]. Calverton, Maryland, U.S.A.: ICF International [Distributor]; 2009-2018.

5. R Core Team. A Language and Environment for Statistical Computing. Vienna, Austria 2021.

6. Fox J, Monette G. Generalized Collinearity Diagnostics. Journal of the American Statistical Association. 1992;87(417):178-83.

7. Snidjers TAB, Bosker RJ. Multilevel analysis. 2nd ed. London: SAGE Publications Ltd; 2012.

8. Adeyinka DA, Muhajarine N, Petrucka P, Isaac EW. Inequities in child survival in Nigerian communities during the Sustainable Development Goal era: insights from analysis of 2016/2017 Multiple Indicator Cluster Survey. BMC Public Health. 2020;20(1):1613.

9. Browne WJ, Subramanian SV, Jones K, Goldstein H. Variance partitioning in multilevel logistic models that exhibit overdispersion. Journal of the Royal Statistical Society: Series A (Statistics in Society). 2005;168(3):599-613.

10. Hosmer DW, Lemeshow S, Sturdivant RX. Applied Logistic Regression. 3rd ed. Hoboken, New Jersey: John Wiley & Sons, Inc; 2013.

11. Rue H, Martino S, Lindgren F, Simpson D, Riebler A. R-INLA: Approximate Bayesian inference using Integrated Nested Laplace Approximations. Trondheim Norway; 2013.

12. StataCorp. Stata Statistical Software: Release 16. College Station, TX: StataCorp LLC.2019.

13. Utazi CE, Thorley J, Alegana VA, Ferrari MJ, Takahashi S, Metcalf CJE, et al. Mapping vaccination coverage to explore the effects of delivery mechanisms and inform vaccination strategies. Nature Communications. 2019;10(1):1633.

14. Utazi CE, Thorley J, Alegana VA, Ferrari MJ, Takahashi S, Metcalf CJE, et al. High resolution age-structured mapping of childhood vaccination coverage in low and middle income countries. Vaccine. 2018;36(12):1583-91.

15. Utazi CE, Wagai J, Pannell O, Cutts FT, Rhoda DA, Ferrari MJ, et al. Geospatial variation in measles vaccine coverage through routine and campaign strategies in Nigeria: Analysis of recent household surveys. Vaccine. 2020;38(14):3062-71.
