# Supplementary material for: Assessing the characteristics of un- and under-vaccinated children in low- and middle-income countries: A multi-level cross-sectional study
Source: PLOS Glob Public Health. 2022 Apr 27;2(4):e0000244. doi: 10.1371/journal.pgph.0000244 (PMC10021434; doi:10.1371/journal.pgph.0000244)
Supplement: Attachment — Submitted filename: Response_to_reviewers_comments.docx [file pgph.0000244.s002.docx]

**Response to reviewers’ comments**

Journal Requirements:

1. Please amend your detailed Financial Disclosure statement. This is published with the article, therefore should be completed in full sentences and contain the exact wording you wish to be published.

i). State the initials, alongside each funding source, of each author to receive each grant.
ii). State what role the funders took in the study. If the funders had no role in your study, please state: “The funders had no role in study design, data collection and analysis, decision to publish, or preparation of the manuscript.”

Response: Done. Thank you.

**Reviewer #1**: Review for PGPH-D-21-00618 - Assessing the characteristics of un- and under-vaccinated children in low- and middle-income countries: A multi-level cross-sectional study

I appreciate the invitation to review this manuscript that addresses an important public health issue: vaccination coverage, especially in vulnerable populations living in low- and middle-income countries. The manuscript is original and has been well-designed methodologically.

I only have some general comments and some minor ones, as described below:

Response: Thank you for these positive comments. We have now addressed your general and minor comments as given below.

**General comments**

Descriptive studies have several important roles in medical and public health research. Typically, descriptive data provide information to develop hypotheses on causes or risk factors for illness (outcomes). In these sense, I strongly suggest that authors avoid using the term "risk" when describing and interpreting study results, since the risk can only be assessed in analytic studies. The authors must remember that, even using the odds ratio as a measure of association, this is a descriptive study.
This recommendation is directly in line with the objective of the study, which is well-proposed: "to investigate and quantify the relationships".

Response: Thank you for this comment. First, we would like to clarify that from a statistical standpoint, our work involves both descriptive and inferential elements and does not qualify to be described as a descriptive study. The bivariate and multivariate analyses we carried out involved fitting single and multi-level binary logistic regression models to quantify the associations between the odds of vaccination and the covariates explored in the study. These analyses are not descriptive.

Secondly, we appreciate the point about the use of the term “risk” when interpreting the results of the study. We had used the terms “risk”, “likelihood” and “odds” interchangeably, but we recognize that there can be subtle differences between “odds” and these other terms as highlighted in this article ^1^. Given that we calculated odds ratios in both the bivariate and multivariate analyses we carried out, we have now used only the term “odds” throughout the manuscript when interpreting the results for correctness and consistency.

ABSTRACT
Findings: odds instead risk.

Response: Thank you. Done.

INTRODUCTION
Page 5, line 111: this is the first appearance of the acronym DRC, therefore, I suggest presenting the country name in full: Democratic Republic of the Congo [DRC].

Response: Done. Thanks.

METHODS
Page 5, lines 190-196: this paragraph should be moved to the Results.

Response: Thank you for this comment. Estimating coverage levels for the study countries was not one of the objectives of the study. The figures/estimates mentioned here only serve to describe the data that we analyzed. Including summary statistics of the data to be analysed in the data section is common practice considering that these are not part of the results of our study. Hence, we have not made any changes to the manuscript.

DISCUSSION
Page 11, lines 369: odds instead risk.

Response: Thank you. Done.

**Reviewer #2**: This is a well-written paper about vaccinations in low and middle-income countries.

The authors claim that «Children living in remote-rural, urban slums and conflict- affected settings are more likely to be un- or under-vaccinated in some countries. Certain household characteristics, including lack of access to maternal health services and other gender-related barriers to vaccination, were consistent predictors of children missing out on vaccination, and our results suggest integrated delivery strategies deserve more consideration».

The results are done in several countries and are like what is known before. However, the title and the methods suggest that they did a multilevel study, and that they also used Bayesian multi-level binary logistic regression models. That could make this paper interesting and provide novel information.

Response: Thank you for these comments. As we have already mentioned in our manuscript, apart from the robust methods used in our work, its novelty also lies in the fact that, to our knowledge, it is the first in-depth quantitative study utilizing cross-sectional individual-level data to explore key non- and under-vaccination characteristics simultaneously across the Equity Reference Group for Immunization’s priority settings.

Unfortunately, neither their methods nor the results describe the Bayesian multi-level binary logistic regressions. It seems as if a simple logistic regression has been done. Do the different inter and intra-country levels give important additional information about how vaccines are used?

Response: Thank you for this comment. The methods used in our work are briefly described in the statistical analysis section. A detailed description of the methods, including the multi-level model used for our multivariate analyses and additional metrics used for residual analysis (i.e. analysis of the random effects included in the models) is included in the analysis section of the supplementary materials as we have further clarified in the manuscript. We fitted country-specific models, in which we accounted for the nested structure of our data, namely child, household, community and stratum levels. Although our approach is standardized across the study countries, we did not model inter-country variation directly. We are, therefore, unable to comment on this. We have included a comment in the discussion to clarify this.

Furthermore, it is also a requirement that when researchers use Bayesian statistics that they discuss the issue of prior probability. That has not been done in this study. Equally important, the results are not presented in a way that Bayesian statistics is usually done with credible intervals, and there is no information about the ICCs for the many levels that they studied.

Response: Thank you for these comments, although we find them surprising. First, the prior distributions used in our work are detailed in the analysis section of the supplementary file. The reasons for choosing these priors are also given.

Throughout the manuscript, we presented both the point and interval (confidence intervals for the frequentist bivariate analyses and credible intervals for the Bayesian multivariate analyses) estimates of the fixed effects. These are given in Figures 2 and 3 in the main manuscript and Tables 5-15 and Figures 8-15 in the supplementary file.

Our residual analyses included the calculation of percentage change in total residual variation and the variance partition coefficient, which are substitutes for ICC and more informative for our analysis. We also calculated the AUC statistics of the fitted models to assess their predictive power. We intentionally moved the residual analysis and model evaluation results to the supplementary materials as we wanted to focus on discussing and interpreting the fixed effects in the main manuscript. We have edited both the methods and results sections to clarify this further.

Reviewer #3: The Paper focuses on characterising zero-dose and under vaccinated communities within LMIC countries using a appropriate methodology. The data used in in the public domain and the data and results support the conclusions made.

Response: Thank you for this positive comment about our work.

**References**

1. Ranganathan, P., Aggarwal, R., & Pramesh, C. S. (2015). Common pitfalls in statistical analysis: Odds versus risk. *Perspectives in clinical research*, *6*(4), 222–224. https://doi.org/10.4103/2229-3485.167092
