# Supplementary material for: Assessing the characteristics of un- and under-vaccinated children in low- and middle-income countries: A multi-level cross-sectional study
Source: PLOS Glob Public Health. 2022 Apr 27;2(4):e0000244. doi: 10.1371/journal.pgph.0000244 (PMC10021434; doi:10.1371/journal.pgph.0000244)
Supplement: Attachment — Submitted filename: Response_to_reviewers_commentsR1.docx [file pgph.0000244.s003.docx]

**Journal requirements:**

Please review your reference list to ensure that it is complete and correct. If you have cited papers that have been retracted, please include the rationale for doing so in the manuscript text, or remove these references and replace them with relevant current references. Any changes to the reference list should be mentioned in the rebuttal letter that accompanies your revised manuscript. If you need to cite a retracted article, indicate the article’s retracted status in the References list and also include a citation and full reference for the retraction notice.

**Response:** Thank you for this comment. We have cross-checked the reference list and we can confirm that this does not include any retracted article.

**Response to comments by Reviewer**

**Comment #1.** Assessing the characteristics of un- and under-vaccinated children in low- and middle-income countries: A multi-level cross-sectional study

The authors accepted most of the recommendations made in the first review, but some issues should be reviewed and discussed.

**Response:** Thank you for this comment. In our responses below, we have addressed the remaining issues raised. In general, we note that these comments did not require any amendments to the manuscript as we provided a clarification or a rebuttal where necessary.

**Comment #1** The uploaded supplementary file with the table and figures is a marked-up copy.

**Response:** Thanks for this observation. We have now uploaded an unmarked copy of the supplementary file.

**Comment #2** The authors are probably confusing two terms that are directly related, but that should be differentiated from a methodological point of view, including from an epidemiological and statistical perspective: study design (types of studies) and data analysis.

It is evident that the study and data analysis include both descriptive and inferential elements and I did not dispute these in the my previous review. A descriptive study (I mean, a study classified from a methodological and epidemiological point of view as a descriptive study) may include inferential statistical analysis, as performed in your work. In other words (and using your answer), “*these analyzes are not descriptive*” (logistic regression models to quantify the associations), but the study design is descriptive. I suggest you review these concepts. A good and classic Epidemiology literature containing study design and data analysis can be useful.

**Response:** Thank you for this comment. We would like to further clarify that our study design is not descriptive. Although we used cross-sectional data (studies utilizing cross-sectional data are often classified as descriptive studies, see Aggarwal and Ranganathan (2019)), the fact that we assessed the relationships between several covariates and indicators of vaccination coverage no longer makes our study design descriptive. This is well explained by Aggarwal and Ranganathan (2019), who first noted that a descriptive study “allows the researcher to study and describe the distribution of one or more variables, without regard to any causal or other hypotheses.” They then further clarified that cross-sectional study designs would no longer be “descriptive” but “analytical” if “the investigator also assesses the relationship between the presence of an exposure and that of an outcome”. This suggests that what makes a study design descriptive or not is not only the data that are used but also the statistical methods used to analyse the data, as we clarified in our previous response. In both of these areas (i.e. the data and the analytical approach used), our study does not qualify to be described as “descriptive”. This is particularly the case because Demographic and Health Surveys, though cross-sectional when considering each year separately, are inherently designed for inferential studies as most analyses involving such data are geared towards making inferences/testing some hypotheses about the populations from which the samples were drawn, as we have done in our study.

We further note that this comment does not require any changes to the manuscript since we have already addressed the main issue of changing the terms “risk” and “likelihood” to “odds” in the earlier comment.

**Comment #3**. Even though it is not the objective of the study ("Estimating coverage levels for the study countries was not one of the objectives of the study"), I emphasize that the results presented on page 6 (lines 193-204) should be moved to the Results.

When asked about this point in the first review, the authors responded and/or argued that "[...] *The figures/estimates mentioned here only serve to describe the data that we analyzed. Including summary statistics of the data to be analysed in the data section is common practice considering that these are not part of the results of our study*".

This comment disregards that the results related to the summary of DHS data and estimates of coverage vaccines referred as "*not one of the objectives of the study*" are not part of the exploratory data analysis, which is essential for the study's explicit objectives to be evaluated. Therefore, these results are directly related to the study objectives and should be relocated in the Results section.

Additionally, regarding to "*Including summary statistics of the data to be analyzed in the data section is common practice considering that these are not part of the results of our study*": do the authors have a reference that supports it? If applicable, please provide a reference on the presentation of exploratory analysis results in the Methodology section, rather than in the Results.

Being common doesn't mean it's adequate.

**Response:** Thank you once again for this comment. We would like to reiterate that the contents of Table 1 are not “results” in our study. Some of the data shown in the table are already included in DHS reports accompanying the various DHS datasets we analysed and are therefore not new.

Some of the authors of the manuscript come from a statistical background. It is quite common in many research papers (see, e.g., Mukhopadhyay and Sahu (2018)) to include some summary statistics of the data being analyzed in the Data section when describing it. We further note that the Table 1 in our manuscript can even be included in the introduction section, but we have chosen to present it in the data section as it fits better in this section. We do not see the point in moving it to the Results section. The data shown in the table are not part of the results of our study and we do not want these to detract from our main results. Thank you.

**References**

Aggarwal, R., and Ranganathan, P. (2019). Study designs: Part 2 - Descriptive studies. *Perspectives in clinical research*, *10*(1), 34–36. <https://doi.org/10.4103/picr.PICR_154_18>

Mukhopadhyay, S. and Sahu, S.K. (2018), A Bayesian spatiotemporal model to estimate long-term exposure to outdoor air pollution at coarser administrative geographies in England and Wales. J. R. Stat. Soc. A, 181: 465-486. <https://doi.org/10.1111/rssa.12299>
